# Supplementary material for: Deciphering regulatory architectures of bacterial promoters from synthetic expression patterns
Source: PLoS Comput Biol. 2024 Dec 26;20(12):e1012697. doi: 10.1371/journal.pcbi.1012697 (PMC11709304; doi:10.1371/journal.pcbi.1012697)
Supplement: S1 Appendix — (PDF) [file pcbi.1012697.s001.pdf]

S1 Appendix Models of the probabilities of microscopic states during transcription

For the purposes of this paper, the first step in the modeling of transcription is the identification of some set of microscopic states that represent the entire set of states available to the promoter dictating our gene of interest. Already, such models represent a coarse-graining of the complex microscopic reality of DNA and its attendant proteins within a cell. For example, DNA conformation at all scales (chromatin structure, supercoiling, etc.) is not included in the most naive application of these models. This kind of coarse-graining is consistent with the depiction of regulatory architectures as a region of DNA with some collection of binding sites and their allied transcription factors such as seen in RegulonDB or EcoCyc and represented in Fig 4 in the paper. Though the assumption of discrete states itself deserves further scrutiny, in this paper we accept these assumptions about a set of discrete states with no further discussion. Once we accept this constellation of microscopic states, many models focus on the steady-state probabilities of those states. Of course, the full dynamical trajectories of these different microstates are of great interest and one approach to computing those dynamics is the use of coupled chemical master equations. With the advent of experimental approaches to measuring the *dynamics* of transcription, the analysis of gene expression dynamics requires something more than is offered by the steady-state probabilities considered here.

In general, the steady-state probabilities of the different microstates can be written in the form

$$p_i(x_1, x_2, \cdots x_n) = \frac{P_i(x_1, x_2, \cdots x_n)}{Q(x_1, x_2, \cdots x_n)}, \tag{S1}$$

where here we examine the probability of the ith state as a rational function (i.e. a ratio of two polynomials) where x_j is the concentration of the jth transcription factor (or RNA polymerase) and with $Q(x_1, x_2, \cdots x_n)$ serving as a kind of generalized partition function gotten by summing over all states (or paths). In some instances, it is justified to specialize Eq S1 to the form

$$p_i(x) = \frac{P_i(x)}{Q(x)}, \tag{S2}$$

where for example our regulatory architecture of interest features only a single repressor or activator whose concentration is measured by x. If we think of the huge topic of input-output functions in biology, then $P_i(x)/Q(x)$ includes a representation of leakiness (the amount of output $p_i(x)$ even in the absence of input, $x = 0$), dynamic range, EC_{50} (the concentration at which the output reaches half its maximum) and the sensitivity as measured by the slope of the input-output curve (usually in logarithmic variables) at the midpoint [1].

In the present paper, we exploit two different ways of thinking about these rational functions, one of which is a subset of the other. In particular, the most general models we consider provide the steady-state probabilities using the tools of graph theory and make no assumption of a quasi-equilibrium [2, 3]. Indeed, such models are a convenient platform for exploring the precise consequences of breaking detailed balance associated with some kinetic step connecting two different microstates [4]. Within the study of transcriptional regulation, the more common class of useful models is sometimes referred to as "thermodynamic models," which have a deep and interesting history in the context of both test-tube biochemistry and the study of signaling, regulation and physiology within living organisms. Such models have played an important role as a conceptual framework for more than a century and a convenient and inspiring point of departure is the work of Archibald V Hill after whom the famed Hill function

$$p_{\text{bound}}(x) = \frac{\left(\frac{x}{K}\right)^n}{1 + \left(\frac{x}{K}\right)^n} \tag{S3}$$

is named. In this case, x is the concentration of some ligand and K is an effective dissociation constant. As Hill himself tells us, this functional form was hypothesized to describe the occupancy of hemoglobin by oxygen (the example he used, though it applies and has been applied much more broadly). Already more than a century ago, Hill argued of the function that now bears his name: "My object was rather to see whether an equation *of this type* can satisfy all the observations, than to base any direct physical meaning on

n and K." [5] He goes further in his 1913 paper noting "In point of fact n does not turn out to be a whole number, but this is due simply to the fact that aggregation is not into one particular type of molecule, but rather into a whole series of different molecules: so that equation (1) is a rough mathematical expression for the sum of several similar quantities with n equal to 1, 2, 3, 4 and possibly higher integers." [6]

In the subsequent decades, the equilibrium analysis of biochemical interactions became increasingly sophisticated with Pauling formulating a model that goes far beyond the Hill function by computing the average number of oxygen molecules bound to hemoglobin in the form

$$\langle N_{\mathrm{bound}}\rangle = \frac{4x + 12x^2 j + 12x^3 j^3 + 4x^4 j^6}{1 + 4x + 6x^2 j + 4x^3 j^3 + x^4 j^6}, \tag{S4}$$

where we adopt the simplifying notation $x = [O_2]$ [7, 8]. The parameter j is an interaction energy that imposes cooperativity in the sense that once one O_2 molecule is bound, the binding of the next one is easier. The equilibrium model of Adair went even further [9], positing that the average number of oxygen molecules bound to hemoglobin is given by

$$\langle N_{\mathrm{bound}}\rangle = \frac{4x + 12x^2 j + 12x^3 j^3 k + 4x^4 j^6 k^4 l}{1 + 4x + 6x^2 j + 4x^3 j^3 k + x^4 j^6 k^4 l}, \tag{S5}$$

where now the parameter k captures 3-body interactions between O_2 molecules and the parameter l captures 4-body interactions [7]. For more than a century, equilibrium thinking has suffused the study of biochemical reactions, even when promoted from the sterile setting of test-tube biochemistry to the messy world of hemoglobin molecules enclosed within red blood cells that are themselves cycling rapidly through the circulatory systems of animals ranging from high-flying birds such as the bar-headed geese to elite divers such as blue whales.

It was not a huge leap to go from the idea of examples such as oxygen binding to hemoglobin (a special case of receptor-ligand binding) to the idea of DNA itself as the receptor and various proteins such as transcription factors and RNA polymerase as the "ligand." Classic work from Ackers and Shea [10, 11] formalized the kind of "regulated recruitment" thinking that had been diligently pursued by Ptashne and coworkers [12], turning it into a formal mathematical structure for evaluating the state probabilities for various occupancies of a promoter of interest. More recently, these ideas have been developed deeply by a battery of researchers several examples of which are given here [13, 14, 15]. Similarly, right from the get-go, the study of gene regulation made it clear that the phenomenon of induction (i.e. the use of effector molecules to tune the state of expression) would require a quantitative description, and beautiful work in the 1960s articulated a wide range of different equilibrium allosteric models such as the MWC model [16], the KNF model [17] and the generalization of these ideas in the model of Manfred Eigen [18]. All of these models, even if no one explicitly says so, are "thermodynamic models" in that they provide a systematic protocol for using statistical mechanics to find the probabilities of *all* of the allowed states. Note that these different models differ not in whether they are quasi-equilibrium or not, but rather in which states they permit.

The vast majority of approaches to generating a specific functional form for state probabilities in transcription like those given in Eq S1 are either: (a) some version of a thermodynamic model which appeals in one way or another to states and Boltzmann weights, (b) phenomenological guesses in which some convenient functional form is adopted (usually a Hill function) and more rarely, (c) using the tools of non-equilibrium physics and graph theory, a version of p_i is adopted that reflects broken detailed balance. Approaches (a) and (c) both require some sort of mechanistic commitment about the classes of states that the system can adopt, and we note that (a) is a special case of (c). In the case of thermodynamic models, these polynomials have a very special form dictated by the Boltzmann weights of the different states of binding between transcription factors and their target DNA.

Part of the reason for the importance of this appendix is because there are so many different opinions in play on the subject of thermodynamic models in the biological setting in the literature. Part of the passion associated with the subject is that some authors are explicit in naming their work as thermodynamic or equilibrium models and others are not. Some fragment of the scientific population has an intrinsic belief that because "living organisms are out of equilibrium," thermodynamic or equilibrium models have no place. We think this extreme view can be replaced by a more nuanced perspective given that even within the fiery interior of a star, equilibrium ideas are used routinely and successfully (see the Saha equation). Rather, we

are going to highlight several alternative and more nuanced pictures including: (i) justification based upon separation of time scales, (ii) a null model which serves as a first and simplest regulatory hypothesis and (iii) phenomenology.

First, we consider the status of thermodynamic models as a null model for the steady-state probabilities of biochemical phenomena with special emphasis on the application to transcription. Then, we subject such models to the harshest scrutiny: what is their track record as a conceptual framework for thinking about experimental data, and what is the nature of their shortcomings? Here we use the title "thermodynamic models" as a shorthand to refer to *all* models of occupancy of transcription factors, nucleosomes and polymerases in which the state probabilities are obtained from the Boltzmann distribution, or some phenomenological approximation (i.e. Hill function) to the equilibrium state probabilities. These models are ubiquitous not only in the theory literature of transcription, but also as an interpretive null model for huge classes of experimental data. To give a flavor for the use of these models, we provide several key case studies followed by a smorgasbord of citations which the reader is urged to consult. Aside from providing references, we decided to forego our own extensive efforts at using and scrutinizing thermodynamic models because we wanted to highlight the ubiquitous nature of such thinking beyond our own work. The original theory work of Ackers and Shea [10, 11] focused primarily on the example of phage lambda, itself already introduced non-mathematically in thermodynamic model format by Ptashne and collaborators [12]. Perhaps no example is more famous than the bacterial example of the *lac* operon and its synthetic variants. Müller-Hill and Oehler and coworkers made an impressive series of quantitative and rigorous studies of synthetic variants of the *lac* operon which in modern parlance we would view as having "tuned the knobs" of transcription such as the strength of DNA binding sites for Lac repressor, the copy number of the Lac repressor and even the length of the DNA loop formed by binding two sites simultaneously, done with exquisite single-base pair precision [19, 20]. In a large number of papers, Vilar and Leibler [13] and subsequently, Saiz and Vilar [21, 22, 23] have provided a corresponding theoretical study of this data (and much more). Kuhlman et al. used the tools of molecular biology to construct strains of *E. coli* such that they could explicitly test thermodynamic models [24](which they expertly modeled using thermodynamic models following their own earlier theory work showing how thermodynamic models could be used to dissect logic gates [14]). Similar studies in the context of the *ara* operon by Schleif and co-workers provided a picture of how DNA looping can be treated quantitatively within the confines of thermodynamic models [25, 26, 27]. We are strong advocates for those cases in which ultimately models of transcription are confronted with well-designed experiments that tune the same knobs that were controlled in the theoretical models. Another example of this kind of regulatory dissection for bacterial promoters is offered by work on MarA which activates transcription [28, 29]. One reason for skepticism concerning these apparent successes is the possibility that in some cases non-equilibrium and equilibrium models will "agree" on some particular set of data. To distinguish them may involve tuning some knob that has not yet been tuned. This paragraph only scratches the surface of the vast array of work based upon these models. A more detailed sense of the reach of this work can be gleaned by looking at the hundreds of citations of papers such as those of Buchler, Gerland and Hwa [14], Bintu et al. [30, 31], and Sherman and Cohen [15].

One of the most important measures of the "success" of thermodynamics is in their power to unify apparently quite distinct data in the form of data collapse. Two extremely impressive examples of such data collapse were explored in the context of chemotaxis (see Fig 5 of Keymer et al. [32]) and quorum sensing (see Fig 6 of Swem et al. [33]), where in both cases the activity of a signaling pathway was modeled using the equilibrium MWC model of allosteric receptors. The key finding is that the receptor activity for an entire suite of mutants could be collapsed onto one single master curve in the same way that for simple ligand-receptor binding (Hill function with Hill coefficient $n = 1$), if we plot p_{bound} vs c/K_d rather than c, we find that all ligand-receptor curves fall on one universal curve. For the chemotaxis and quorum sensing examples, the data collapse is much more subtle. Though we cannot consider this a bulletproof demonstration that the thermodynamic models are "right," they certainly provide a powerful, unifying and parameter-free predictive framework for thinking about experiments. In the context of transcription, similar data collapse was achieved featuring a very demanding parameter-free collapse of a broad array of experimental data in which binding site strength, transcription factor copy number and gene copy number were systematically varied (see Fig 4 of Weinert et al. [34]) and for these same constructs as a function of inducer concentration (see Fig 7(B) of Razo et al. [1]).

Before briefly turning to a survey of some of the shortcomings of the thermodynamic models, we present

examples in which differential equations are used to model the dynamics of either mRNA or protein production and that implicitly feature thermodynamic models to describe gene regulation. Our key point here is to note that often these equations take the form (for example, Eq 5 in Cherry and Adler [35])

$$\frac{\mathrm{d}A}{\mathrm{d}t} = -\gamma A + f_{\text{production}}(A), \tag{S6}$$

where almost always the production term can be written in the form

$$f_{\text{production}}(A) = \frac{P(A)}{Q(A)}, \tag{S7}$$

where $P(A)$ and $Q(A)$ are polynomials. Further, in most instances, these rational functions are either of the phenomenological (or quasi-equilibrium) Hill form or appeal directly to Boltzmann states and weights. To be concrete, in two of the classic examples of synthetic biology, the genetic switch and the repressilator, the dynamical models were of the form described above [36, 37]. For example, for the genetic switch the production of the two species of repressor are written in dimensionless form (see Eq 1a and Eq 1b of Gardner et al. [36]) as

$$\begin{aligned} \frac{\mathrm{d}r_1}{\mathrm{d}\tau} &= -r_1 + \frac{\alpha}{1 + r_2^n}, \\ \frac{\mathrm{d}r_2}{\mathrm{d}\tau} &= -r_2 + \frac{\alpha}{1 + r_1^n}. \end{aligned} \tag{S8}$$

Here r_1 and r_2 are the dimensionless concentrations of the two mutually repressing repressors, time is measured in units of $\tau = \gamma t$ where γ is the degradation rate and α is a dimensionless protein production rate. Our main point here is to note that although the words "thermodynamic model" or "equilibrium" are never used, the right-hand side of these equations is explicitly computing the probability of binding site occupancy by repressors.

We are hopeful that the subject of transcription will be held to the highest quantitative standards. We are excited by the many examples highlighted here (which only scratches the surface) and hope for a time when we have a deep and predictive understanding of all the genes in key model organisms and that these insights will serve as the basis for the study of non-model organisms such as redwood trees and blue whales. One route to such predictive understanding is the critical scrutiny offered by a dialogue between theory and experiment. In this paragraph, we note in passing a number of examples where it appears that the thermodynamic null models do not pass muster. One subject of intense effort over the last few decades is the study of DNA packing in eukaryotes and its implications for gene expression. As usual, the literature of this topic is immense. Intense debates have unfolded on the position of nucleosomes on genomic DNA with the conclusion likely that equilibrium models by themselves will *not* explain all the extant data [38, 39, 40]. The connection between expression and nucleosomal occupancy has been taken farther recently using single-cell methods with the result that the thermodynamic null model must be superseded by a more detailed model [41]. Similarly, in the context of the *Pho5* promoter in yeast, a quite amazing set of experiments was done to measure the occupancies of nucleosomes on this promoter. This data was analyzed using tens of thousands of models and the only models consistent with all of the data appear to require broken detailed balance [42, 43]. In a beautiful use of the MWC model highlighted above, Mirny worked out the probability that nucleosomes will be present essentially regulating some gene of interest and even went so far as to make an analogy with the Bohr effect in hemoglobin in which the post-translational modification of nucleosomes could be thought of as a kind of Bohr effect [44]. Though it took nearly a decade, it appears that this model is not sufficient to explain chromatin accessibility and gene expression [45]. We note that this example is common: to really make the comparison between theory and experiment often means that the data that exists is just not quite right to make the acid test (see the example of hemoglobin where, in our view, misuses of the MWC model led to the conclusion that the "model doesn't work" whereas in reality, it was rather that the set of states needed to be expanded to include other effectors [46, 47, 45]). This is also carefully explained in Chapter 7 of Ref. [48]. However, failure of the thermodynamic framework reaches well beyond the example of chromatin where it is clear that energy-consuming processes such as nucleosome remodeling can break detailed balance. Beautiful single-molecule experiments in *E. coli* revealed that even in

the process of transcription factor binding to its DNA target, the results were not consistent with the thermodynamic framework [49]. Similarly, application of the thermodynamic modeling framework in the context of the *Pseudomonas aeruginosa* genes associated with the transcription factor BqsR have thus far been unsuccessful [50]. One particularly interesting example of the shortcomings of the thermodynamic approach was in the context of the glucocorticoid receptor where it was found that the rank ordering of gene expression did not scale with the rank ordering of binding site strength [51]. As with our list of "successes" this list of failures of the thermodynamic framework is superficial and incomplete. Further, we argue that to actually make claims of "right" or "wrong" requires diligent and often frustrating dialogue between theory and experiment where dedicated efforts need to be made to make sure that the same knobs are being tuned in both the theory and the corresponding experiments.

In summary, we would put the place of thermodynamic models in thinking about in vivo biochemistry (including the case of transcription) on par with other seemingly naive but incredibly productive null models such as the apparently crazy idea of a noninteracting electron gas to describe metals or the Ising model as a way to describe magnetic phenomena. In the context of transcription, though it is routine to critique thermodynamic models, we would argue that in fact, the thermodynamic models are just as plausible as the equally popular "two-state promoter" used so often to explain noise in transcription. Several beautiful examples of the use of the two-state framework are given in So at al. and Zenklusen et al. [52, 53]. For the purposes of the present paper, the thermodynamic model framework is a convenient null model which allows us to self-consistently generate synthetic datasets that can then be analyzed by the tools used to study real data with the added benefit that we "know the answer" from the outset. This appendix is a very superficial rendering of a vast subject. We hope at the least that it provides an entry into the literature which has used the so-called thermodynamic models and that attempts a balanced view of their successes and shortcomings. In our view, ultimately, the status of all models of transcription will only be really clarified by a painstaking dialogue between theory and experiment.

SI references

1. Razo-Mejia M, Barnes SL, Belliveau NM, Chure G, Einav T, Lewis M, and Phillips R. Tuning Transcriptional Regulation through Signaling: A Predictive Theory of Allosteric Induction. Cell Syst 2018 Apr; 6:456–469.e10
2. Gunawardena J. A linear framework for time-scale separation in nonlinear biochemical systems. PLoS One 2012 May; 7:e36321
3. Ahsendorf T, Wong F, Eils R, and Gunawardena J. A framework for modelling gene regulation which accommodates non-equilibrium mechanisms. BMC Biol. 2014 Dec; 12:102
4. Mahdavi SD, Salmon GL, Daghlian P, Garcia HG, and Phillips R. Flexibility and sensitivity in gene regulation out of equilibrium. Proc. Natl. Acad. Sci. U. S. A. 2024 Nov; 121:e2411395121
5. Hill A and Paganini-Hill A. The possible effects of the aggregation of the molecules of haemoglobin on its dissociation curves. J. Physiol. 1910
6. Hill AV. The Combinations of Haemoglobin with Oxygen and with Carbon Monoxide. I. Biochem. J 1913 Oct; 7:471–80
7. Phillips R, Kondev J, Theriot J, and Garcia H. Physical Biology of the Cell. Garland Science, 2012
8. Pauling L. The Oxygen Equilibrium of Hemoglobin and Its Structural Interpretation. Proc. Natl. Acad. Sci. U. S. A. 1935 Apr; 21:186–91
9. Adair GS, Bock AV, and Field H. The hemoglobin system: VI. The oxygen dissociation curve of hemoglobin. J. Biol. Chem. 1925 Mar; 63:529–45
10. Ackers GK, Johnson AD, and Shea MA. Quantitative model for gene regulation by lambda phage repressor. Proc. Natl. Acad. Sci. U. S. A. 1982 Feb; 79:1129–33
11. Shea MA and Ackers GK. The OR control system of bacteriophage lambda. A physical-chemical model for gene regulation. J. Mol. Biol. 1985 Jan; 181:211–30

12. Ptashne M. A Genetic Switch: Phage Lambda Revisited. New York: Cold Spring Harbor Laboratory Press, 2004
13. Vilar JMG and Leibler S. DNA looping and physical constraints on transcription regulation. J. Mol. Biol. 2003 Aug; 331:981–9
14. Buchler NE, Gerland U, and Hwa T. On schemes of combinatorial transcription logic. Proc. Natl. Acad. Sci. U. S. A. 2003 Apr; 100:5136–41
15. Sherman MS and Cohen BA. Thermodynamic state ensemble models of cis-regulation. PLoS Comput. Biol. 2012 Mar; 8:e1002407
16. Monod J, Wyman J, and Changeux JP. On the nature of allosteric transitions: A plausible model. J. Mol. Biol. 1965 May; 12:88–118
17. Koshland Jr DE, Némethy G, and Filmer D. Comparison of experimental binding data and theoretical models in proteins containing subunits. Biochemistry 1966 Jan; 5:365–85
18. Eigen M. New looks and outlooks on physical enzymology. Q. Rev. Biophys. 1968 May; 1:3–33
19. Oehler S, Amouyal M, Kolkhof P, Wilcken-Bergmann B von, and Müller-Hill B. Quality and position of the three lac operators of *E. coli* define efficiency of repression. EMBO J. 1994 Jul; 13:3348–55
20. Oehler S, Alberti S, and Müller-Hill B. Induction of the lac promoter in the absence of DNA loops and the stoichiometry of induction. Nucleic Acids Res. 2006 Jan; 34:606–12
21. Vilar JMG and Saiz L. DNA looping in gene regulation: from the assembly of macromolecular complexes to the control of transcriptional noise. Curr. Opin. Genet. Dev. 2005 Apr; 15:136–44
22. Vilar JMG and Saiz L. Systems biophysics of gene expression. Biophys. J. 2013 Jun; 104:2574–85
23. Vilar JMG and Saiz L. The unreasonable effectiveness of equilibrium gene regulation through the cell cycle. bioRxiv 2023 Apr :2023.03.31.535089
24. Kuhlman T, Zhang Z, Saier Jr MH, and Hwa T. Combinatorial transcriptional control of the lactose operon of *Escherichia coli*. Proc. Natl. Acad. Sci. U. S. A. 2007 Apr; 104:6043–8
25. Dunn TM, Hahn S, Ogden S, and Schleif RF. An operator at -280 base pairs that is required for repression of araBAD operon promoter: addition of DNA helical turns between the operator and promoter cyclically hinders repression. Proc. Natl. Acad. Sci. U. S. A. 1984 Aug; 81:5017–20
26. Ogden S, Haggerty D, Stoner CM, Kolodrubetz D, and Schleif R. The *Escherichia coli* L-arabinose operon: binding sites of the regulatory proteins and a mechanism of positive and negative regulation. Proc. Natl. Acad. Sci. U. S. A. 1980 Jun; 77:3346–50
27. Schleif R and Lis JT. The regulatory region of the L-arabinose operon: a physical, genetic and physiological study. J. Mol. Biol. 1975 Jul; 95:417–31
28. Martin RG, Bartlett ES, Rosner JL, and Wall ME. Activation of the *Escherichia coli* marA/soxS/rob regulon in response to transcriptional activator concentration. J. Mol. Biol. 2008 Jul; 380:278–84
29. Wall ME, Markowitz DA, Rosner JL, and Martin RG. Model of transcriptional activation by MarA in *Escherichia coli*. PLoS Comput. Biol. 2009 Dec; 5:e1000614
30. Bintu L, Buchler NE, Garcia HG, Gerland U, Hwa T, Kondev J, and Phillips R. Transcriptional regulation by the numbers: models. Curr. Opin. Genet. Dev. 2005 Apr; 15:116–24
31. Bintu L, Buchler NE, Garcia HG, Gerland U, Hwa T, Kondev J, Kuhlman T, and Phillips R. Transcriptional regulation by the numbers: applications. Curr. Opin. Genet. Dev. 2005 Apr; 15:125–35
32. Keymer JE, Endres RG, Skoge M, Meir Y, and Wingreen NS. Chemosensing in *Escherichia coli*: two regimes of two-state receptors. Proc. Natl. Acad. Sci. U. S. A. 2006 Feb; 103:1786–91
33. Swem LR, Swem DL, Wingreen NS, and Bassler BL. Deducing receptor signaling parameters from in vivo analysis: LuxN/AI-1 quorum sensing in Vibrio harveyi. Cell 2008 Aug; 134:461–73
34. Weinert FM, Brewster RC, Rydenfelt M, Phillips R, and Kegel WK. Scaling of gene expression with transcription-factor fugacity. Phys. Rev. Lett. 2014 Dec; 113:258101
35. Cherry JL and Adler FR. How to make a biological switch. J. Theor. Biol. 2000 Mar; 203:117–33

36. Gardner TS, Cantor CR, and Collins JJ. Construction of a genetic toggle switch in *Escherichia coli*. Nature 2000 Jan; 403:339–42

37. Elowitz MB and Leibler S. A synthetic oscillatory network of transcriptional regulators. Nature 2000 Jan; 403:335–8

38. Segal E, Fondufe-Mittendorf Y, Chen L, Thåström A, Field Y, Moore IK, Wang JPZ, and Widom J. A genomic code for nucleosome positioning. Nature 2006 Aug; 442:772–8

39. Struhl K and Segal E. Determinants of nucleosome positioning. Nat. Struct. Mol. Biol. 2013 Mar; 20:267–73

40. Kaplan N, Moore IK, Fondufe-Mittendorf Y, Gossett AJ, Tillo D, Field Y, LeProust EM, Hughes TR, Lieb JD, Widom J, and Segal E. The DNA-encoded nucleosome organization of a eukaryotic genome. Nature 2009 Mar; 458:362–6

41. Doughty BR, Hinks MM, Schaepe JM, Marinov GK, Thurm AR, Rios-Martinez C, Parks BE, Tan Y, Marklund E, Dubocanin D, Bintu L, and Greenleaf WJ. Single-molecule chromatin configurations link transcription factor binding to expression in human cells. bioRxiv 2024 Feb :2024.02.02.578660

42. Brown CR, Mao C, Falkovskaia E, Jurica MS, and Boeger H. Linking stochastic fluctuations in chromatin structure and gene expression. PLoS Biol. 2013 Aug; 11:e1001621

43. Wolff MR, Schmid A, Korber P, and Gerland U. Effective dynamics of nucleosome configurations at the yeast PHO5 promoter. Elife 2021 Mar; 10

44. Mirny LA. Nucleosome-mediated cooperativity between transcription factors. Proc. Natl. Acad. Sci. U. S. A. 2010 Dec; 107:22534–9

45. Eck E, Liu J, Kazemzadeh-Atoufi M, Ghoreishi S, Blythe SA, and Garcia HG. Quantitative dissection of transcription in development yields evidence for transcription-factor-driven chromatin accessibility. Elife 2020 Oct; 9

46. Yonetani T, Park SI, Tsuneshige A, Imai K, and Kanaori K. Global allostery model of hemoglobin. Modulation of O(2) affinity, cooperativity, and Bohr effect by heterotropic allosteric effectors. J. Biol. Chem. 2002 Sep; 277:34508–20

47. Rapp O and Yifrach O. Using the MWC model to describe heterotropic interactions in hemoglobin. PLoS One 2017 Aug; 12:e0182871

48. Phillips R. The Molecular Switch: Signaling and Allostery. Princeton University Press, 2020

49. Hammar P, Walldén M, Fange D, Persson F, Baltekin O, Ullman G, Leroy P, and Elf J. Direct measurement of transcription factor dissociation excludes a simple operator occupancy model for gene regulation. Nat. Genet. 2014 Apr; 46:405–8

50. Kreamer NN, Phillips R, Newman DK, and Boedicker JQ. Predicting the impact of promoter variability on regulatory outputs. Sci. Rep. 2015 Dec; 5:18238

51. Meijsing SH, Pufall MA, So AY, Bates DL, Chen L, and Yamamoto KR. DNA binding site sequence directs glucocorticoid receptor structure and activity. Science 2009 Apr; 324:407–10

52. So LH, Ghosh A, Zong C, Sepúlveda LA, Segev R, and Golding I. General properties of transcriptional time series in Escherichia coli. Nat. Genet. 2011 Jun; 43:554–60

53. Zenklusen D, Larson DR, and Singer RH. Single-RNA counting reveals alternative modes of gene expression in yeast. Nat. Struct. Mol. Biol. 2008 Dec; 15:1263–71
