# Supplementary material for: Deciphering regulatory architectures of bacterial promoters from synthetic expression patterns
Source: PLoS Comput Biol. 2024 Dec 26;20(12):e1012697. doi: 10.1371/journal.pcbi.1012697 (PMC11709304; doi:10.1371/journal.pcbi.1012697)
Supplement: S2 Appendix — (PDF) [file pcbi.1012697.s002.pdf]

S2 Appendix Predicting the probability of RNA polymerase being bound using thermodynamic models

S2.1 Protocol to derive the thermodynamic models

To estimate the expression levels of a gene with a given promoter, one key step is to calculate the probability of RNA polymerase (RNAP) being bound, which is needed in computing the rate of mRNA production using Eq 3. This can be done by building thermodynamic models of transcriptional regulation. In this appendix, we outline the protocol for building thermodynamic models, using a promoter that is constitutively expressed and a promoter with the simple repression architecture as examples.

On a broader level, the first step to build a thermodynamic model involves abstracting the genome into discrete microstates. As shown in Fig S1(A), our approach entails disregarding the three-dimensional topology of the genome and conceptualizing it as a linear sequence of discrete binding sites. RNAPs and transcription factors can bind to these sites in a number of configurations, each of which can be defined as a microstate. With this way of defining the microstates, we can then write down a general protocol for building thermodynamic models, which is shown in Fig S1(B). First, we need to identify all the relevant promoter states. Second, we compute the energies for each of the states. Next, we need to calculate the multiplicity for each of the states. This is needed because each of the states encompass a range of possible configurations, and we need to count the total number of configurations associated with each state. Finally, we can compute the statistical weight of each state, which is the product of multiplicity and the Boltzmann weight calculated using the Boltzmann law of statistical mechanics. The statistical weight is in the form of

$$\omega_i = W\, e^{-\beta \varepsilon_i}, \tag{S9}$$

where W is the multiplicity, $\beta = 1/k_B T$ with k_B representing the Boltzmann constant and T representing temperature, and ε_i is the energy of the i-th state. The probability of any given state is then calculated by dividing ω_i by the partition function, which is the sum of the weights of all states.

To demonstrate how this protocol is used, let us consider a constitutive promoter, which is not regulated by any transcription factor. As shown in Fig S1(B), there are two possible states of binding: the state where one RNAP is bound to the promoter and the state where the promoter is empty. In the latter state, all RNAPs are bound to a non-specific binding site along the rest of the genome. Let us suppose that the specific binding energy at the promoter is $\varepsilon_{\text{pd}}^{\text{S}}$ and the non-specific binding energy along the rest of the genome is $\varepsilon_{\text{pd}}^{\text{NS}}$. If there are P molecules of RNAPs in the system, then the energy of the empty promoter is $P\varepsilon_{\text{pd}}^{\text{NS}}$ and the energy when one RNAP is bound to the promoter and the remaining $(P-1)$ RNAPs are bound non-specifically is $\varepsilon_{\text{pd}}^{\text{S}} + (P-1)\varepsilon_{\text{pd}}^{\text{NS}}$.

Following the protocol, we next find the multiplicity of each promoter state. Let N_{NS} be the number of non-specific binding sites, then the multiplicity of the empty promoter state is given by

$$W_{\text{NS}}(P, N_{\text{NS}}) = \frac{N_{\text{NS}}!}{P!(N_{\text{NS}} - P)!} \approx \frac{(N_{\text{NS}})^P}{P!}. \tag{S10}$$

The approximation in the second step holds true because N_{NS} is typically taken to be the length of the genome, which is on the order of 10^6 for *E. coli.* Therefore, we have that $N_{\text{NS}} \gg P$ and $\frac{N_{\text{NS}}!}{(N_{\text{NS}}-P)!} \approx (N_{\text{NS}})^P$. We can use the same procedure to count the number of configurations associated with the state where RNAP is bound to the promoter. Since one RNAP molecule is bound to the promoter, there remain $(P-1)$ RNAP molecules that can bind to the non-specific binding sites in the rest of the genome. Therefore, the multiplicity of the RNAP-bound state is given by

$$W_{\text{S}}(P-1, N_{\text{NS}}) = \frac{N_{\text{NS}}!}{(P-1)!(N_{\text{NS}} - (P-1))!} \approx \frac{(N_{\text{NS}})^{P-1}}{(P-1)!}. \tag{S11}$$

Having written down the energies and multiplicity terms of the two promoter states, we can compute the statistical weights of the states, which are given in the fourth column on Fig S1(B). Now we're ready to write

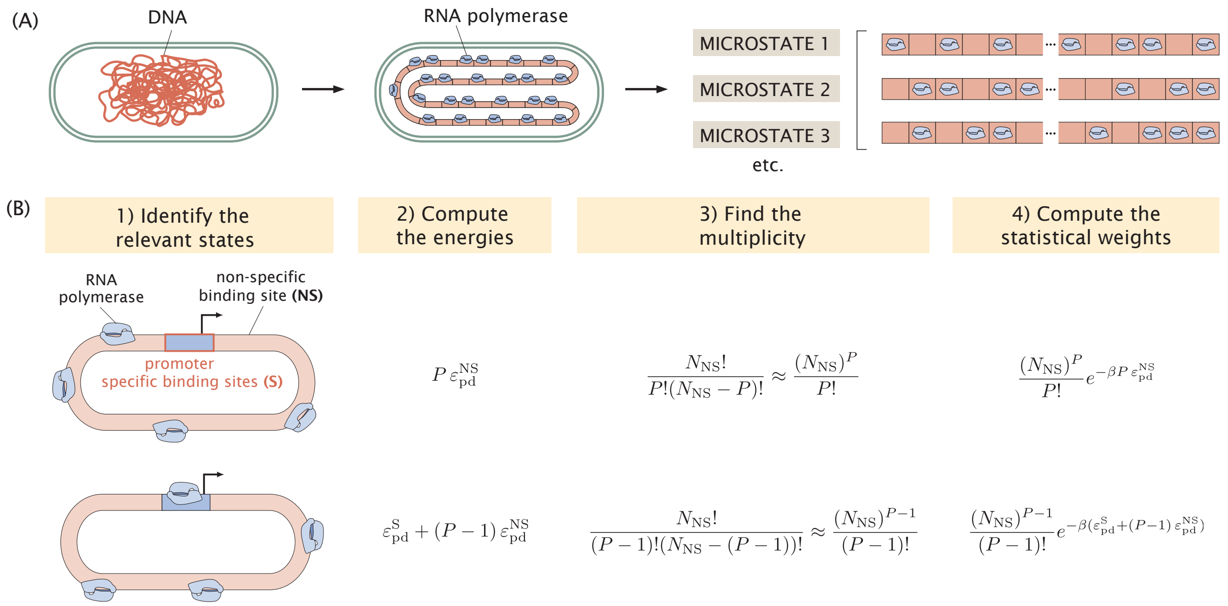

Fig S1. Writing down thermodynamic models for transcriptional regulation.
(A) Conceptualizing binding events along the genome as microstates. Each of the red boxes is a site along the genome to which RNAP and transcription factors can bind. Each of the possible configurations of binding is considered a microstate. (B) Protocol for writing down a thermodynamic model. The protocol involves four important steps. Firstly, all the relevant promoter states need to be identified. Secondly, the energy of each state is computed. Next, we compute the multiplicity, which tells us the number of possible configurations associated with each state. Finally, statistical weights are written down based on the energies and the multiplicity terms. For the energy terms ε, the superscript NS refers to non-specific binding, the superscript S refers to specific binding, and the subscript pd refers to RNAP (p) binding to DNA (d).

down the probability of RNAP being bound for a constitutive promoter

$$p_{\text{bound}} = \frac{\frac{(N_{\text{NS}})^{P-1}}{(P-1)!} e^{-\beta\varepsilon_{\text{pd}}^{\text{S}}} e^{-\beta(P-1)\varepsilon_{\text{pd}}^{\text{NS}}}}{\frac{(N_{\text{NS}})^{P}}{P!} e^{-\beta P\varepsilon_{\text{pd}}^{\text{NS}}} + \frac{(N_{\text{NS}})^{P-1}}{(P-1)!} e^{-\beta\varepsilon_{\text{pd}}^{\text{S}}} e^{-\beta(P-1)\varepsilon_{\text{pd}}^{\text{NS}}}}, \tag{S12}$$

where $\varepsilon_{\text{pd}}^{\text{S}}$ is the binding energy of RNAP at the promoter and $\varepsilon_{\text{pd}}^{\text{NS}}$ is the binding energy of RNAP at the non-specific binding sites. In particular, we assume that the binding energy is same across all non-specific binding sites. To simplify the expression, we can multiply all the terms in the numerator and the denominator by $\frac{P!}{(N_{\text{NS}})^P} e^{\beta P\varepsilon_{\text{pd}}^{\text{NS}}}$. This gives us

$$p_{\text{bound}} = \frac{\frac{P}{N_{\text{NS}}} e^{-\beta\Delta\varepsilon_{\text{pd}}}}{1 + \frac{P}{N_{\text{NS}}} e^{-\beta\Delta\varepsilon_{\text{pd}}}}, \tag{S13}$$

where $\Delta\varepsilon_{\text{pd}} = \varepsilon_{\text{pd}}^{\text{S}} - \varepsilon_{\text{pd}}^{\text{NS}}$ is the binding energy of the RNAP at the promoter relative to the binding energy at the non-specific binding sites.

This protocol can be easily extended to cases where the promoter is regulated by transcription factors. The simplest architecture that involves a transcription factor is where the promoter is regulated by a single repressor. As shown in Fig S2(B), for a promoter with the simple repression regulatory architecture, there are three possible state of binding: the state with an empty promoter, the state where RNAP is bound to the promoter, and the state where the repressor is bound to the promoter. Following the protocol above, we can

write down the following statistical weights for each of the three states

$$Z_{\text{empty promoter}} = \frac{N_{\text{NS}}!}{P!R!(N_{\text{NS}} - P - R)!} e^{-\beta P \varepsilon_{\text{pd}}^{\text{NS}}} e^{-\beta R \varepsilon_{\text{rd}}^{\text{NS}}} \tag{S14}$$

$$\approx \frac{(N_{\text{NS}})^P}{P!} \frac{(N_{\text{NS}})^R}{R!} e^{-\beta P \varepsilon_{\text{pd}}^{\text{NS}}} e^{-\beta R \varepsilon_{\text{rd}}^{\text{NS}}} \tag{S15}$$

$$Z_{\text{RNAP on promoter}} = \frac{N_{\text{NS}}!}{(P-1)!R!(N_{\text{NS}} - (P-1) - R)!} e^{-\beta (P-1) \varepsilon_{\text{pd}}^{\text{NS}}} e^{-\beta R \varepsilon_{\text{rd}}^{\text{NS}}} e^{-\beta \varepsilon_{\text{pd}}^{\text{S}}} \tag{S16}$$

$$\approx \frac{(N_{\text{NS}})^{P-1}}{(P-1)!} \frac{(N_{\text{NS}})^R}{R!} e^{-\beta (P-1) \varepsilon_{\text{pd}}^{\text{NS}}} e^{-\beta R \varepsilon_{\text{rd}}^{\text{NS}}} e^{-\beta \varepsilon_{\text{pd}}^{\text{S}}} \tag{S17}$$

$$Z_{\text{repressor on promoter}} = \frac{N_{\text{NS}}!}{P!(R-1)!(N_{\text{NS}} - P - (R-1))!} e^{-\beta P \varepsilon_{\text{pd}}^{\text{NS}}} e^{-\beta (R-1) \varepsilon_{\text{rd}}^{\text{NS}}} e^{-\beta \varepsilon_{\text{rd}}^{\text{S}}} \tag{S18}$$

$$\approx \frac{(N_{\text{NS}})^P}{P!} \frac{(N_{\text{NS}})^{R-1}}{(R-1)!} e^{-\beta P \varepsilon_{\text{pd}}^{\text{NS}}} e^{-\beta (R-1) \varepsilon_{\text{rd}}^{\text{NS}}} e^{-\beta \varepsilon_{\text{rd}}^{\text{S}}} \tag{S19}$$

where N_{NS} is the number of non-specific binding sites; P is the number of RNAPs; R is the number of repressors; $\Delta\varepsilon_{\text{pd}}$ is the binding energy of the RNAP; $\varepsilon_{\text{pd}}^{\text{S}}$ and $\varepsilon_{\text{pd}}^{\text{NS}}$ are the specific binding energy of the RNAP at the promoter and the binding energy of the RNAP at the non-specific binding site; $\varepsilon_{\text{rd}}^{\text{S}}$ and $\varepsilon_{\text{rd}}^{\text{NS}}$ are the specific binding energy of the repressor at the promoter and the binding energy of the repressor at the non-specific binding site. This allows us to write down the probability of RNAP binding as

$$p_{\text{bound}} = \frac{Z_{\text{RNAP on promoter}}}{Z_{\text{empty promoter}} + Z_{\text{RNAP on promoter}} + Z_{\text{repressor on promoter}}}. \tag{S20}$$

Again, we simplify the expression by multiplying both the numerator and the denominator by $\frac{P!}{(N_{\text{NS}})^P} \frac{R!}{(N_{\text{NS}})^R} e^{\beta \varepsilon_{\text{pd}}^{\text{NS}}} e^{\beta \varepsilon_{\text{rd}}^{\text{NS}}}$. This gives us

$$p_{\text{bound}} = \frac{\frac{P}{N_{\text{NS}}} e^{-\beta \Delta\varepsilon_{\text{pd}}}}{1 + \frac{P}{N_{\text{NS}}} e^{-\beta \Delta\varepsilon_{\text{pd}}} + \frac{R}{N_{\text{NS}}} e^{-\beta \Delta\varepsilon_{\text{rd}}}}, \tag{S21}$$

where $\Delta\varepsilon_{\text{pd}} = \varepsilon_{\text{pd}}^{\text{S}} - \varepsilon_{\text{pd}}^{\text{NS}}$ and $\Delta\varepsilon_{\text{rd}} = \varepsilon_{\text{rd}}^{\text{S}} - \varepsilon_{\text{rd}}^{\text{NS}}$ are the binding energies of the RNAP and the repressors at the promoter relative to their non-specific binding energies. Here, the weak promoter approximation is often made, which states that the RNAP binding state has a much lower Boltzmann weight compared to the repressor binding site. Therefore, the expression can often be simplified to

$$p_{\text{bound}} = \frac{\frac{P}{N_{\text{NS}}} e^{-\beta \Delta\varepsilon_{\text{pd}}}}{1 + \frac{R}{N_{\text{NS}}} e^{-\beta \Delta\varepsilon_{\text{rd}}}}. \tag{S22}$$

It is important to note that when the expressions for p_{bound} are used to predict the expression levels of promoter variants in an MPRA library, the energy terms ε_i are calculated for each promoter variant by mapping binding site sequences to energy matrices. The procedure for calculating the total energies is explained in Sec 1.1 and illustrated in Fig 3(A).

S2.2 States-and-weights models for common regulatory architectures

There are six common regulatory architectures for promoters in *E. coli*. In S2.1, we have written down p_{bound}, the probability that the RNAP is bound to the promoter, for a constitutively expressed promoter and a promoter with the simple repression regulatory architecture. Based on the states-and-weights diagrams shown in Fig S2 and using the same protocol introduced in S2.1, we can write down p_{bound} the remaining four common regulatory architectures [1].

For a promoter with the simple activation regulatory architecture, the states-and-weights diagram is shown in Fig S2(C), and the probability of RNAP being bound is given by

$$p_{\text{bound}} = \frac{\frac{P}{N_{\text{NS}}} e^{-\beta \Delta\varepsilon_{\text{pd}}} + \frac{P}{N_{\text{NS}}} \frac{A}{N_{\text{NS}}} e^{-\beta(\Delta\varepsilon_{\text{pd}} + \Delta\varepsilon_{\text{ad}})} \omega_{\text{ap}}}{1 + \frac{P}{N_{\text{NS}}} e^{-\beta \Delta\varepsilon_{\text{pd}}} + \frac{A}{N_{\text{NS}}} e^{-\beta \Delta\varepsilon_{\text{rd}}} + \frac{P}{N_{\text{NS}}} \frac{A}{N_{\text{NS}}} e^{-\beta(\Delta\varepsilon_{\text{pd}} + \Delta\varepsilon_{\text{ad}})} \omega_{\text{ap}}}, \tag{S23}$$

where $N_{\rm NS}$ is the number of non-specific binding sites; P is the number of RNAP; A is the number of activators; $\Delta\varepsilon_{\rm pd}$ is the binding energy of the RNAP; $\Delta\varepsilon_{\rm ad}$ is the binding energy of the activator; $\omega_{\rm a_1a_2}$ is the interaction energy between the activator and the RNAP.

For a promoter with the repression-activation regulatory architecture, the states-and-weights diagram is shown in Fig S2(D), and the probability of RNAP being bound is given by

$$p_{\rm bound} = \frac{\frac{P}{N_{\rm NS}}e^{-\beta\Delta\varepsilon_{\rm pd}} + \frac{P}{N_{\rm NS}}\frac{A}{N_{\rm NS}}e^{-\beta(\Delta\varepsilon_{\rm pd}+\Delta\varepsilon_{\rm ad})}\omega_{\rm ap}}{1+\frac{P}{N_{\rm NS}}e^{-\beta\Delta\varepsilon_{\rm pd}} + \frac{R}{N_{\rm NS}}e^{-\beta\Delta\varepsilon_{\rm rd}} + \frac{A}{N_{\rm NS}}e^{-\beta\Delta\varepsilon_{\rm ad}} + \frac{P}{N_{\rm NS}}\frac{A}{N_{\rm NS}}e^{-\beta(\Delta\varepsilon_{\rm pd}+\Delta\varepsilon_{\rm ad})}\omega_{\rm ap}}, \tag{S24}$$

where $N_{\rm NS}$ is the number of non-specific binding sites; P is the number of RNAPs; R is the number of repressors; A is the number of activators; $\Delta\varepsilon_{\rm pd}$ is the binding energy of the RNAP; $\Delta\varepsilon_{\rm rd}$ is the binding energy of the repressor; $\Delta\varepsilon_{\rm ad}$ is the binding energy of the activator; $\omega_{\rm a_1a_2}$ is the interaction energy between the activator and the RNAP.

Let $r_1 = \frac{R_1}{N_{\rm NS}}e^{-\beta\Delta\varepsilon_{r_1d}}$, $r_2 = \frac{R_2}{N_{\rm NS}}e^{-\beta\Delta\varepsilon_{r_2d}}$, and $p = \frac{P}{N_{\rm NS}}e^{-\beta\Delta\varepsilon_{pd}}$. Then, for a promoter with the double repression regulatory architecture under OR logic, the states-and-weights diagram is shown in Fig S2(E), and the probability of RNAP being bound is given by

$$p_{\rm bound} = \frac{p}{1+r_1+r_2+r_1r_2\omega_{\rm r_1r_2}+p}, \tag{S25}$$

where $N_{\rm NS}$ is the number of non-specific binding sites; P is the number of RNAPs; R_1 is the number of the first repressor; R_2 is the number of the second repressor; $\Delta\varepsilon_{\rm pd}$ is the binding energy of the RNAP; $\Delta\varepsilon_{\rm r_1d}$ is the binding energy of the first repressor; $\Delta\varepsilon_{\rm r_2d}$ is the binding energy of the second repressor; $\omega_{\rm r_1r_2}$ is the interaction energy between the two repressors. On the other hand, for a double repression promoter with AND logic, we need to consider two additional states where the RNAP is bound while one of the two repressors, but not both, is also bound. In this case, the probability of RNAP being bound is given by

$$p_{\rm bound} = \frac{p+r_1p+r_2p}{1+r_1+r_2+r_1r_2\omega_{\rm r_1r_2}+p+r_1p+r_2p}, \tag{S26}$$

Let $a_1 = \frac{A_1}{N_{\rm NS}}e^{-\beta\Delta\varepsilon_{a_1d}}$, $a_2 = \frac{A_2}{N_{\rm NS}}e^{-\beta\Delta\varepsilon_{a_2d}}$, and $p = \frac{P}{N_{\rm NS}}e^{-\beta\Delta\varepsilon_{pd}}$. Then, for a promoter with the double repression regulatory architecture under OR logic, the states-and-weights diagram is shown in Fig S2(F), and the probability of RNAP being bound is given by

$$p_{\rm bound} = \frac{p+a_1p\omega_{a_1p}+a_2p\omega_{a_2p}+a_1a_2p\omega_{a_1p}\omega_{a_2p}}{1+a_1+a_2+p+a_1a_2\omega_{a_1a_2}p+a_1p\omega_{a_1p}+a_2p\omega_{a_2p}+a_1a_2p\omega_{a_1p}\omega_{a_2p}}, \tag{S27}$$

where $N_{\rm NS}$ is the number of non-specific binding sites; P is the number of RNAPs; A_1 is the number of the first activator; A_2 is the number of the second activator; $\Delta\varepsilon_{\rm pd}$ is the binding energy of the RNAP; $\Delta\varepsilon_{\rm a_1d}$ is the binding energy of the first activator; $\Delta\varepsilon_{\rm a_2d}$ is the binding energy of the second activator; $\omega_{\rm a_1p}$ is the interaction energy between the first activator and the RNAP; $\omega_{\rm a_2p}$ is the interaction energy between the second activator and the RNAP. On the other hand, for a double activation promoter with AND logic, we need to consider the interaction energy between the two activators, $\omega_{\rm a_1a_2}$. In this case, the probability of RNAP being bound is given by

$$p_{\rm bound} = \frac{p+a_1p\omega_{a_1p}+a_2p\omega_{a_2p}+a_1a_2p\omega_{a_1p}\omega_{a_2p}\omega_{a_1a_2}}{1+a_1+a_2+p+a_1a_2\omega_{a_1a_2}p+a_1p\omega_{a_1p}+a_2p\omega_{a_2p}+a_1a_2p\omega_{a_1p}\omega_{a_2p}\omega_{a_1a_2}}, \tag{S28}$$

where $\omega_{\rm a_1a_2}$ is the interaction energy between the two activators.

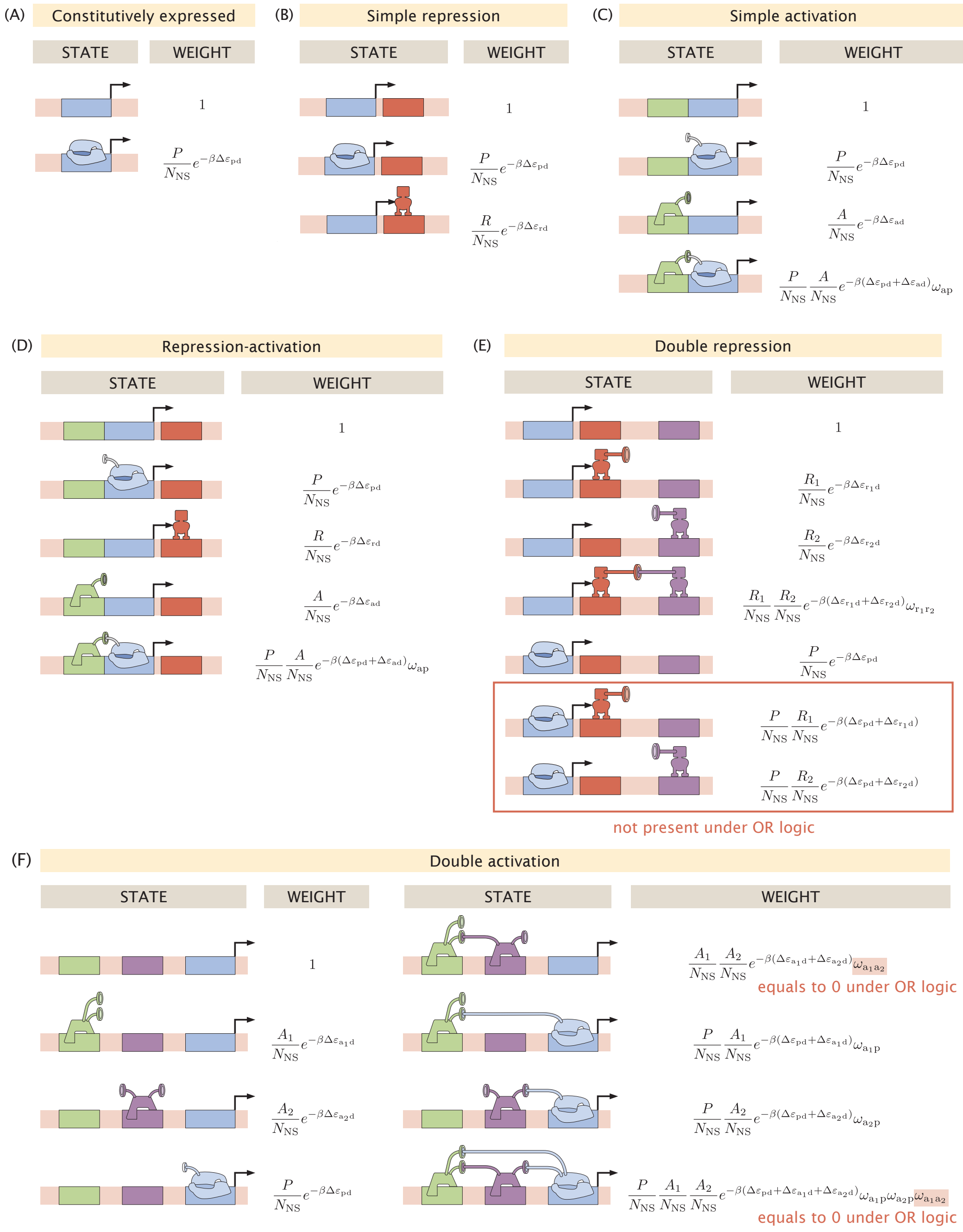

Fig S2. States-and-weights models for common regulatory architectures. In all the diagrams, P represents the number of RNAP; R represents the number of repressors; A represents the number of activators; N_{NS} represents the number of non-specific binding sites; $\Delta\varepsilon_{\mathrm{pd}}$ represents the binding energy of the RNAP; $\Delta\varepsilon_{\mathrm{rd}}$ represents the binding energy of the repressor; $\Delta\varepsilon_{\mathrm{ad}}$ represents the binding energy of the activator; $\omega_{\mathrm{ab}} = e^{-\beta\varepsilon_{\mathrm{int}}}$ represents the interaction energy between a and b. (A) States-and-weights model for a promoter that is constitutively expressed. (B) States-and-weights model for a promoter under the simple repression regulatory architecture. (C) States-and-weights model for a promoter under the simple activation regulatory architecture. (D) States-and-weights model for a promoter under the repression-activation regulatory architecture. (E) States-and-weights model for a promoter under the double repression regulatory architecture. Note that the last two states are only present under AND logic and are not present under OR logic. (F) States-and-weights model for a promoter under the double activation regulatory architecture. Note that the interaction energy between the two activators, $\omega_{\mathrm{a_1a_2}}$, is equal to 0 under OR logic.

SI references

1. Bintu L, Buchler NE, Garcia HG, Gerland U, Hwa T, Kondev J, and Phillips R. Transcriptional regulation by the numbers: models. Curr. Opin. Genet. Dev. 2005 Apr; 15:116–24
