# Supplementary material for: Deciphering regulatory architectures of bacterial promoters from synthetic expression patterns
Source: PLoS Comput Biol. 2024 Dec 26;20(12):e1012697. doi: 10.1371/journal.pcbi.1012697 (PMC11709304; doi:10.1371/journal.pcbi.1012697)
Supplement: S3 Appendix — (PDF) [file pcbi.1012697.s003.pdf]

S3 Appendix Producing synthetic datasets from first principles

S3.1 Definition of probability distributions in the calculation of mutual information

In order to build an information footprint from data, we need to calculate the mutual information between expression levels and the base identity at each position in the sequence, which is defined as

$$I_i = \sum_b \sum_\mu \Pr{}_i(b, \mu) \log_2 \left(\frac{\Pr{}_i(b, \mu)}{\Pr{}_i(b) \Pr(\mu)} \right), \tag{S29}$$

where b represents base identity and μ represents expression levels.

As shown in Fig S3(A) and S3(C), we find that binding sites have a higher signal-to-noise ratio in information footprints when the coarse grained approach is taken

$$b = \begin{cases} 0, & \text{if the base is mutated,} \\ 1, & \text{if the base is wild type.} \end{cases} \tag{S30}$$

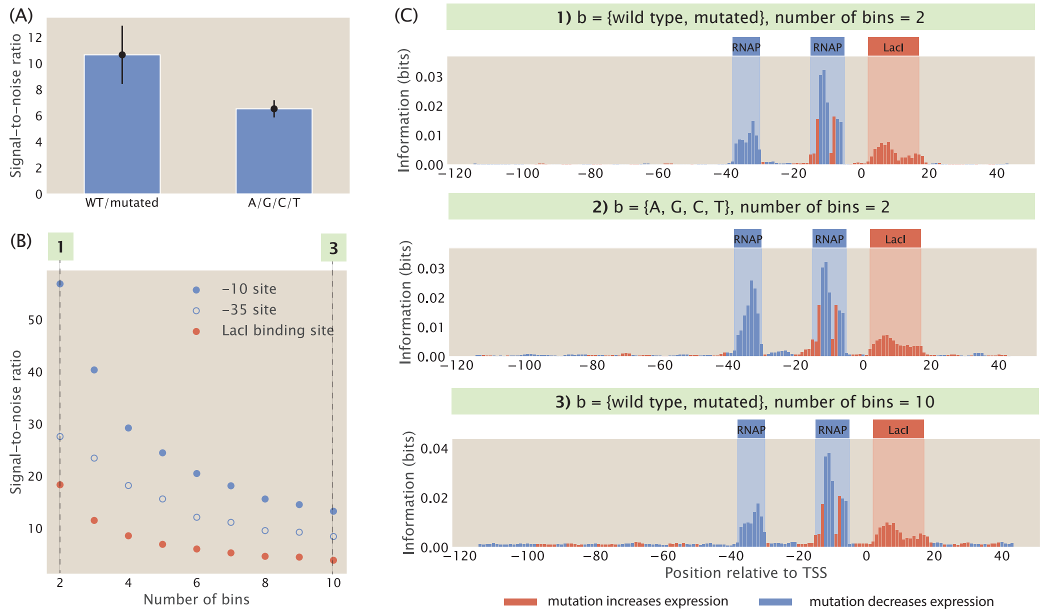

Fig S3. Definition of probability distributions in the calculation of mutual information. (A) Using a probability distribution of the four bases leads to a reduced signal-to-noise ratio in the information footprint. The heights of the bars are the average signal-to-noise ratios calculated from the information footprints of 20 synthetic datasets with the simple repression architecture. (B) Signal-to-noise ratio decreases when the number of bins increases. Each data point is the mean of average mutual information across 20 synthetic datasets with the corresponding number of bins. The numbered labels correspond to footprints in (C). (C) Choosing different probability distributions to calculate the information footprint for a synthetic dataset with the simple repression genetic architecture. The top footprint uses a probability distribution of wild-type and mutated bases and uses 2 bins to calculate the probability distribution for expression levels. The middle footprint uses a probability distribution of the four bases (A, G, C, T) and uses 2 bins to calculate the probability distribution for expression levels. The bottom footprint uses a probability distribution of wild-type and mutated bases and uses 10 bins to calculate the probability distribution for expression levels.

On the other hand, to obtain a distribution of expression levels, sequencing counts are binned into N bins, where the bins are chosen such there is an equal number of sequences in each bin. Again, we observe the highest signal-to-noise ratio for $N = 2$, and see a continuous decrease with increasing number of bins. As

shown in Fig S3(B) and S3(C), we observe that signal-to-noise ratio is higher when fewer bins are used to partition expression levels.

These observations may be explained by the fact that increasing the number of states or bins increases the level of noise. When there are more states or more bins, fewer sequences will be present in each bin. This amplifies the hitch-hiking effects discussed in Sec 1.3, leading to a higher level of noise. In addition, since the boundaries between the bins are artificially set, a sequence may be randomly grouped into the N-th bin rather than the adjacent (N-1)-th and (N+1)-th bins simply because the boundaries are set a particular level. This randomness occurs in both the marginal probability distribution for μ and the joint probability distribution, resulting in noise that is increased when more bins are added.

S3.2 Analytical calculation of an information footprint

To better understand how mutations in the binding sites create signals in the information footprint, we derive the information footprint analytically for a constitutively expressed gene, i.e. the promoter of the gene only has a binding site for RNAP and is not bound by any transcription factors.

Consider a promoter region where the RNAP binding site is l base pairs long and the probability of mutation at each site is θ. Furthermore, the binding energy of RNAP to the wild-type sequence is denoted by $\Delta\varepsilon$ and we assume that at each position within the binding site, a mutation comes with cost $\Delta\Delta\varepsilon$ to the binding energy. Therefore, if there are m mutations in the binding site, the total binding energy between RNAP and the mutant binding site is $\Delta\varepsilon + m\Delta\Delta\varepsilon$.

In a sufficiently large data set, the ratio of sequences with mutation at position i is given by

$$\Pr{}_i(b) = \begin{cases} 1-\theta, & \text{if } b = 0 \\ \theta, & \text{if } b = 1. \end{cases} \tag{S31}$$

Next, we determine $\Pr(\mu)$. As before, we define $\Pr(\mu)$ as the probability that a given sequence leads to high expression levels or low expression levels. To predict expression levels, we begin by calculating p_{bound} for each promoter variant. Since the gene is constitutively expressed, the probability of RNAP binding is given by

$$p_{\text{bound}} = \frac{\frac{P}{N_{\text{NS}}} e^{-\beta(\Delta\varepsilon + m\Delta\Delta\varepsilon)}}{1 + \frac{P}{N_{\text{NS}}} e^{-\beta(\Delta\varepsilon + m\Delta\Delta\varepsilon)}}. \tag{S32}$$

As derived in Eq 5 in the main text, the steady state copy number of mRNAs is proportional to the probability of the RNAP bound state. Therefore, expression level is only dependent on the number of mutations in the RNAP binding site.

The probability distribution for the number of mutations in the RNAP binding site can be expressed using the binomial distribution, where the probability of k mutations in the binding site is given by

$$\Pr(m = k; l, \theta) = \binom{l}{k} \theta^k (1-\theta)^{l-k}. \tag{S33}$$

As illustrated in Fig S4, since expression levels are solely determined by the number of mutations in the binding site, and sequences are binned by expression levels to obtain $P(\mu)$, there is a threshold number of mutations, m^*, where sequences with m^* or more than m^* mutations fall into the lower expression bin. Hence, $P(\mu)$ is given by

$$\Pr(\mu) = \begin{cases} \Pr(m \geq m^*; l, \theta) = \sum_{k=m^*}^{l} \binom{l}{k} \theta^k (1-\theta)^{l-k}, & \text{if } \mu = 0 \\ \Pr(m < m^*; l, \theta) = 1 - \Pr(m \geq m^*; l, \theta), & \text{if } \mu = 1. \end{cases} \tag{S34}$$

Finally, we determine the expression for $\Pr{}_i(b, \mu)$. To do this, we consider two cases, one where the position i is outside of the RNAP binding site and one where the position i is within the RNAP binding site.

If i is not in the RNAP binding site $\mathcal{B}$, then a mutation would have no effect on the expression levels, therefore

$$\Pr_{i\notin\mathcal{B}}(b,\mu) = \begin{cases} (1-\theta)\cdot\Pr(m\geq m^*;l,\theta), & \text{if } b=0 \text{ and } \mu=0 \\ (1-\theta)\cdot\Pr(m< m^*;l,\theta), & \text{if } b=0 \text{ and } \mu=1 \\ \theta\cdot\Pr(m\geq m^*;l,\theta), & \text{if } b=1 \text{ and } \mu=0 \\ \theta\cdot\Pr(m< m^*;l,\theta), & \text{if } b=1 \text{ and } \mu=1. \end{cases} \tag{S35}$$

Having derived all the marginal probability distributions and the joint probability distributions, we can then write down mutual information at a non-binding site and at a binding site. If position i is outside the RNAP binding site, then

$$\begin{aligned} I_i =&(1-\theta)\cdot\Pr(m\geq m^*;l,\theta)\log_2\frac{(1-\theta)\cdot\Pr(m\geq m^*;l,\theta)}{(1-\theta)\cdot\Pr(m\geq m^*;l,\theta)} \\ &+(1-\theta)\cdot\Pr(m< m^*;l,\theta)\log_2\frac{(1-\theta)\cdot\Pr(m< m^*;l,\theta)}{(1-\theta)\cdot\Pr(m< m^*;l,\theta)} \\ &+\theta\cdot\Pr(m\geq m^*;l,\theta)\log_2\frac{\theta\cdot\Pr(m\geq m^*;l,\theta)}{\theta\cdot\Pr(m\geq m^*;l,\theta)} \\ &+\theta\cdot\Pr(m< m^*;l,\theta)\log_2\frac{\theta\cdot\Pr(m< m^*;l,\theta)}{\theta\cdot\Pr(m< m^*;l,\theta)}. \end{aligned} \tag{S36}$$

We can see that $I_i = 0$ since the fractions within the logarithms all cancel out to be 1. This is because the joint probability $\Pr_i(b,\mu)$ for bases outside the binding site is simply given by the product of the marginal distributions,

$$\Pr_{i\notin\mathcal{B}}(b,\mu) = \Pr_{i\notin\mathcal{B}}(\mu)\Pr_{i\notin\mathcal{B}}(b). \tag{S37}$$

If the position i is in the RNAP binding site, the calculation for $\Pr_i(b,\mu)$ is more complex. As illustrated in Fig S4, for a position with wild-type base identity, the sequence will have low expression levels if there are more than m^* mutations in the remaining $l-1$ bases in the RNAP binding site, and high expression levels if there are less than m^* mutations in the remaining $l-1$ bases in the RNAP binding site. On the other hand, for a position that is mutated, the sequence will have low expression levels if there are more than m^*-1 mutations in the remaining $l-1$ bases, and high expression levels if there are less than m^*-1 mutations in the remaining $l-1$ bases. Taken together, we can write down the joint probability distribution as

$$\Pr_{i\in\mathcal{B}}(b,\mu) = \begin{cases} (1-\theta)\cdot\Pr(m\geq m^*;l-1,\theta), & \text{if } b=0 \text{ and } \mu=0 \\ (1-\theta)\cdot\Pr(m< m^*;l-1,\theta), & \text{if } b=0 \text{ and } \mu=1 \\ \theta\cdot\Pr(m\geq m^*-1;l-1,\theta), & \text{if } b=1 \text{ and } \mu=0 \\ \theta\cdot\Pr(m< m^*-1;l-1,\theta), & \text{if } b=1 \text{ and } \mu=1. \end{cases} \tag{S38}$$

In this case, the joint distribution does not factor into the marginal distributions,

$$\Pr_{i\in\mathcal{B}}(b,\mu) \neq \Pr_{i\in\mathcal{B}}(\mu)\Pr_{i\in\mathcal{B}}(b), \tag{S39}$$

and therefore, mutual information has to be larger than zero, $I_i > 0$, clearly distinguishing positions that are

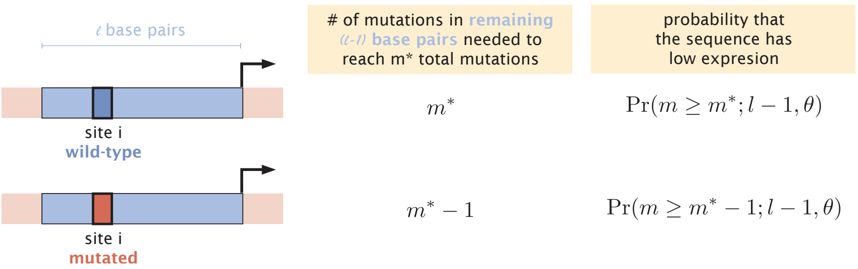

Fig S4. Calculating number of mutations needed to reach the threshold between low expression and high expression bins. The joint probability distribution at site i is a product of the probability that site i is mutated or wild-type and the probability that the sequence has high or low expression level. Since the expression of the sequence depends on the presence of a mutation at site i, we need to consider two different cases in order to calculate the probability of expression. In the case where site i has wild-type base identity, there need to be m^* mutations outside of site i in the RNAP binding site for the sequence to reach the threshold m^*. Therefore, the probability that the sequence has low expression is $\Pr(m \geq m^*; l-1, \theta)$. On the other hand, in the case where site i is mutated, since one mutation is known to exist, there only need to be $m^* - 1$ mutations outside of site i in the RNAP binding site for the sequence to reach the threshold. In this case, the probability that the sequence has low expression is $\Pr(m \geq m^* - 1; l-1, \theta)$.

within the binding site from positions outside. Specifically,

$$\begin{aligned}
I_i =& (1-\theta) \cdot \Pr(m \geq m^*; l-1, \theta) \log_2 \frac{(1-\theta) \cdot \Pr(m \geq m^*; l-1, \theta)}{(1-\theta) \cdot \Pr(m \geq m^*; l, \theta)} \\
&+ (1-\theta) \cdot \Pr(m < m^*; l-1, \theta) \log_2 \frac{(1-\theta) \cdot \Pr(m < m^*; l-1, \theta)}{(1-\theta) \cdot \Pr(m < m^*; l, \theta)} \\
&+ \theta \cdot \Pr(m \geq m^* - 1; l-1, \theta) \log_2 \frac{\theta \cdot \Pr(m \geq m^* - 1; l-1, \theta)}{\theta \cdot \Pr(m \geq m^*; l, \theta)} \\
&+ \theta \cdot \Pr(m < m^* - 1; l-1, \theta) \log_2 \frac{\theta \cdot \Pr(m < m^* - 1; l-1, \theta)}{\theta \cdot \Pr(m < m^*; l, \theta)} \\
=& (1-\theta) \cdot \Pr(m \geq m^*; l-1, \theta) \log_2 \frac{\Pr(m \geq m^*; l-1, \theta)}{\Pr(m \geq m^*; l, \theta)} \\
&+ (1-\theta) \cdot \Pr(m < m^*; l-1, \theta) \log_2 \frac{\Pr(m < m^*; l-1, \theta)}{\Pr(m < m^*; l, \theta)} \\
&+ \theta \cdot \Pr(m \geq m^* - 1; l-1, \theta) \log_2 \frac{\Pr(m \geq m^* - 1; l-1, \theta)}{\Pr(m \geq m^*; l, \theta)} \\
&+ \theta \cdot \Pr(m < m^* - 1; l-1, \theta) \log_2 \frac{\Pr(m < m^* - 1; l-1, \theta)}{\Pr(m < m^*; l, \theta)}.
\end{aligned} \tag{S40}$$

S3.3 Comparison between synthetic and experimental information footprints

To show that we can produce synthetic information footprints that are comparable to the information footprints obtained from experimental MPRA data, here we present a side-by-side comparison of synthetic and experimental footprints for each of the six most common regulatory architectures. The experimental footprints are obtained using the Reg-Seq pipeline by Ireland et al. [1]. Note that we do not have the energy matrices in $k_B T$ units for the transcription factors in these footprints, nor do we often know the copy numbers of the related transcription factors. Therefore, we could not exactly replicate the signals observed in the experimental footprints. However, the signals for RNAP appear to be similar and the footprints exhibit a similar structure as the synthetic footprints.

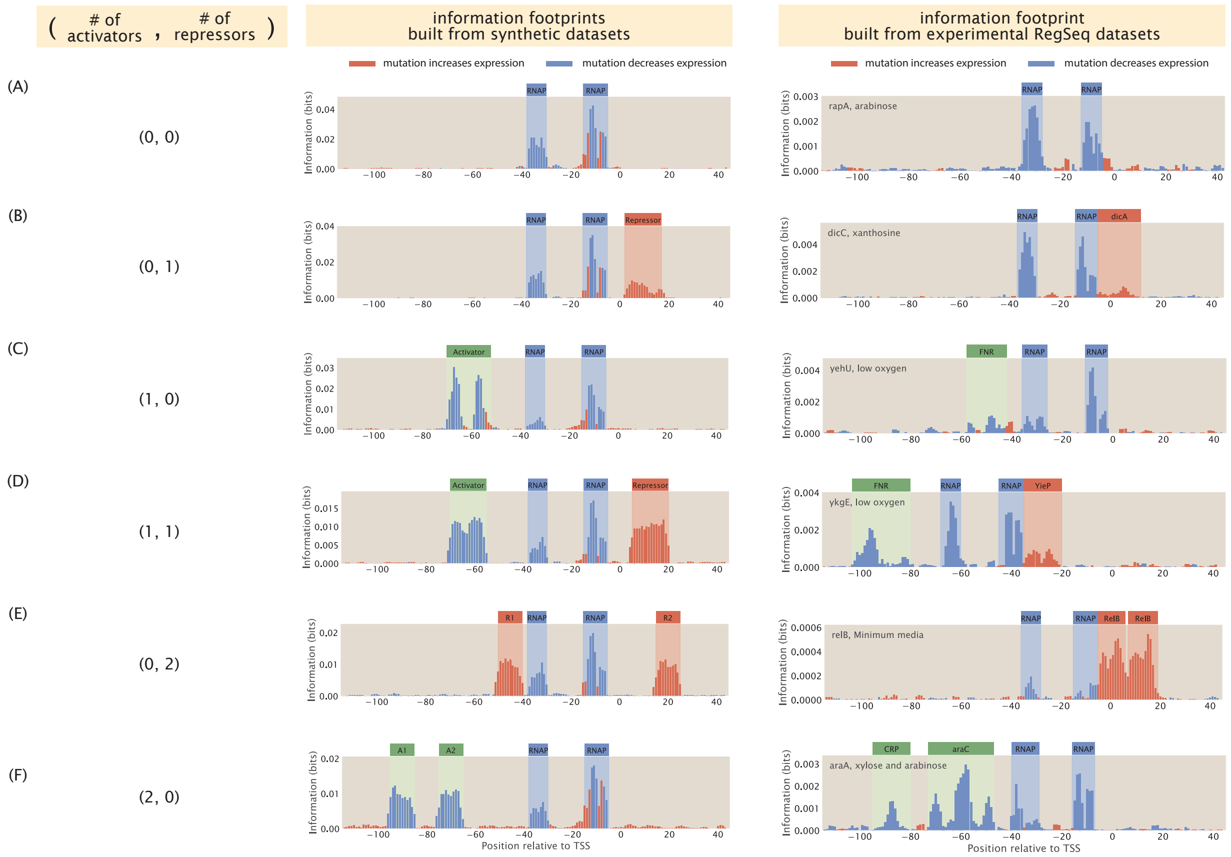

Fig S5. Comparison of information footprints built from synthetic datasets and information footprints built from experimental datasets Comparison of synthetic footprints and experimental footprints for the six most common regulatory architectures. The experimental footprints are built from datasets obtained through Reg-Seq, an MPRA developed by Ireland et al. [1].

SI references

1. Ireland WT, Beeler SM, Flores-Bautista E, McCarty NS, Röschinger T, Belliveau NM, Sweredoski MJ, Moradian A, Kinney JB, and Phillips R. Deciphering the regulatory genome of *Escherichia coli*, one hundred promoters at a time. eLife 2020 Sep; 9:e55308
