# Supplementary material for: Deciphering regulatory architectures of bacterial promoters from synthetic expression patterns
Source: PLoS Comput Biol. 2024 Dec 26;20(12):e1012697. doi: 10.1371/journal.pcbi.1012697 (PMC11709304; doi:10.1371/journal.pcbi.1012697)
Supplement: S4 Appendix — (PDF) [file pcbi.1012697.s004.pdf]

S4 Appendix Effects of mutation rate on information footprints

S4.1 Recovering binding site signal under extreme mutation rates

As we have shown in Sec 1.2, when the rate of mutation in the mutant library is low, we lose the signal at the RNAP binding site. We hypothesize that this is because RNAP binds weakly at the promoter. We generated a synthetic dataset that has low mutation rate but stronger binding energy at the RNAP binding site. As shown in Fig S6(A), the information footprint built from this dataset has a much higher level of mutual information at the RNAP binding site compared to the information footprint built from a dataset with the same mutation rate but weak RNAP binding energy, which supports our hypothesis.

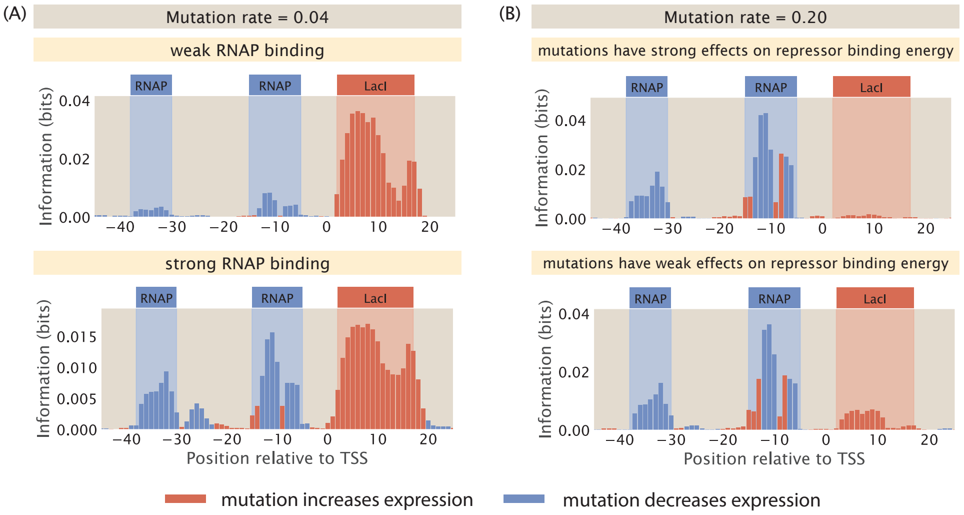

Fig S6. Recovering signals from information footprints under extreme mutation rates. (A) We generated two synthetic datasets with a mutation rate of 0.04 in the mutant library. In the first dataset, we set the RNAP binding energy $\Delta\varepsilon_{pd}$ to be $-5\ k_BT$, which is typical of RNAP binding at the wild type -10 and -35 binding sites. In the footprint produced from this dataset, there is low mutual information at the RNAP binding site due to the low mutation rate. On the other hand, in the second dataset, we increased $\Delta\varepsilon_{pd}$ to $-12\ k_BT$. This allows us to recover the signal at the RNAP binding site. (B) We generated two synthetic datasets with a mutation rate of 0.20. In the first dataset, we used the experimentally measured energy matrix for LacI at the O1 operator shown in Fig 3(B), where the average effect of mutations on binding energy is 2.24 k_BT. In the footprint produced from this dataset, there is low mutual information at the repressor binding site due to the high mutation rate. In the second dataset, we reduce the average effect of mutations five-fold and are able to recover the signal at the repressor binding site.

We also showed that when the rate of mutation in the mutant library is high, there is low mutual information at the repressor binding site. Our hypothesis is that this is caused by the large effects of mutations on the repressor binding energy. We generated a synthetic dataset with high mutation rate while reducing the effect of mutation on binding energy by five fold. As shown in Fig S6(B), this allows us to recover the signal at the repressor binding site, which is also in line with our hypothesis.

S4.2 Calculating the optimal mutation rate under different sets of parameters

An essential part of designing libraries for MPRAs is determining the rate at which bases in the sequences are mutated. In Sort-Seq [1] and Reg-Seq [2], the mutation rate for promoter sequences is chosen as 0.1 per base. In Sec 1.2, we have calculated that this is on par with the optimal mutation rate for a promoter regulated by one transcription factor, given a specific set of parameters for copy numbers and binding energies of the transcription factor and RNAP. Here, we explore how the result for the optimal mutation rate depends on the specific choice of parameters.

In Eq 16 in the main text, we have defined the optimal mutation rate as the rate where the Boltzmann weights of RNAP binding and transcription factor binding are equal. First, we explore how this mutation rate depends on the binding energy of RNAP $\Delta\varepsilon_{\mathrm{pd}}$, the binding energy of the transcription factor $\Delta\varepsilon_{\mathrm{rd}}$, the copy number of RNAP P, and the copy number of the transcription factor R. For each set of parameters, we can solve Eq 16 numerically for the mutation rate that gives us $\kappa = 1$. In Fig S7, the optimal mutation rate is computed numerically when two of the parameters are varied while the others are kept constant. Increasing the binding energy or copy number of the transcription factor increases the mutation rate, while increasing the binding energy and copy number of RNAP decreases the mutation rate. Changing the binding energy can have drastic effects on the mutation rate, in contrast to changes in copy numbers. This can be explained by the fact that the binding energies contributing exponentially to κ, while the copy numbers come into play as linear factors. For cases where the transcription factor bound state becomes very unlikely, e.g. in the cases of very weak binding of the transcription factor or very strong binding of the RNAP, there is no optimal mutation rate that can be found given the criteria in Eq 16. These regions can be found in Fig S7(B)-(D) as grey regions.

In addition, we explore how the optimal mutation rate depends on the length of the binding site and the extent to which each mutation changes the total energy. The effect of each mutation depends on the information content of the energy matrix. For an energy matrix with high information content, each mutation would lead to a large change in binding energy. On the other hand, for an energy matrix with low information content, each mutation would only lead to a small change in binding energy. In Fig S8, we vary each pair of parameters while keeping the other parameters constant. We see that for the vast majority of parameter values within the physiologically relevant range, the optimal mutation rate falls under 0.5.

Binding sites and transcription factors come with widely different values for the parameters we have tested, e.g., the copy number of the activator CRP can be as high as about 500, while the copy number for an essential transcription factor DicA can be as low as 10 as measured in mass spectrometry experiments [3]. The binding energy for LacI varies on the order of $6\,k_{\mathrm{B}}T$ ($-15.7\,k_{\mathrm{B}}T$ for the O1 operator and $-9.3\,k_{\mathrm{B}}T$ for the O3 operator) [4]. Hence, it can be beneficial to create a library that contains sequences with different mutation rates in order to detect binding sites with these different parameters.

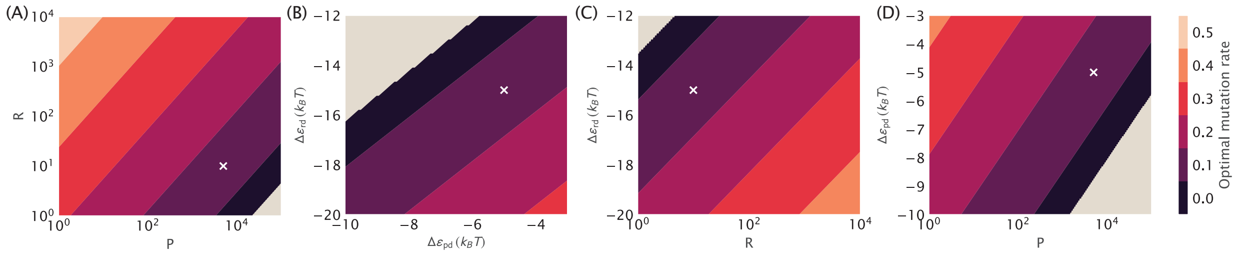

Fig S7. Optimal mutation rate as a function of transcription factor copy numbers and wild-type binding energies. Numerical solutions for the mutation rate calculated by finding the mutation rate that leads to equal Boltzmann weights between the RNAP bound state and the transcription factor bound state using Eq 16. The white crosses mark the standard set of parameters: $R = 10$, $P = 5000$, $\Delta\varepsilon_{\mathrm{rd}} = -15\,k_{\mathrm{B}}T$ and $\Delta\varepsilon_{\mathrm{pd}} = -15\,k_{\mathrm{B}}T$. Panels (A) to (D) vary 2 of the 4 parameters, while the other two stay constant. Gray color indicates regimes where no mutation rate can be found that fulfills the criteria of $\kappa = 1$.

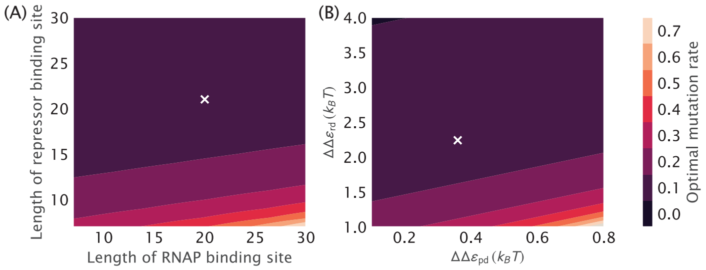

Fig S8. Optimal mutation rate as a function of the size of the binding sites and information content of energy matrices. Numerical solutions for the mutation rate calculated using Eq 16. (A) Varying the length of the RNAP and repressor binding sites. (B) Varying $\Delta\Delta\varepsilon_{\mathrm{pd}}$ and $\Delta\Delta\varepsilon_{\mathrm{rd}}$, which are the changes in total binding energy given a single mutation at the RNAP binding site and at the repressor binding site. The white crosses mark the standard set of parameters: $l_r = l_p = 20$, $\Delta\Delta\varepsilon_{\mathrm{pd}} = 0.36\,k_{\mathrm{B}}T$ and $\Delta\Delta\varepsilon_{\mathrm{rd}} = 2.24\,k_{\mathrm{B}}T$.

SI references

1. Kinney JB, Murugan A, Callan Jr CG, and Cox EC. Using deep sequencing to characterize the biophysical mechanism of a transcriptional regulatory sequence. Proc. Natl. Acad. Sci. U. S. A. 2010 May; 107:9158–63
2. Ireland WT, Beeler SM, Flores-Bautista E, McCarty NS, Röschinger T, Belliveau NM, Sweredoski MJ, Moradian A, Kinney JB, and Phillips R. Deciphering the regulatory genome of *Escherichia coli*, one hundred promoters at a time. eLife 2020 Sep; 9:e55308
3. Schmidt A, Kochanowski K, Vedelaar S, Ahrné E, Volkmer B, Callipo L, Knoops K, Bauer M, Aebersold R, and Heinemann M. The quantitative and condition-dependent *Escherichia coli* proteome. Nat. Biotechnol. 2016 Jan; 34:104–10
4. Garcia HG and Phillips R. Quantitative dissection of the simple repression input-output function. Proc. Natl. Acad. Sci. U. S. A. 2011 Jul; 108:12173–8
