# Supplementary material for: Deciphering regulatory architectures of bacterial promoters from synthetic expression patterns
Source: PLoS Comput Biol. 2024 Dec 26;20(12):e1012697. doi: 10.1371/journal.pcbi.1012697 (PMC11709304; doi:10.1371/journal.pcbi.1012697)
Supplement: S5 Appendix — (PDF) [file pcbi.1012697.s005.pdf]

S5 Appendix Choosing an appropriate library size in MPRAs

S5.1 Analytical demonstration of hitch-hiking effects

To demonstrate the hitch-hiking effect analytically, we consider a hypothetical promoter that is constitutively transcribed and only two base pairs long, as illustrated in Fig S9(A). Without loss of generality, we assume that there are only two letters in the nucleotide alphabet, X and Y. Therefore, a complete and unbiased library contains four sequences: XX, YX, XY, and YY. We designate that $\varepsilon_X < \varepsilon_Y$, i.e. the RNAP is strongly bound at the binding site when the base identity is X and weakly bound when the base identity is Y. We also assume that there is active transcription only when RNAP is bound to the second site. Under these assumptions, there are high expression levels when the promoter sequence is XX or YX and low expression levels when the promoter sequence is XY or YY.

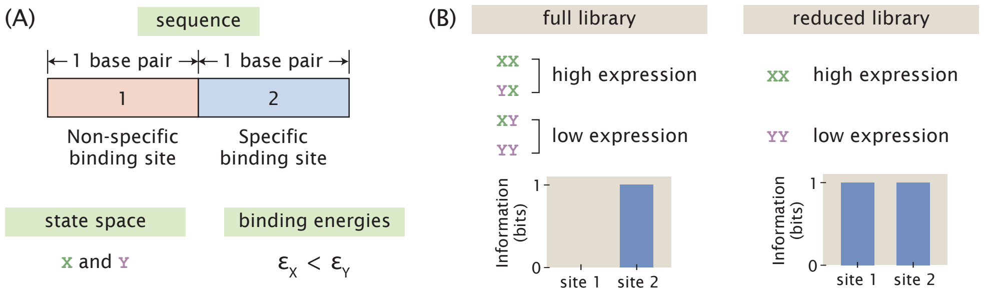

Fig S9. Hitch-hiking effect in the hypothetical minimal promoter. (A) Set-up of the hypothetical minimal promoter. The specific and non-specific sites of the minimal promoter are each 1 base pair long. There are two possible bases at each binding site, X and Y. Strong binding occurs when the base is X, whereas weak binding occurs when the base is Y. (B) Effect of library size on the information footprint of the minimal promoter. A full mutant library consists of all four possible sequences and leads to a footprint with no signal outside of the specific binding sites. On the other hand, a reduced mutant library with only two sequences creates noise outside of the specific binding sites. In this case, the noise at the non-binding site has the same magnitude as the signal at the specific binding site.

We first consider a mutant library with full diversity and no bias, i.e. the four possible sequences, XX, YX, XY, and YY, are each present in the library exactly once. The marginal probability distribution for expression levels is

$$\Pr(\mu) = \begin{cases} 0.5, & \text{if } \mu = 0 \\ 0.5, & \text{if } \mu = 1. \end{cases} \tag{S41}$$

The marginal probability distributions of base identity at the two sites are

$$\Pr{}_1(b) = \Pr{}_2(b) = \begin{cases} 0.5, & \text{if } b = \text{X} \\ 0.5, & \text{if } b = \text{Y}. \end{cases} \tag{S42}$$

The joint probability distribution at the first site is

$$\Pr{}_1(\mu, b) = \begin{cases} 0.25, & \text{if } \mu = 0 \text{ and } b = \text{X} \\ 0.25, & \text{if } \mu = 0 \text{ and } b = \text{Y} \\ 0.25, & \text{if } \mu = 1 \text{ and } b = \text{X} \\ 0.25, & \text{if } \mu = 1 \text{ and } b = \text{Y}. \end{cases} \tag{S43}$$

On the other hand, the joint probability distribution at the second site is

$$\Pr{}_2(\mu, b) = \begin{cases} 0, & \text{if } \mu = 0 \text{ and } b = \text{X} \\ 0.5, & \text{if } \mu = 0 \text{ and } b = \text{Y} \\ 0.5, & \text{if } \mu = 1 \text{ and } b = \text{X} \\ 0, & \text{if } \mu = 1 \text{ and } b = \text{Y}. \end{cases} \tag{S44}$$

We can calculate the mutual information at each site according to Eq 6,

$$I_1 = 4\left(\frac{1}{4}\log_2\left(\frac{1/4}{1/2 \cdot 1/2}\right)\right) = 0 \tag{S45}$$

$$I_2 = 2\left(\frac{1}{2}\log_2\left(\frac{1/2}{1/2 \cdot 1/2}\right)\right) = 1. \tag{S46}$$

Therefore, when the library has the maximum size, there is perfect signal at the specific binding site and no signal outside of the specific binding site, as shown in Fig S9(B).

On the other hand, consider a reduced library that only consists of XX and YY. According to the assumptions stated above, XX has high expression and YY has low expression. In this case, there is an apparent correlation between the base identity at the non-binding site and expression levels, where a base identity of X at the non-binding site appears to lead to high expression levels and a base identity of Y at the non-binding site appears to lead to low expression levels. To demonstrate this analytically, we again write down the relevant probability distributions required for calculating mutual information. The marginal probability distributions for expression levels and base identity are the same as the case where we have a full library. However, the joint probability distributions at both of the two sites become

$$\Pr{}_1(\mu, b) = \Pr{}_2(\mu, b) = \begin{cases} 0, & \text{if } \mu = 0 \text{ and } b = \text{X} \\ 0.5, & \text{if } \mu = 0 \text{ and } b = \text{Y} \\ 0.5, & \text{if } \mu = 1 \text{ and } b = \text{X} \\ 0, & \text{if } \mu = 1 \text{ and } b = \text{Y}. \end{cases} \tag{S47}$$

This means that for both the non-binding site and the specific binding site, the mutual information is

$$I_1 = I_2 = 2\left(\frac{1}{2}\log_2\left(\frac{1/2}{1/2 \cdot 1/2}\right)\right) = 1. \tag{S48}$$

As shown in Fig S9(B), this creates an artificial signal, or noise, outside of the specific binding sites that cannot be distinguished from the signal at the specific binding site.

S5.2 Cost of library synthesis as a function of oligo pool size

Using Reg-Seq, our group has successfully deciphered the regulatory architecture of several hundred *E. coli* promoters [1, 2, 3]. The next goal is to scale up Reg-Seq and dissect the regulatory architectures of every gene in an entire genome. In *E. coli*, which is the model organism that we will start with to test our high-throughput pipeline, there are on the order of a few thousand promoters [4]. This means that we need a Reg-Seq pipeline that can be used to dissect 10^3 promoters at a time.

A major bottleneck to scale-up Reg-Seq is the financial cost associated with synthesizing large mutant libraries. In previous iterations of Reg-Seq, we used a mutant library of 1,500 sequence variants for each promoter, where each sequence variant contains scattered random mutations [1]. As demonstrated in Fig 6, using the scattered-mutation library, we cannot cut down the library size to a lower order of magnitude without drastically reducing signal-to-noise ratio. However, if we continue to use a library with 10^3 sequences for every promoter in *E. coli*, the total number of sequence variants would exceed 10^6. In addition, the cost of synthesis also depends on the length of the oligos. In the original Reg-Seq library, each oligo contains a 160 base pair promoter variant, two 20 base pair primer binding sites at each end of the promoter variant, and two 6 base pair restriction sites that are needed for adding the GFP reporter gene and for inserting the

library into plasmids. Taken together, the total length of the oligo is $160 + 2 \times 20 + 2 \times 6 = 212$ base pair long. In our lab, mutant libraries are synthesized in the form of oligo pools. Fig S10 shows the cost of synthesizing different sizes of oligo pools with different oligo lengths. Based on Fig S10, we see that the cost of synthesizing an oligo pool with 10^6 oligos that are 212 base pair long is around 60 thousand dollars. To make the venture of deciphering the regulatory architectures of whole genomes financially viable, an alternative library design may be required where fewer sequences are needed to maintain the level of information that we can extract from sequencing data.

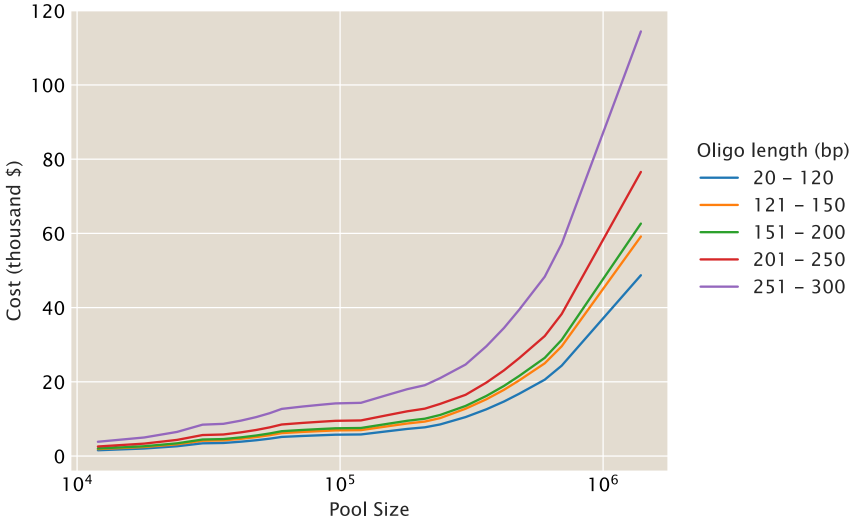

Fig S10. Cost of synthesizing oligo pools of different sizes. The numbers are obtained from the Twist Bioscience oligo pool cost sheet. Note that these costs take into account academic discounts that are offered for the synthesis of large pools.

SI references

1. Ireland WT, Beeler SM, Flores-Bautista E, McCarty NS, Röschinger T, Belliveau NM, Sweredoski MJ, Moradian A, Kinney JB, and Phillips R. Deciphering the regulatory genome of *Escherichia coli*, one hundred promoters at a time. eLife 2020 Sep; 9:e55308
2. Barnes SL, Belliveau NM, Ireland WT, Kinney JB, and Phillips R. Mapping DNA sequence to transcription factor binding energy in vivo. PLoS Comput. Biol. 2019 Feb; 15:e1006226
3. Belliveau NM, Barnes SL, Ireland WT, Jones DL, Sweredoski MJ, Moradian A, Hess S, Kinney JB, and Phillips R. Systematic approach for dissecting the molecular mechanisms of transcriptional regulation in bacteria. Proc. Natl. Acad. Sci. U. S. A. 2018 May; 115:E4796–E4805
4. Urtecho G, Tripp AD, Insigne KD, Kim H, and Kosuri S. Systematic Dissection of Sequence Elements Controlling σ70 Promoters Using a Genomically Encoded Multiplexed Reporter Assay in *Escherichia coli*. Biochemistry 2019 Mar; 58:1539–51
