# Supplementary material for: Deciphering regulatory architectures of bacterial promoters from synthetic expression patterns
Source: PLoS Comput Biol. 2024 Dec 26;20(12):e1012697. doi: 10.1371/journal.pcbi.1012697 (PMC11709304; doi:10.1371/journal.pcbi.1012697)
Supplement: S6 Appendix — (PDF) [file pcbi.1012697.s006.pdf]

S6 Appendix Changing transcription factor copy numbers under different regulatory logics

S6.1 Double repression promoter under XOR logic

In Sec 2.1, we examined how changing transcription factor copy numbers affect the footprint for a double repression promoter under AND and OR logic. As discussed by Buchler et al. [1] and de Ronde et al. [2], only a limited number of logic gates are attainable through parameter variations, and a thermodynamic model can be written down for each of the possible logic gates. This means that our thermodynamic-model-based computational pipeline can be easily adapted to consider all possible types of interactions between transcription factors.

As an example, let us consider exclusive-or (XOR) logic in a promoter regulated by two repressors, which is another important and interesting logic gate other than the AND and OR logics that we considered in Fig 10. As illustrated in Fig S11(A), under the XOR gate, gene expression is repressed when only one of the repressors is present at high concentrations, but not when both of the repressors are present at high concentrations. One possible mechanism by which this may occur is if the interaction between the two repressors is repulsive. As shown in Fig S11(B) and S11(C), when the copy number of the second repressor is kept constant at 25 and the copy number of the first repressor is increased from 0 to 50, the signal at the first repressor binding site increases and the signal at the second repressor binding site decreases. This behaviour is consistent with the definition of XOR logic gates and demonstrates that our computational pipeline can handle a diverse range of interaction regimes.

S6.2 Double activation promoter under AND and OR logics

A double activation promoter can also operate under AND or OR logic [1]. The states-and-weights diagram for a double activation promoter is shown in Fig S2(F). Under AND logic, the two activators can interact both with the RNAP and with each other. This cooperativity leads to a further increase in expression levels. In contrast, under OR logic, the activators independently interact with RNAP and there is no cooperativity between them. We build synthetic datasets for an AND-logic and an OR-logic double activation promoter. As shown in Fig S12(A) and S12(B), under AND logic, since cooperativity is at play, the signal at both A_1 and A_2 binding sites increases when A_1 is increased. On the other hand, under OR logic, the two activators act independently and there is competition between the signals at the two sites. When A_1 is increased, the signal at A_1 binding site correspondingly increases but the signal at A_2 binding site decreases.

SI references

1. Buchler NE, Gerland U, and Hwa T. On schemes of combinatorial transcription logic. Proc. Natl. Acad. Sci. U. S. A. 2003 Apr; 100:5136–41
2. Ronde W de, Rein ten Wolde P, and Mugler A. Protein logic: a statistical mechanical study of signal integration at the single-molecule level. Biophys. J. 2012 Sep; 103:1097–107

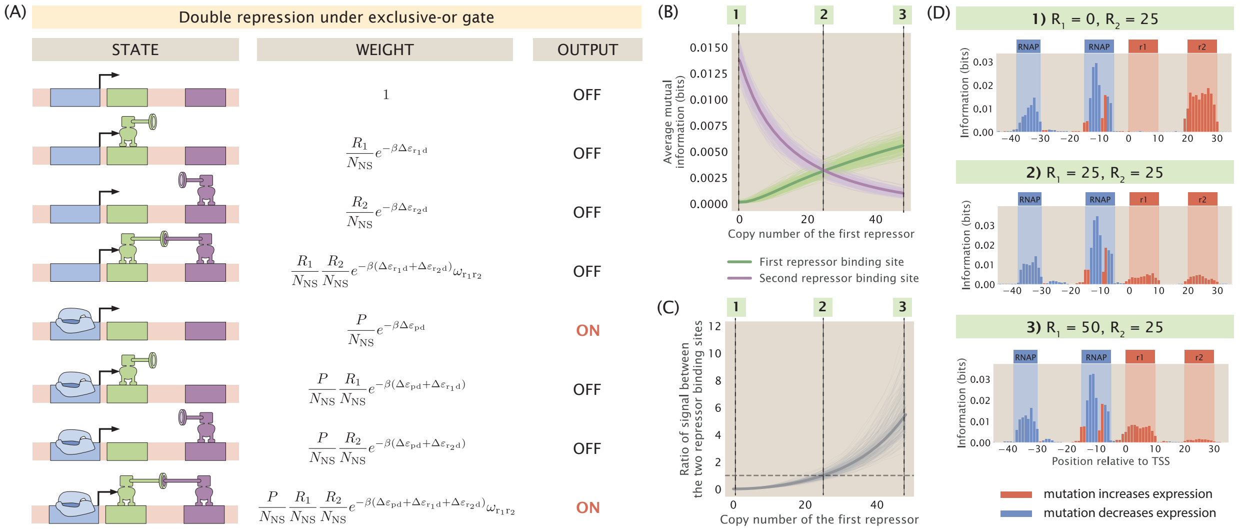

Fig S11. Changing repressor copy number for a double-repression promoter under XOR logic. (A) States-and-weights diagram of a promoter with the double repression regulatory architecture and under the exclusive-or (XOR) gate. (B) Changes in the average mutual information at the two repressor binding sites when the copy number of the first repressor is increased. For the energy matrices of the repressors, the interaction energy between the repressor and a site is set to $0\, k_BT$ if the site has the wild type base identity and set to $1\, k_BT$ if the site has the mutant base identity. To enforce the XOR logic, the interaction energy between the repressors is set to $5\, k_BT$. 200 synthetic datasets are simulated and the trajectory for each of the synthetic dataset is shown as an individual light green or light purple curve. The average trajectories across all 200 synthetic datasets are shown as the bolded green and purple curves. The three numbered labels correspond to the information footprints shown in (D). (C) Ratio of average mutual information between the two repressor binding sites when the copy number of the first repressor changes. The individual trajectories (plotted in light grey) and mean trajectory (plotted in dark grey) are from the same 200 synthetic datasets used in generate the plot in (B). As expected, the ratio is equal to 1 when the copy number of the first repressor is equal to the copy number of the second repressor. (D) Representative information footprints with three different combinations of repressor copy numbers.

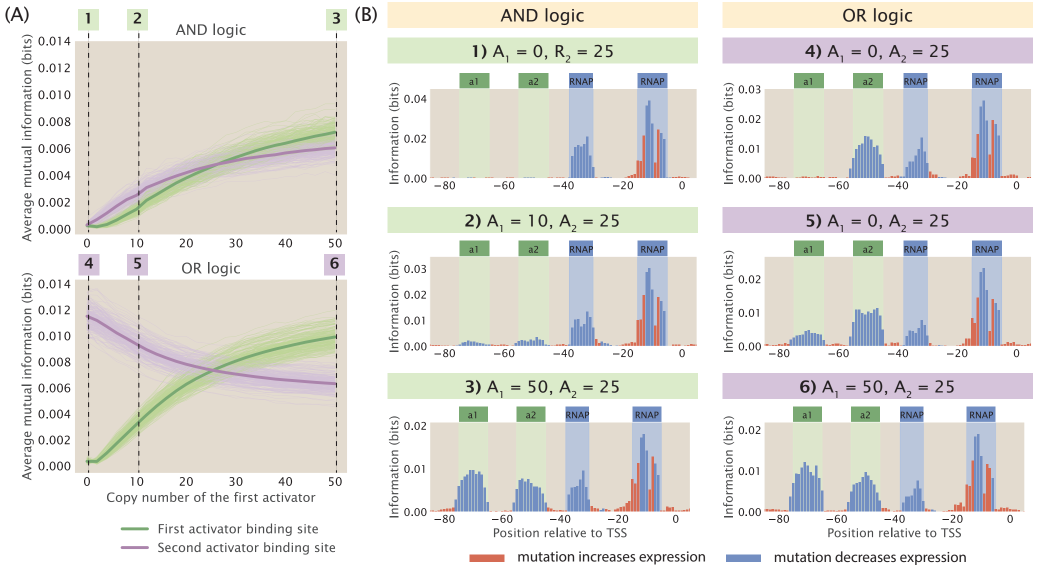

Fig S12. Changing the copy number of activators in a double activation promoter. (A) Changing the copy number of the first activator under AND logic and OR logic affects the signal at both activator binding sites. The energy matrices of the activators are randomly generated in the same way as the energy matrices of the repressors in Fig 8. For the promoter with AND logic, the interaction energies between the activators and between the activator and the RNAP are set to -4 k_BT. For the promoter with OR logic, the interaction energies between the activators and between the activator and the RNAP are set to -7 k_BT. The higher interaction energy for the OR logic promoter is to ensure that there are similar levels of signal at the activator binding sites compared to the AND logic promoter. 200 synthetic datasets are simulated and the trajectory for each of the synthetic dataset is shown as an individual light green or light purple curve. The average trajectories across all 200 synthetic datasets are shown as the bolded green and purple curves.
(B) Representative information footprints of a double repression promoter under AND and OR logic.
