# Supplementary material for: Deciphering regulatory architectures of bacterial promoters from synthetic expression patterns
Source: PLoS Comput Biol. 2024 Dec 26;20(12):e1012697. doi: 10.1371/journal.pcbi.1012697 (PMC11709304; doi:10.1371/journal.pcbi.1012697)
Supplement: S7 Appendix — (PDF) [file pcbi.1012697.s007.pdf]

S7 Appendix Building thermodynamic models using the method of chemical potential

When MPRAs are performed for microbial systems, there are typically two possible approaches for delivering the mutant library into the cells, which are illustrated in Fig S13(A). In the first approach, the mutant sequence is directly integrated into the genome, which preserves the original copy number of the binding site. In the second approach, the mutant sequence is introduced into the cell using plasmids, which would contribute additional copies of the binding site. We would like to introduce additional binding sites into thermodynamic models in order to understand whether the methods of genome integration and using plasmid constructs will lead to different signals in the information footprints. However, the additional binding sites would lead to a combinatorial explosion that makes it impractical to write down all the states and the final probability of RNAP being bound. To tackle this challenge, we use an alternative method of constructing thermodynamic models using chemical potential.

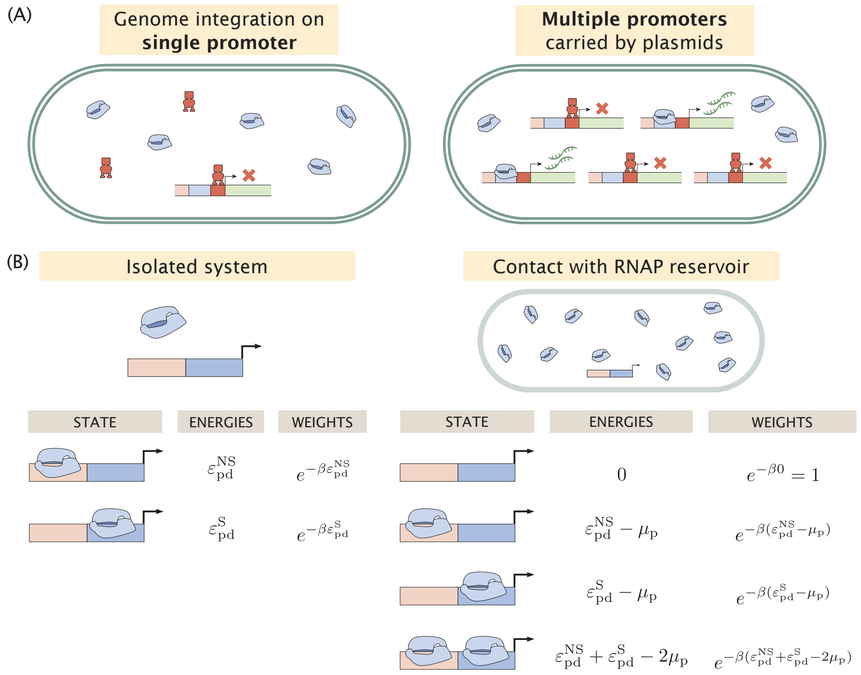

Fig S13. Changing the copy number of transcription factor binding sites. (A) There are two common ways to deliver sequence variants into a cell. If the promoter variant is integrated into the genome, the original copy number of the promoter is preserved. On the other hand, if the cells are transformed with plasmids containing the promoter variant, binding site copy number will increase. When the copy number of the binding site is high, the additional binding sites titrate away the repressors and the gene will be expressed at high levels despite the presence of repressors. (B) States-and-weights models for a constitutive promoter in an isolated system and in contact with a cellular reservoir of RNAPs. To build a thermodynamic model for an isolated system, we assume that there are no free-floating RNAPs and we require that the number of bound RNAPs is equal to the total number of RNAPs in the system. On the other hand, for a system that is in contact with a reservoir, we only need to ensure that the average number of RNAPs bound matches the total number of RNAPs.

Chemical potential corresponds to the free energy required to take an RNAP or a transcription factor out of the cellular reservoir. Under this conception, the total free energy of binding has two components: one is the specific binding energy $\varepsilon_{\rm pd}^{\rm NS}$ or $\varepsilon_{\rm pd}^{\rm NS}$, and one is the free energy μ_p that corresponds to the chemical potential. As shown in Fig S13(B), it is convenient to use chemical potential because in contrast to an isolated system, the resulting model no longer imposes a constraint on the exact number of RNAP or transcription factors bound to the promoter. Instead, we can tune the chemical potential such that we

constrain the *average* number of bound RNAPs and transcription factors. This method decouples the individual binding sites and allows us to write the total partition function as a product of the partition functions at each site.

To illustrates how this works, let us consider the states-and-weights diagram shown on the right of Fig S13(B). In this system, there are one specific RNAP binding site and one non-specific RNAP binding site. By assuming that the binding sites are in contact with the RNAP reservoir, we not only allow the states where a single RNAP is bound, but we also allow the states where no RNAP is bound and where two RNAPs are bound. In this way, the partition function can be written as

$$\mathcal{Z} = (1 + e^{-\beta(\varepsilon_{\text{pd}}^{\text{S}} - \mu_p)})(1 + e^{-\beta(\varepsilon_{\text{pd}}^{\text{NS}} - \mu_p)}). \tag{S49}$$

By extension, when there are M specific binding sites and N non-specific binding sites, the partition function can be written as

$$\mathcal{Z} = (1 + e^{-\beta(\varepsilon_{\text{pd}}^{\text{S}} - \mu_p)})^M (1 + e^{-\beta(\varepsilon_{\text{pd}}^{\text{NS}} - \mu_p)})^N. \tag{S50}$$

With this, we can write down the following expression for the average number of RNAPs bound to the M specific binding site

$$\langle n_{\text{S}} \rangle = \sum_{n_{\text{S}}=0}^{N} n_{\text{S}} \cdot \frac{\frac{M!}{n_{\text{S}}!(M-n_{\text{S}})!} e^{-\beta n_s(\varepsilon_{\text{S}} - \mu_p)} (1 + e^{-\beta(\varepsilon_{\text{NS}} - \mu_p)})^N}{\mathcal{Z}}, \tag{S51}$$

which can be simplified to

$$\langle n_{\text{S}} \rangle = M \frac{\lambda x}{1 + \lambda x}, \tag{S52}$$

where $\lambda = e^{\mu_p}$ and $x = e^{-\beta \varepsilon_{\text{pd}}^{\text{S}}}$. Similarly, the average number of RNAPs bound to the N non-specific binding sites is given by

$$\langle n_{\text{NS}} \rangle = N \frac{\lambda y}{1 + \lambda y}, \tag{S53}$$

where $y = e^{-\beta \varepsilon_{\text{pd}}^{\text{NS}}}$. Finally, we solve for λ using the following equation

$$P = \langle n_{\text{S}} \rangle + \langle n_{\text{NS}} \rangle = M \frac{\lambda x}{1 + \lambda x} + N \frac{\lambda y}{1 + \lambda y}, \tag{S54}$$

which allows us to impose the constraint on the average number of RNAPs.
