# Supplementary material for: Deciphering regulatory architectures of bacterial promoters from synthetic expression patterns
Source: PLoS Comput Biol. 2024 Dec 26;20(12):e1012697. doi: 10.1371/journal.pcbi.1012697 (PMC11709304; doi:10.1371/journal.pcbi.1012697)
Supplement: S8 Appendix — (PDF) [file pcbi.1012697.s008.pdf]

S8 Appendix Adding inducibility to thermodynamic models

S8.1 Probability of RNAP being bound under an inducible repressor

As shown in Fig S14(A), to include an inducible repressor in our thermodynamic model, we add an additional state that accounts for binding between the inactivated repressor and the promoter. This additional state is a weak binding state where the repressor is more likely to dissociate from the binding site. In many cases, transcription factors have multiple inducer binding sites. Here, we choose a typical model where the repressor has two inducer binding sites. Based on the new states-and-weights diagram, the probability of RNAP being bound can be rewritten according to the following expression [1]

$$p_{\text{bound}} = \frac{\frac{P}{N_{\text{NS}}} e^{-\beta \Delta \varepsilon_{\text{pd}}}}{1 + \frac{R_A}{N_{\text{NS}}} e^{-\beta \Delta \varepsilon_{\text{rd}}^A} + \frac{R_I}{N_{\text{NS}}} e^{-\beta \Delta \varepsilon_{\text{rd}}^I} + \frac{P}{N_{\text{NS}}} e^{-\beta \Delta \varepsilon_{\text{pd}}}}, \tag{S55}$$

where P is the number of RNAPs, R_A is the number of active repressors, R_I is the number of inactive repressors, and $\Delta\varepsilon_{\text{pd}}$, $\Delta\varepsilon_{\text{rd}}^A$, $\Delta\varepsilon_{\text{rd}}^I$ correspond to the energy differences between specific and non-specific binding of the RNAP, the active repressor, and the inactive repressor respectively.

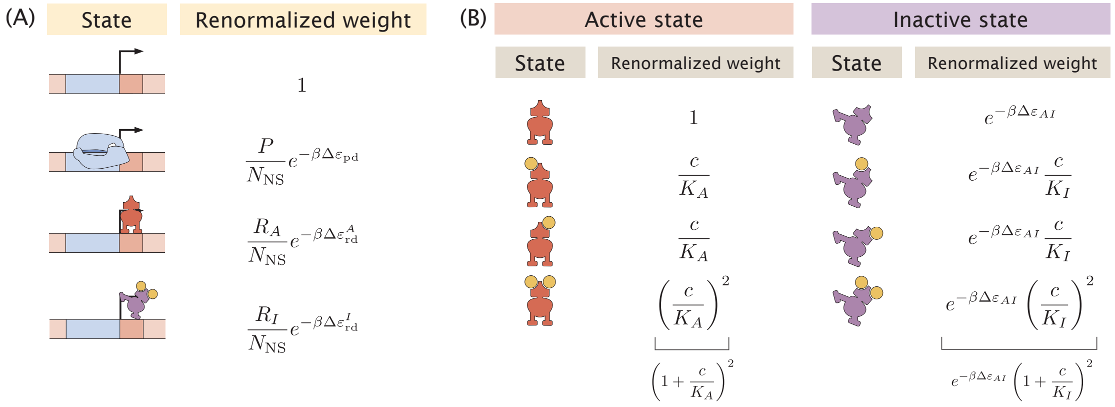

Fig S14. Changing the concentration of the inducer. (A) States-and-weights diagram for an inducible repressor. (B) States-and-weights diagram to calculate the probability that the repressor is in the active state.

In order to calculate the probability of RNAP being bound, we need to determine the proportion of R_A and R_I with respect to the total number of repressors. To do this, we calculate $p_{\text{active}}(c)$, the probability that the repressor exists in the active conformation as a function of the concentration of the inducer, c. To calculate $p_{\text{active}}(c)$, we model the different states of the repressor using another states-and-weights diagram, as illustrated in Fig 10(B). The probability that the repressor is in the active state is

$$p_{\text{active}}(c) = \frac{\left(1 + \frac{c}{K_A}\right)^2}{\left(1 + \frac{c}{K_A}\right)^2 + e^{-\Delta\varepsilon_{AI}/k_B T}\left(1 + \frac{c}{K_I}\right)^2}, \tag{S56}$$

where K_A is the dissociation constant between the inducer and the active repressor, K_I is the dissociation constant between the inducer and the inactive repressor, and $\Delta\varepsilon_{AI}$ is the structural energy difference between the active repressor and the inactive repressor. This allows us to represent the number of active and inactive repressors as $R_A = p_{\text{active}} R$ and $R_I = (1 - p_{\text{active}})R$. Therefore, our expression for p_{bound} can be modified to

$$p_{\text{bound}} = \frac{\frac{P}{N_{\text{NS}}} e^{-\beta \Delta \varepsilon_{\text{pd}}}}{1 + p_{\text{active}} \frac{R}{N_{\text{NS}}} e^{-\beta \Delta \varepsilon_{\text{rd}}^A} + (1 - p_{\text{active}}) \frac{R}{N_{\text{NS}}} e^{-\beta \Delta \varepsilon_{\text{rd}}^I} + \frac{P}{N_{\text{NS}}} e^{-\beta \Delta \varepsilon_{\text{pd}}}}. \tag{S57}$$

We use the above expression to calculate the probability of RNAP being bound in Fig 10.

S8.2 Changing inducer concentration for an inducible activator

In Sec 2.4, we discussed the effects of inducer concentration on the information footprints of a simple repression promoter with an inducible repressor. Similar effects can also be seen for a simple activation promoter with an inducible activator. One example of an inducible activator is CRP, which changes its conformation when bound to cyclic-AMP and thereby becomes more favorable to DNA binding [2]. Based on the states-and-weights diagram for such a promoter, which is shown in Fig S15(A), the probability of RNAP being bound is given by

$$p_{\text{bound}} = \frac{p + pa_A\omega_A + pa_I\omega_I}{1 + p + a_A + a_I + pa_A\omega_A + pa_I\omega_I}, \tag{S58}$$

where p is the normalized weight of the RNAP bound state, a_A is the normalized weight of the active activator bound state, and a_I is the normalized weight of the inactive activator bound state. ω_A and ω_I account for the interaction energy between the RNAP and the active activator and the interaction energy between the RNAP and the inactive activator, respectively. The exact expressions for p, a_A, a_I, ω_A and ω_I are given in Fig S15(A).

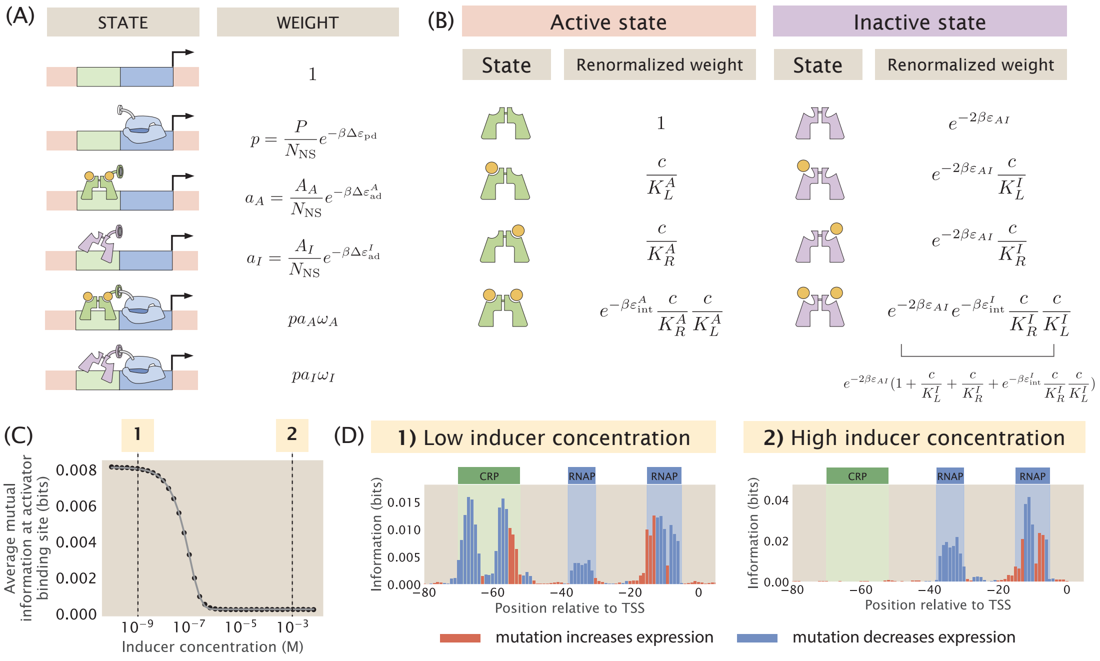

Fig S15. Changing inducer concentration for the inducible activator. (A) States-and-weights diagram for an inducible activator. In the diagram, N_{NS} is the number of non-binding sites in the genome, P is the copy number of the RNAP, A_A is the copy number of active activators, A_I is the copy number of inactive activators, $\Delta\varepsilon_{\text{pd}}$ is the binding energy of the RNAP, $\Delta\varepsilon^A_{\text{ad}}$ is the binding energy of the active activator, $\Delta\varepsilon^I_{\text{ad}}$ is the binding energy of the inactive activator. $\omega_A = e^{-\beta\varepsilon_{p,a_A}}$ and $\omega_I = e^{-\beta\varepsilon_{p,a_I}}$, where ε_{p,a_A} is the interaction energy between the RNAP and the active activator and ε_{p,a_I} is the interaction energy between the RNAP and the inactive activator. (B) States-and-weights diagram to calculate the probability that the activator is in the active state. (C) Average mutual information at the activator binding site increases as the inducer concentration increases. Here, we let $K^A_L = K^A_R = 3 \times 10^{-6}$ M, $K^I_L = K^A_R = 10^{-7}$ M, and $\Delta\varepsilon_{AI} = -2\ k_BT$ [2]. Each data point is the mean of average mutual information across 20 synthetic datasets with the corresponding inducer concentration. The numbered labels correspond to footprints in (D). (D) Representative information footprints with low inducer concentration (10^{-9} M) and high inducer concentration (10^{-3} M).

To simplify the expression above, we determine the proportion of active and inactive activators with respect to the total number of activators. Similar to the case of simple repression, we calculate $p_{\text{active}}(c)$.

The different states of the activator can be modelled using the states-and-weights diagram shown in Fig S11(B). Here, we consider two types of cooperativity. The first type of cooperativity is between the two binding sites, where each ligand binding event changes the binding affinity of the unbound site. This is inherent to the classic MWC model and is already encoded in the terms ω_A and ω_I in Eq S58. The second type of cooperativity is between the two ligands, which accounts for the negative cooperativity of CRP in the inactive state. This is accounted for by the cooperative energy terms $\varepsilon_{\text{int}}^A$ and $\varepsilon_{\text{int}}^I$, which represent the interaction energies between the two ligands in the active and inactive states, respectively. Given the states-and-weights diagram, $p_{\text{active}}(c)$ is given by

$$p_{\text{active}}(c) = \frac{1 + \frac{c}{K_L^A} + \frac{c}{K_R^A} + \frac{c}{K_L^A}\frac{c}{K_R^A}e^{-\beta\varepsilon_{\text{int}}^A}}{1 + \frac{c}{K_L^A} + \frac{c}{K_R^A} + \frac{c}{K_L^A}\frac{c}{K_R^A}e^{-\beta\varepsilon_{\text{int}}^A} + e^{-2\beta\varepsilon_{AI}}(1 + \frac{c}{K_L^I} + \frac{c}{K_R^I} + \frac{c}{K_L^I}\frac{c}{K_R^I}e^{-\beta\varepsilon_{\text{int}}^I})}, \tag{S59}$$

where K_L^A is the dissociation constant between the inducer and the left binding pocket of the active activator, K_R^A is the dissociation constant between the inducer and the right binding pocket of the active activator, K_L^I is the dissociation constant between the inducer and the left binding pocket of the inactive activator, and K_R^I is the dissociation constant between the inducer and the right binding pocket of the inactive activator. With this expression, we can represent the number of active and inactive activators as $A_A = p_{\text{active}}A$ and $A_I = (1 - p_{\text{active}})A$. Therefore, we have that $a_A = p_{\text{active}}\frac{A}{N_{\text{NS}}}e^{-\beta\Delta\varepsilon_{ad}^A}$ and $a_I = (1 - p_{\text{inactive}})\frac{A}{N_{\text{NS}}}e^{-\beta\Delta\varepsilon_{ad}^I}$.

We built synthetic datasets for a simple activation promoter with an inducible activator. As shown in Fig S15(C) and Fig S15(D), when the concentration of the inducer is increased, the average signal at the activator binding site decreases and subsequently plateaus. At high inducer concentration, the activator is too strongly bound to be affected by mutations, and therefore the signal at the activator binding site is negligible.

SI references

1. Razo-Mejia M, Barnes SL, Belliveau NM, Chure G, Einav T, Lewis M, and Phillips R. Tuning Transcriptional Regulation through Signaling: A Predictive Theory of Allosteric Induction. Cell Syst 2018 Apr; 6:456–469.e10

2. Einav T, Duque J, and Phillips R. Theoretical analysis of inducer and operator binding for cyclic-AMP receptor protein mutants. PLoS One 2018 Sep; 13:e0204275
