# Supplementary material for: Deciphering regulatory architectures of bacterial promoters from synthetic expression patterns
Source: PLoS Comput Biol. 2024 Dec 26;20(12):e1012697. doi: 10.1371/journal.pcbi.1012697 (PMC11709304; doi:10.1371/journal.pcbi.1012697)
Supplement: S9 Appendix — (PDF) [file pcbi.1012697.s009.pdf]

S9 Appendix Noise from experimental procedures of MPRAs

In MPRAs such as Reg-Seq, the mutant library is grown up in culture. Once the cell culture is prepared, genomic DNA (gDNA) and mRNAs are extracted, the latter of which is used as a template in reverse transcription to make complementary DNA (cDNA). Afterwards, polymerase chain reation (PCR) is performed to amplify the reporter gene from the gDNA and cDNA. Finally, sequencing adapters are attached to the gDNA and cDNA. The gDNA and cDNA are then sequenced to obtain DNA and RNA counts for each sequence variant.

As illustrated in Fig S16(A), there are at least two possible sources of experimental noise in this procedure. Firstly, PCR amplification is a stochastic process where the probability that a DNA molecule is amplified in a given cycle is less than one. This stochasticity may cause some sequences to have an artificially high RNA count. We note that assuming that the same reporter gene is used for each sample, the only difference in the sequence being amplified would be the barcode. Since barcodes are typically much shorter, it is unlikely to significantly alter the GC-content of the sequence, and therefore unlikely to significantly bias amplification. Secondly, during RNA-Seq as well as the prior library preparation procedures such as RNA extraction and reverse transcription, we cannot ensure that every mRNA is extracted, converted to cDNA, and sequenced. Instead, in these steps, only a random subset of the original pool of mRNAs is sampled and included in the final sequencing dataset. As a result, a sequence may have an artificially low RNA count because some copies of the mRNA associated with that sequence are not sampled in one of the experimental steps.

We simulate these two sources of experimental noise in our computational pipeline. To simulate PCR with n cycles of amplification, we start with the original mRNA counts predicted based on the probability of RNAP being bound. Subsequently, we model the number of sequences that are successfully amplified during each cycle using a binomial distribution [1]. Hence, for each sequence variant,

$$n(j+1) = n(j) + B(n(j), P_{\text{amp}}), \tag{S60}$$

where $n(j)$ is the number of sequences of the promoter variant in cycle j, $B(n, P)$ is a binomial random variable, and P_{amp} is the probability that a sequence is successfully amplified in a cycle. We applied Eq S60 to calculate the final count of each sequence variant in a library. As shown in Fig S16(B) and S16(D), even when the probability of amplification is set to a low value of $P_{\text{amp}} = 0.5$, increasing the number of PCR cycles does not reduce the signal-to-noise ratio in information footprints. Therefore, we conclude that stochasticity in PCR does not contribute to significant levels of noise in information footprints.

To simulate the random sampling effect during RNA extraction, reverse transcription, and sequencing, we randomly draw a subset of promoter variants in the mutant library and we only consider the expression levels of the selected promoter variants when we calculate mutual information to build the information footprint. As shown in Fig S16(C) and S16(D), the resulting noise only becomes significant when less than 1% of the original pool of sequences is sampled. Therefore, random sampling effects are not a significant source of noise in information footprints either.

SI references

1. Kebschull JM and Zador AM. Sources of PCR-induced distortions in high-throughput sequencing data sets. Nucleic Acids Res. 2015 Dec; 43:e143

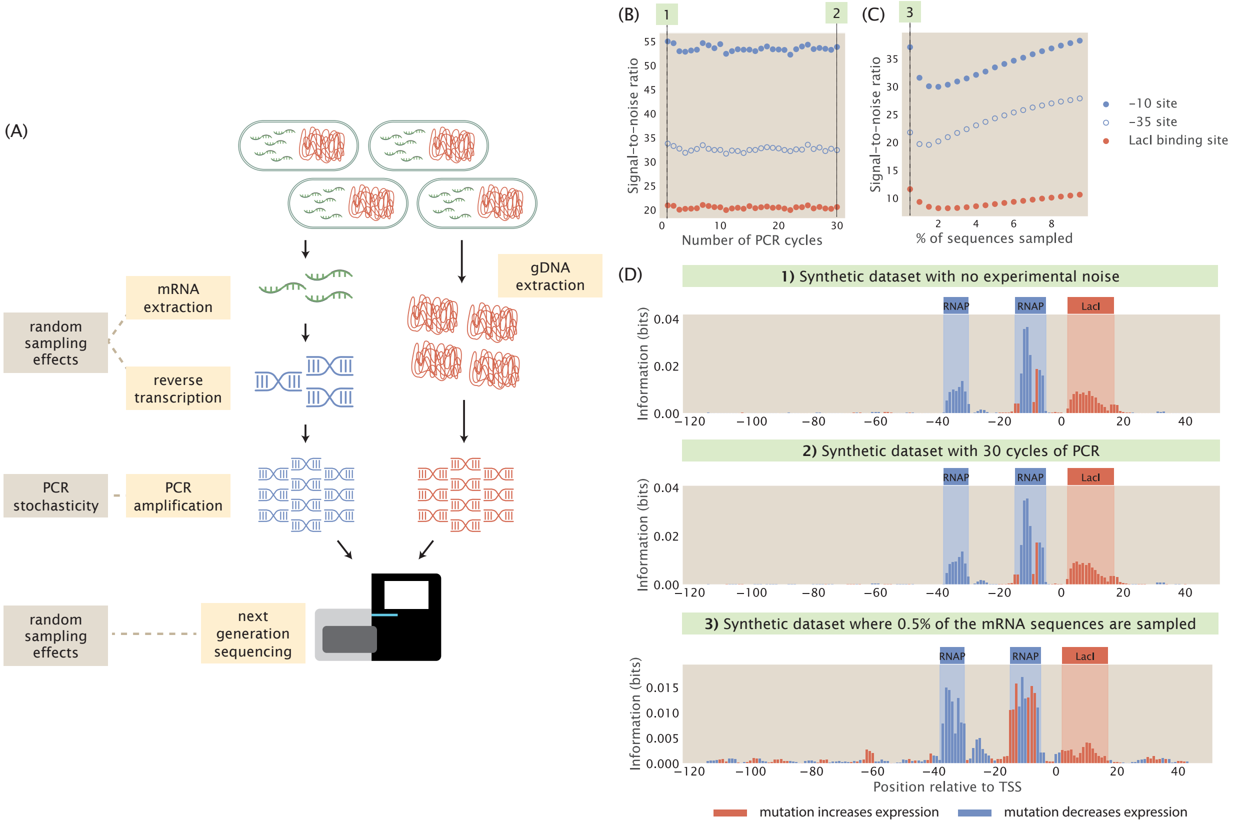

Fig S16. Noise from experimental procedures in the Reg-Seq pipeline. (A) The two main sources of noise in the experimental MPRA pipeline are stochasticity from PCR amplification and random sampling effects from RNA extraction, reverse transcription, and RNA-Seq. (B) Signal-to-noise ratio in the information footprints remains high when the number of PCR amplification cycles is increased. Here, $P_{\text{amp}} = 0.5$. Each data point is the mean of average mutual information across 20 synthetic datasets with the corresponding number of PCR cycles. The numbered labels correspond to footprints in (D). (C) Signal-to-noise ratio remains high when only a small percentage of the sequences are randomly sampled. Each data point is the mean of average mutual information across 20 synthetic datasets with the corresponding percentage of sampled sequences. The numbered labels correspond to footprints in (D). (D) Representative information footprints with no experimental noise, PCR stochasticity after 30 cycles, and random sampling effects after 0.5% of the RNA sequences are sampled.
