# Supplementary material for: Deciphering regulatory architectures of bacterial promoters from synthetic expression patterns
Source: PLoS Comput Biol. 2024 Dec 26;20(12):e1012697. doi: 10.1371/journal.pcbi.1012697 (PMC11709304; doi:10.1371/journal.pcbi.1012697)
Supplement: S10 Appendix — (PDF) [file pcbi.1012697.s010.pdf]

S10 Appendix Modelling extrinsic noise in transcription initiation

S10.1 Describing extrinsic noise using a Log-Normal distribution

In order to account for extrinsic noise, we choose to use a Log-Normal distribution to describe the copy number of RNAPs and repressors. Let X be the copy number of the RNAP or the transcription factor, we define the Log-Normal distribution as

$$\log X \sim \text{Normal}\left(\log \mu, (\alpha \log \mu)^2\right). \tag{S61}$$

Since the goal of our analysis is to better formulate regulatory hypotheses based on real world data, we focus on levels of copy number fluctuations that are physiologically relevant. Assuming that the extrinsic noise in copy numbers primarily comes from asymmetrical partitioning during cell division, one method to measure fluctuations in transcription factor copy numbers between cells is the dilution method developed by Rosenfeld, Young et al. [1]. Based on Brewster et al. [2] who utilized the dilution method, transcription factor copy numbers typically vary by less than 20% of the mean copy number. Consider the proteomic measurements from Schmidt et al. [3] and Balakrishnan et al. [4], the coefficient of variation for transcription factor copy numbers is less than 2 even across very different growth conditions. With these empirical data, we can then define our distributions of copy numbers to respect the known levels of fluctuation. Given the known mean and variance of a Log-Normal distribution, we can derive that the coefficient of variation is given by

$$\text{CoV}(X) = \frac{\mathbb{E}(X)}{\text{Var}X} = \sqrt{e^{(\alpha \log \mu)^2} - 1}.$$

Rearranging this expression, we can write down α in terms of μ and $\text{CoV}(X)$, where

$$\alpha = \frac{\sqrt{\log\left[\text{CoV}(X)^2 + 1\right]}}{\log \mu}$$

This means that we can derive a Log-Normal distribution that obeys the empirical mean and coefficient of variation for transcription factors. In Fig 12, we simulated noisy synthetic datasets using Log-Normal distributions with $P = 5000$ and $R = 100$ as the mean copy numbers and a range of coefficients of variation from 0.1 to 10^2, which covers the levels of fluctuations that are physiologically relevant. In Fig 12, we show the distributions of copy numbers given the three levels of fluctuations that we particularly discuss in Sec 3.1. Note that when the coefficient of variation is set to 100, the copy number of RNAPs can reach as high as 10^9 and the copy numbers of repressors can reach as high as 10^7, both of which are unrealistic.

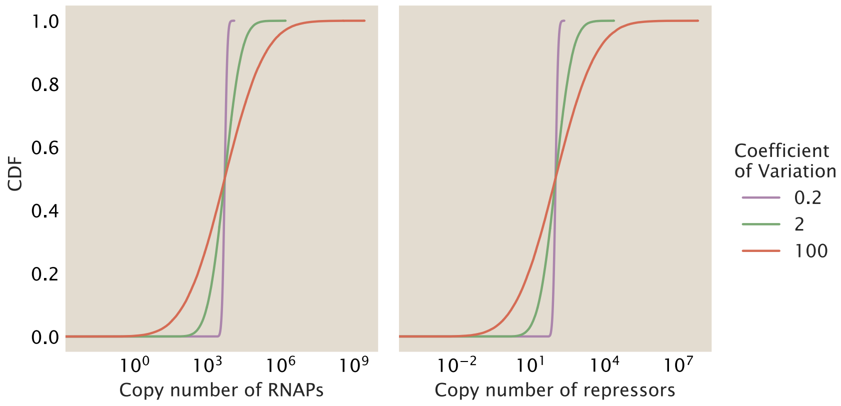

Fig S17. Modelling the copy number of RNAPs and repressors using a Log-Normal distribution. Cumulative distribution functions (CDFs) for the copy numbers of RNAP and repressors modelled using a Log-Normal distribution and under three different levels of coefficient of variation.

S10.2 Extrinsic noise under low signal

In Sec 3.1, we explored the effect of extrinsic noise while keeping all other parameter at their standard values. That is to say, we expect that the fluctuations in copy numbers should not drastically change the level of signal in the footprints. However, one reasonable suspicion is that when the signal from binding events is sufficiently low, even low levels of noise may affect our interpretation of information footprints and expression shift matrices. To examine whether this is the case, we built footprints with lowered binding energy for the repressor while allowing copy numbers to fluctuate. As shown in Fig S18, even when the signal is low and the coefficient of variation is set to as high as 100, we can still identify signal at the repressor binding site. Therefore, it seems that this is not an issue unless the level of noise is unrealistically high.

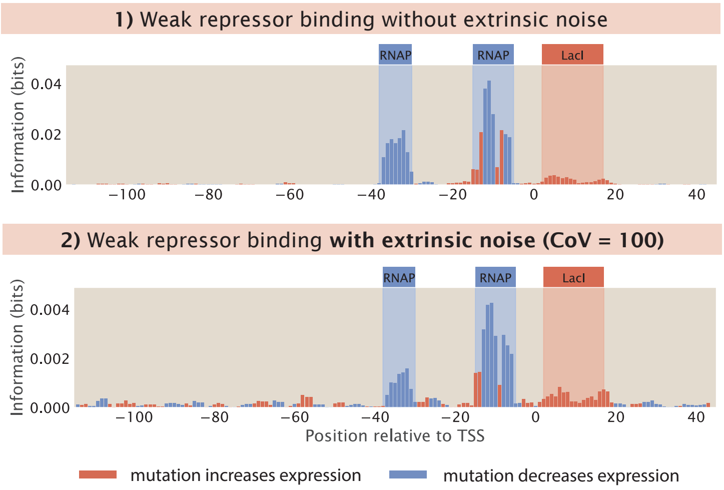

Fig S18. Effects of extrinsic noise on information footprints with weak repressor binding. For both footprints, the repressor binding energy $\Delta\varepsilon_{\mathrm{rd}} = -11\, k_B T$. In the top information footprint, no extrinsic noise is introduced. In the bottom information footprint, the Log-Normal distribution from which RNAP and repressor copy numbers are drawn has a coefficient of variation of 100.

S10.3 Extrinsic noise for different architectures

To see if our investigation of extrinsic noise is generalizable, we test the cases where there are copy number fluctuations under the other four common regulatory architectures (simple activation, double repression, double activation, and repression-activation). As shown in Fig S19, the signal-to-noise ratio remains high regardless of the architecture even when copy numbers are allowed to fluctuate to 10 times above or below the average copy number.

SI references

1. Rosenfeld N, Young JW, Alon U, Swain PS, and Elowitz MB. Accurate prediction of gene feedback circuit behavior from component properties. Mol. Syst. Biol. 2007 Nov; 3:143
2. Brewster RC, Weinert FM, Garcia HG, Song D, Rydenfelt M, and Phillips R. The transcription factor titration effect dictates level of gene expression. Cell 2014 Mar; 156:1312–23
3. Schmidt A, Kochanowski K, Vedelaar S, Ahrné E, Volkmer B, Callipo L, Knoops K, Bauer M, Aebersold R, and Heinemann M. The quantitative and condition-dependent *Escherichia coli* proteome. Nat. Biotechnol. 2016 Jan; 34:104–10
4. Balakrishnan R, Mori M, Segota I, Zhang Z, Aebersold R, Ludwig C, and Hwa T. Principles of gene regulation quantitatively connect DNA to RNA and proteins in bacteria. Science 2022 Dec; 378:eabk2066

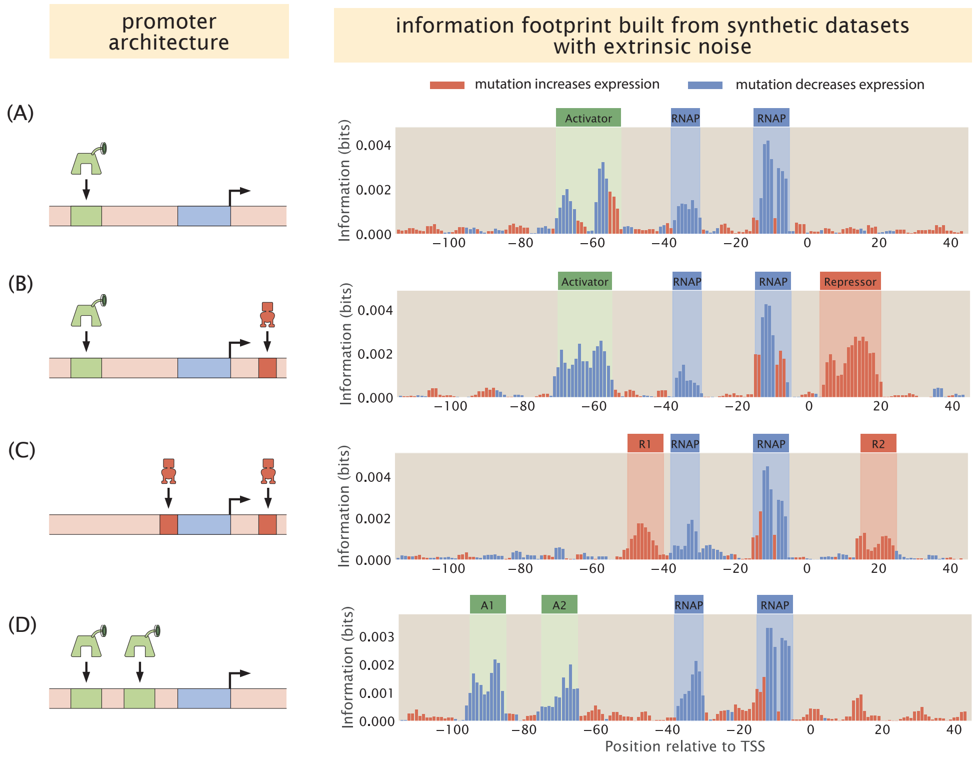

Fig S19. Information footprints built from synthetic datasets with extrinsic noise under common regulatory architectures. In all four plots, the coefficient of variation for RNAP and transcription copy numbers is set to 10. Panels (A) to (D) show the footprints from the simple activation architecture, repression-activation architecture, double repression architecture, and the double activation architecture, respectively.
