# Supplementary material for: Deciphering regulatory architectures of bacterial promoters from synthetic expression patterns
Source: PLoS Comput Biol. 2024 Dec 26;20(12):e1012697. doi: 10.1371/journal.pcbi.1012697 (PMC11709304; doi:10.1371/journal.pcbi.1012697)
Supplement: S11 Appendix — (PDF) [file pcbi.1012697.s011.pdf]

S11 Appendix Calculating the probability of transcriptionally active states under non-equilibrium

S11.1 General protocol for calculating the probability of transcriptionally active states with broken detailed balance

Let us consider a promoter with the simple activation regulatory architecture. Recall that such a promoter can be in one of four possible states: empty (E), bound by RNAP (P), bound by the activator (A), or bound by both RNAP and the activator (AP). As shown in Fig S20, we can describe this architecture using a directed square graph with four vertices and eight edges. Each vertex corresponds to one of the four states; each edge describes the transition between two connected states and is associated with a rate constant. Having written down the architecture using a graph, the probability of each possible state can be derived using the Matrix Tree Theorem, which states that the probability of a given state at steady state is proportional to the sum of products of rate constants across all spanning trees that are rooted in that state. The expression for the probability of each state is given in Appendix S11.2. With this, we can write down the total probability of the transcriptionally active states

$$p_{\text{active}} = p_{\text{A}} + p_{\text{AP}}, \tag{S62}$$

where p_{A} is the probability that the activator is bound to the promoter and p_{AP} is the probability that both the activator and RNAP are bound to the promoter.

Importantly, to construct a synthetic MPRA dataset with broken detailed balance, the sequence-dependence of p_{active} needs to be preserved. Here, we make the simplifying assumption that all the on-rates are diffusion limited and therefore independent of promoter sequence. This means that the sequence-dependence of p_{active} comes from the mapping between the off rates and the sequences of the promoter variants. One way to create this mapping is to leverage the fact that the dissociation constant K_d is sequence-dependent. Specifically, we have that

$$K_d = c_0\, e^{\beta \Delta\varepsilon}, \tag{S63}$$

where $c_0 = 1\ M$ is the reference concentration of the standard state. As we have demonstrated in Fig 3(A), $\Delta\varepsilon_D$ can be calculated using energy matrices in a sequence-dependent manner, which confers sequence-specificity to K_d. Since $k_{\text{off}}^{\text{eq}} = k_{\text{on}}^{\text{eq}} \times K_d$, we can also calculate k_{off} in a sequence-dependent manner. To demonstrate that the graph-theoretic approach allows us to build synthetic datasets, we first use the method outlined above to calculate k_{on} and k_{off} for each promoter variant at equilibrium and used these values to predict the expression levels of the promoter variants with the simple activation regulatory architecture.

Finally, to build a synthetic dataset under non-equilibrium, we can estimate k_{off} by incorporating the energy U invested to break detailed balance, where

$$k_{\text{off}} = k_{\text{off}}^{\text{eq}} \times e^{\beta U}. \tag{S64}$$

The diffusion-limited k_{on} and the sequence-dependent k_{off} for each edge can then be used to calculate p_{active} for each promoter variant and predict the expression levels of mutant promoters under non-equilibrium conditions. For example, we can break detailed balance at the edge where RNAP unbinds from the state where both the activator and RNAP are bound. That is to say, we invest an energy $U_{\text{AP,A}}$ such that

$$k_{\text{AP,A}} = k_{\text{AP,A}}^{\text{eq}} \times e^{\beta U_{\text{AP,A}}}. \tag{S65}$$

With these sequence-dependent rate coefficients, we can then calculate p_{active} for each of the mutant sequences under non-equilibrium.

S11.2 Statistical weights for the simple activation promoter based on spanning trees

To derive the statistical weights of all states using the graph-theoretic approach introduced in Appendix S11.2, we make use of the Matrix Tree Theorem, which states that at steady state, the probability

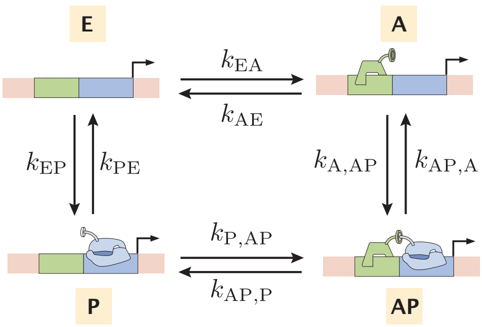

Fig S20. Directed square graph that describes the kinetic processes of a simple activation promoter. State E corresponds to the state where the promoter is not bound by either the RNAP or the activator. State P corresponds to the state where the RNAP is bound to the promoter. State A corresponds to the state where the activator is bound to the promoter. State AP corresponds to the state where both the activator and the RNAP are bound to the promoter.

of state i is proportional to the sum of products of rate constants across all spanning trees rooted in the vertex representing state i. By definition, a spanning tree rooted in vertex i is a subgraph that (1) contains all vertices in the original graph and (2) has all edges incoming in vertex i. The graph describing a simple activation promoter is shown in Fig 15. As shown in Fig S21, we can enumerate all spanning trees for each of the four vertices. This gives us the following statistical weights for each of the promoter states

$$\rho_{\mathrm{E}} = k_{\mathrm{AE}}\, k_{\mathrm{PE}}\, k_{\mathrm{AP,P}} + k_{\mathrm{A,AP}}[\mathrm{P}]\, k_{\mathrm{AP,P}}\, k_{\mathrm{PE}} + k_{\mathrm{PE}}\, k_{\mathrm{AP,P}}\, k_{\mathrm{AE}} + k_{\mathrm{P,AP}}[\mathrm{A}]\, k_{\mathrm{AP,P}}\, k_{\mathrm{AE}} \tag{S66}$$
$$\rho_{\mathrm{P}} = k_{\mathrm{AE}}\, k_{\mathrm{EP}}[\mathrm{P}]\, k_{\mathrm{AP,P}} + k_{\mathrm{EP}}[\mathrm{P}]\, k_{\mathrm{A,AP}}[\mathrm{P}]\, k_{\mathrm{AP,P}} + k_{\mathrm{AP,A}}\, k_{\mathrm{AE}}\, k_{\mathrm{EP}}[\mathrm{P}] + k_{\mathrm{EA}}\,[\mathrm{A}]\, k_{\mathrm{A,AP}}[\mathrm{P}]\, k_{\mathrm{AP,P}} \tag{S67}$$
$$\rho_{\mathrm{A}} = k_{\mathrm{AP,P}}\, k_{\mathrm{PE}}\, k_{\mathrm{EA}}[\mathrm{A}] + k_{\mathrm{EP}}[\mathrm{P}]\, k_{\mathrm{P,AP}}[\mathrm{A}]\, k_{\mathrm{AP,A}} + k_{\mathrm{PE}}\, k_{\mathrm{EA}}[\mathrm{A}]\, k_{\mathrm{AP,A}} + k_{\mathrm{P,AP}}[\mathrm{A}]\, k_{\mathrm{AP,A}}\, k_{\mathrm{EA}}[\mathrm{A}] \tag{S68}$$
$$\rho_{\mathrm{AP}} = k_{\mathrm{AE}}\, k_{\mathrm{EP}}[\mathrm{P}]\, k_{\mathrm{P,AP}}[\mathrm{A}] + k_{\mathrm{EP}}[\mathrm{P}]\, k_{\mathrm{P,AP}}[\mathrm{A}]\, k_{\mathrm{A,AP}}[\mathrm{P}] + k_{\mathrm{PE}}\, k_{\mathrm{EA}}[\mathrm{A}]\, k_{\mathrm{A,AP}}[\mathrm{P}] + k_{\mathrm{EA}}[\mathrm{A}]\, k_{\mathrm{A,AP}}[\mathrm{P}]\, k_{\mathrm{P,AP}}[\mathrm{A}] \tag{S69}$$

Finally, we can calculate the probability of each state by taking the weight of each state and dividing by the sum of all weights

$$p_{\mathrm{E}} = \frac{\rho_{\mathrm{E}}}{\rho_{\mathrm{E}} + \rho_{\mathrm{P}} + \rho_{\mathrm{A}} + \rho_{\mathrm{AP}}} \tag{S70}$$
$$p_{\mathrm{P}} = \frac{\rho_{\mathrm{P}}}{\rho_{\mathrm{E}} + \rho_{\mathrm{P}} + \rho_{\mathrm{A}} + \rho_{\mathrm{AP}}} \tag{S71}$$
$$p_{\mathrm{A}} = \frac{\rho_{\mathrm{A}}}{\rho_{\mathrm{E}} + \rho_{\mathrm{P}} + \rho_{\mathrm{A}} + \rho_{\mathrm{AP}}} \tag{S72}$$
$$p_{\mathrm{AP}} = \frac{\rho_{\mathrm{AP}}}{\rho_{\mathrm{E}} + \rho_{\mathrm{P}} + \rho_{\mathrm{A}} + \rho_{\mathrm{AP}}} \tag{S73}$$

In particular, since A and AP are the transcriptionally active states, the probability that the promoter is on is given by $p_{\mathrm{active}} = p_{\mathrm{A}} + p_{\mathrm{AP}}$.

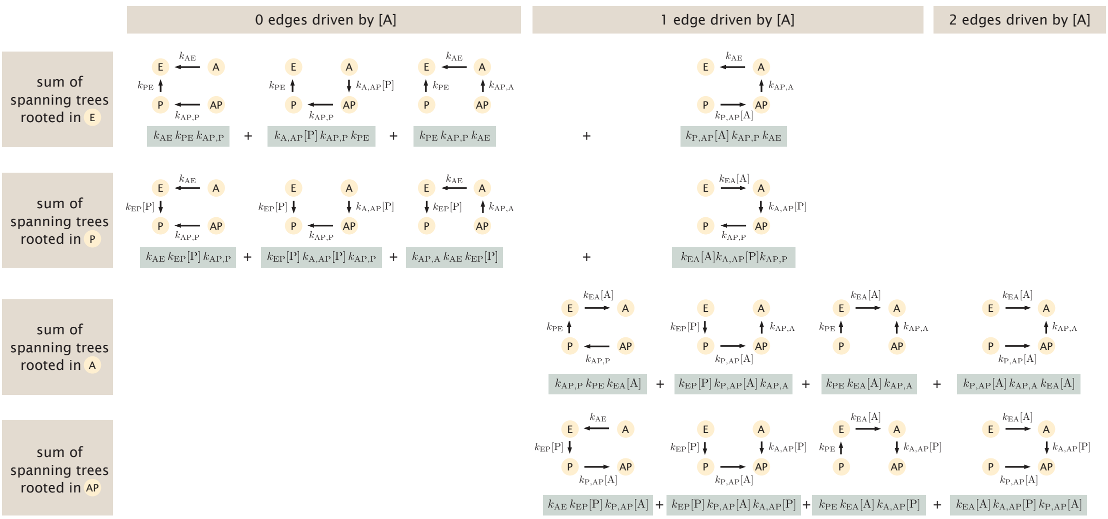

Fig S21. Deriving statistical weights of promoter states using spanning trees. Each row corresponds to a different root for the spanning trees. The columns are grouped based on the number of edges in the spanning tree that depend upon the concentration of the activator. The figure is adapted from Mahdavi, Salmon et al. [1].

SI references

1. Mahdavi SD, Salmon GL, Daghlian P, Garcia HG, and Phillips R. Flexibility and sensitivity in gene regulation out of equilibrium. Proc. Natl. Acad. Sci. U. S. A. 2024 Nov; 121:e2411395121
