# Supplementary material for: Deciphering regulatory architectures of bacterial promoters from synthetic expression patterns
Source: PLoS Comput Biol. 2024 Dec 26;20(12):e1012697. doi: 10.1371/journal.pcbi.1012697 (PMC11709304; doi:10.1371/journal.pcbi.1012697)
Supplement: Attachment — Submitted filename: review_wenshan_pan.pdf [file pcbi.1012697.s012.pdf]

Wenshan pan et al. present a theoretical study to infer the function of regulatory sites in promoters from data from massively parallel reporter assays (MPRA) using the so-called thermodynamic equilibrium model of gene regulation. The paper is clearly written and the results all seem valid (apart from some small errors that we noticed, as detailed below) and I do not have much to object to the contents or the interpretations of the results provided. However, two main points temper my enthusiasm regarding this work:

1. The corresponding author has published a whole host of papers already on the thermodynamic equilibrium model approach to transcription regulation (in particular for prokaryotes) including papers on the various effects explored here (e.g. the titration effect paper of Brewster et al) as well as on the use of this approach in the context of MPRA (e.g. in collaboration with Justin Kinney). Given this existing literature, I struggled to identify what is really novel here. I first thought that maybe an integrated computational tool would be presented but it doesn't look like that to me. It also was not obvious to me what qualitatively new effects were being explored, or how the theory presented goes beyond what is already in the literature. I guess the paper could maybe be of value as an introduction to the thermodynamic model of prokaryotic gene regulation and interpretation of MPRA data for students that are starting in this field. But this is maybe a bit thin for a paper in PLoS Comp Biol. I would suggest that the authors revise the paper so as to focus on the aspects that they consider novel and bring these out more clearly.
2. I do not object to the thermodynamic equilibrium modeling that is used here, but I think it should be made clear that this is an *extremely simplified* model of what actually happens inside prokaryotic cells and that we currently do not know whether it is actually generally a quantitatively accurate model. Cells are typically growing and expanding exponentially, their DNA is replicated (which interferes with transcription), gene copy numbers vary as the DNA is replicated (and there has been recent evidence that this drives variations in expression at the single cell level), levels of TFs may be fluctuating on time scales that are not necessarily slow compared to the time scales of transcription, translation and dilution of proteins (which invalidates the thermodynamic model's assumptions). There are all kinds of non-equilibrium effects in the process of transcription and translation (eg. just the fact that RNA polymerase leaving the promoter when it starts transcribing is an irreversible non-equilibrium process, but also the super-coiling resulting from transcription, just to give two arbitrary examples). Although I agree that the equilibrium model that assumes transcription rate is proportional to equilibrium polymerase occupancy is a good starting model, we actually have no idea yet of how valid this model is *generally* for quantitatively describing gene regulation in prokaryotes. Given this, I am more than a little annoyed at the really grandiose tone in this manuscript, starting from the title. There is virtually zero acknowledgment in the manuscript that this model is a highly simplified model that may well not be appropriate in many situations. This needs to change. The authors need to point out how strong the simplifications are that are being made and that this is just a reasonable starting model and that we do not know yet whether it is generally quantitatively accurate.

I have a few more minor concerns which mainly consider cases where the authors seem to be drawing stronger and more general conclusions than I think can be supported by what is shown, and also some things that were unclear:

1. What are the `states' i in equations (3) and (4)? Are these different sigma70 binding sites? (or maybe also alternative sigma factor sites). And what is the mechanistic interpretation of having different rates r_i at these different sites?
2. Lines 131-135. I understand that these simplifications (only two bins and only mutation vs. wild-type) will generally increase mutual information, but they obviously also lose information in that we can no longer distinguish the effects of different letters nor distinguish small from large

changes in expression. So I do not think this simplification is always ‘optimal’. This warrants more discussion.

3. AND and OR models for function of promoters with two repressor sites. There should be more discussion that these strict AND and OR functions are an idealizations (which may not be anywhere close to how repressor sites actually interact in real promoters) and more generally, that we do not really know how to model the interactions between regulatory sites if there are more sites per promoter (i.e. how the transcription rate depends on the precise binding configuration of the TFs). There are plenty of promoters in *E. coli* which can have 5 or more binding sites and multiple sigma sites. It would be good for the authors to acknowledge that it is not obvious how the methods they present can be extended to such more complex situations.
4. In equation (15) the authors derive an ‘optimal’ mutation rate by taking the parameters of the LacI site and sigma70 site at the lac promoter and working out a mutation rate that puts these sites on equal footing. But the expression for kappa (equation (12)) depends on several parameters that can be very different at different binding sites and promoters and it is not even guaranteed that there is a mutation rate that makes the expected effect of mutations on different sites equal. I therefore find it quite misleading that this is presented as if it were some general optimization procedure.
5. Figure 8B, bottom panel. I do not understand that the curves do not intersect at 25 copies because in that case the situation is completely symmetric and both repressors should have the same mutual information. This makes me doubt this plot is correct.
6. Section 3.1. I understand that for the particular example studied here (i.e. the Lac promoter once again) the extrinsic noise did not have a large effect on the signal-to-noise, but that cannot *generally* be the case. If there are TF binding sites whose effects are weak enough and the noise is large enough, surely at some point the noise will become larger than the signal.
7. Section 3.2. A similar comment applies here. Not every promoter has a clear dominant sigma site and in some cases it might be hard to even clearly distinguish ‘spurious’ from ‘functional’ binding.
8. Section 3.3 shows that overlapping sites are difficult to interpret. But aren’t overlapping sites highly common in *E. coli* promoters? (especially if weaker sites are included). This warrants some discussion.

Finally, there were a number of places where I noted small errors/typos:

1. In figure 7b the panel titles should be swapped – the upper one depicts the case where $\Delta\varepsilon_{rd} = -10k_BT$, while in the bottom one $\Delta\varepsilon_{rd} = -20k_BT$.
2. In the denominator of equation (39), $\frac{R_A}{N_{NS}} e^{-\beta\Delta\varepsilon_{rd}^I}$ should be replaced by $\frac{R_A}{N_{NS}} e^{-\beta\Delta\varepsilon_{rd}^A}$.
3. In Figure 8c 5), R1=5 instead of R1=0.
4. Equation (42) seems incorrect. The numerator should not be 1 but the same as the numerator of equation (41).
5. Line 424 – concentration of the inducer goes up to $10K_A$ instead of K_A
6. In line 305, ‘strong binding’ should be replaced by ‘weak binding’.
7. Line 322 – it should state when R1<R2 instead of when R1>R2.
