# Supplementary material for: Deciphering regulatory architectures of bacterial promoters from synthetic expression patterns
Source: PLoS Comput Biol. 2024 Dec 26;20(12):e1012697. doi: 10.1371/journal.pcbi.1012697 (PMC11709304; doi:10.1371/journal.pcbi.1012697)
Supplement: Attachment — Submitted filename: response_to_reviewers.pdf [file pcbi.1012697.s013.pdf]

Thank you very much for submitting your manuscript "Dissecting endogeneous genetic circuits from first principles" for consideration at PLOS Computational Biology.

As with all papers reviewed by the journal, your manuscript was reviewed by members of the editorial board and by several independent reviewers. In light of the reviews (below this email), we would like to invite the resubmission of a significantly-revised version that takes into account the reviewers' comments.

Author's response: We are deeply grateful to both reviewers and the editor for their careful engagement with our paper. The act of responding to their comments significantly improved our work and as a result, we hope that it will now be ready for publication.

We regret that our manuscript failed to convey the philosophy and impact of our work and its tight coupling to huge high-throughput datasets for which there is no currently existing theory. The reviews have resulted in a massive rewrite as well as the addition of a variety of new results that go well beyond what was here before. We also argue that not only were there points of miscommunication on our part and perhaps some misunderstanding on the part of the reviewers, but we also believe that there are some elements of the discussion between us and the reviewers that are best served by careful analysis of previous literature. We hope that both the editor and the reviewers will be willing to engage in a more nuanced way with the role of thermodynamic models in the study of transcription and their value as a null model that serves as a point of departure in the analysis of huge datasets such as our own published and unpublished data that we have included in this response. Thank you again for such a careful and stimulating review – it was fun to consider all of your points and the act of doing so improved the paper and gave all of us lots of really interesting opportunities to think harder about transcription, modern high-throughput experiments and theoretical approaches that answer to such data.

Please address these concerns in particular:

1. demonstrate the novelty of the results vs. previously published work from your lab and others

   Author's response: We were surprised to see that reviewer 2 found that this work was little more than a rehashing or a review of previous work from our or other labs. That is very much on us. We regret that we did not make the arguments and the novelty of the paper more clear. To that end, we have rewritten the Introduction in the hope of being more clear about our objectives, emphasizing why we think they matter and what the reader should take away from this work.

   First, we think of this as a very practical contribution related to experiments being done right now in our labs. One of the most exciting recent experimental developments is the advent of massively parallel reporter assays (MPRAs), which are experiments we have been practicing in very high throughput in our own lab. The data from such experiments literally involves tens of millions of different promoter variants captured in summary statistics such as information footprints and expression shift matrices (the latter being a second useful read-out now included in our revised paper). As shown in our website for the new iteration of Reg-Seq and Appendix R1, we have experimentally produced over 3000 information footprints for the *E. coli* transcriptome under more than 30 different growth conditions (these data are currently unpublished as yet, please don't share). In the high-throughput language of the current state of the art, these results are *literally* from measuring expression in tens of millions of cells. Given such experimental results, we are interested in applying all of the theoretical tools at our disposal to understand that data. One such approach is the use of thermodynamic models, but we are by no means patriotic to only this class of models. The key point is that we view this as a novel, PLOS Computational Biology-worthy paper because it is engaging in a detailed, rigorous way with a

real, huge and complex dataset. Indeed, as we now make clear in our Abstract and Introduction, this paper uses a new version of thermodynamic models to make tens of thousands of separate theoretical promoter variants and to capture the results of those huge synthetic datasets in summary statistics that mirror those used in experiments. We urge the referees to review previous work in the field – we strongly believe that they will find no previous work in which thermodynamic models with base pair specificity were used to generate huge synthetic datasets, nor in which the result of such simulations were used to generate the kinds of summary statistics used in MRPAs. Further, some of these summary statistics such as the expression shift matrices are themselves new to us (not trying to make a big deal out of it, but just saying that even the quantities we choose to compute are often new).

Our first interpretive step is to use these models as a tool to explain real data that exists now, not hypothetically. We are considering a progression of theoretical approaches – thermodynamic models, graph-theoretic approaches, chemical master equations, etc. However, the goals are practical: the question is how we can interpret tens of thousands of information footprints and tens of thousands of expression shift matrices (again, see Appendix R1) in order to make the smartest guesses we can about the underlying regulatory architectures. How can the data from these kinds of experiments be used to reveal the signatures of non-equilibrium effects in transcription? We argue that it is novel and useful to have a "theory of the experiment" in which we can use a well-controlled and well-established model to create synthetic datasets that can then be used as a tool for understanding how to interpret real datasets (for other examples of people generating these kinds of datasets, see the work of Kinney et al. [1], Urtecho et al. [2], and Lagator et al. [3, 4]). Though of course we are interested in whether or not thermodynamic models are "right," the most immediate use of these models is to create a theoretical infrastructure that precisely mimics the real-world experimental pipelines used in MPRAs with the ambition of rigorously interpreting the outcome of such experiments. When we *know* with certainty what went into the synthetic datasets, we can unambiguously interpret them.

Second, while there is existing work on inferring binding energies using thermodynamic models [5, 6], we are unaware of any previous work from us or others in which a base-by-base description of energy is used to predict expression and the resulting information footprints are computed. The closest work we are aware of is Lagator et al. (2022) [3]. In two of our own previous papers [6, 7], we showed how energy matrices could be used to design new promoters and new repressor binding sites (theoretical results that we tested rigorously ourselves), but nowhere have we or others (to the best of our knowledge) done a systematic study of how changing bases in regulatory regions changes gene expression. Note that although we have not pursued this point in the present paper, these base-pair specific thermodynamic models are a very exciting starting point for the analysis of the evolution of gene regulation since they will make it possible to concretely explore the connection between sequence, expression and fitness. Below is a more specific rendering of the novelty question in terms of our figures that we believe offer novel results to the field:

- Figure 3 presents for the first time to our knowledge the expected information footprints and expression shift matrices for all of the key regulatory architectures in *E. coli.* Note that the use of expression shift matrices as a summary statistic is new to us (again, to the best of our knowledge) and no one besides us has used or shown how to calculate these expression shift matrices (or footprints) from massive theoretically generated mutant libraries. The point of this figure is to provide a benchmark for us and others when performing Reg-Seq or other MPRAs so that we have a stronger basis for making our guesswork about where transcription factors bind.

- Figure 4 explores the way that mutation rate alters information footprints. We are unaware of any work that even touches on this point, and once again, this is a jumping off point for trying to design maximally informative experiments, but also begins to provide a

basis for understanding the connection between mutations and the evolutionary change of transcriptional regulation.

- Figure 5 explores a deeply important point. How many mutant sequences do we need in a library in order to distinguish binding sites from noise? Again, this is practically oriented as seen by examining by hand the 3000 information footprints in Appendix R1. Note that this question has hugely important consequences if for no other reason than because the financial cost of the relevant mutant libraries with the aim of doing a genome-wide exploration of regulatory architectures is prohibitively high. The smaller the library, the better.

- Figure 7 explores the tricky question of how the information footprint (or the raw RNA-Seq data itself) depends upon the relative strength of RNAP binding and transcription factor binding. Though the weak promoter approximation allows us to ignore the presence of RNAP in thermodynamic models, real world promoters do not always satisfy the weak-promoter approximation. Again, in the spirit of understanding what can happen in real data, this figure provides a benchmark for how the relative binding strengths influence the information footprints and expression shift matrices. If we and others are going to use these summary statistics as a discovery tool for massive data sets, then we need to know what factors control their quantitative structure. When used in this mode, we have no idea a priori about the copy numbers of the relevant transcription factors nor of the strength with which they bind DNA. We and others need a framework for anticipating what will happen in such experiments.

- Figure 8 does indeed build on seminal work such as that of Buchler et al. [8] and de Ronde et al. [9]. By no means are we claiming "novelty" in that we are the first to consider AND and OR logic etc. Rather, once again, we are trying to deeply understand the relationship between regulatory logic and information footprints and expression shift matrices. Though we are not sure if the reviewers caught it, we find it fascinating that the information footprints are so very different in the AND and OR cases.

- Figure 9 takes a close look at how the number of transcription factor binding sites affects information footprints. This is a very pertinent point in the context of MPRAs and has not yet been thoroughly examined in the literature. On the one hand, many transcription factors regulate the expression of more than one gene; therefore, transcription factor binding site copy number is highly variable and needs to be taken into consideration when we analyze MRPA datasets. On the other hand, from an even more practical perspective, when MPRAs are conducted in bacterial systems and the mutant library is delivered through bacterial transformation, this helps us to choose a plasmid of an appropriate copy number.

- Figure 10 demonstrates the relationship between inducer concentration and MPRA datasets. In their perspective article, Lindsley and Rutter underscores the importance of allosteric regulation in various aspects of biology [10]. In the experimental work conducted in our own lab, we also make sure to perform MPRAs in a diverse range of growth conditions so as to cover various forms of allosteric regulation involved in transcriptional processes. Our work here provides a reference point for what to expect in MPRA outputs when inducers are at work to regulate transcriptional machineries.

- Starting from Figure 12, we begin to explore the difference sources of noise in MPRAs. In the analysis of real world datasets such as ours, it is important that we are able to distinguish between signal and noise. In Figure 12 in particular, we focus on the effect of extrinsic noise. It is true that many papers such as that by Raser and O'Shea, Fu and Pachter, as well as Elowitz et al. have explored extrinsic noise at length [11, 12, 13]. However, our paper contributes by connecting the current literature on extrinsic noise to MPRAs, which is something that we do not think has been explored elsewhere.

- Figure 13 considers whether non-specific binding along the promoter itself may be a significant source of noise in information footprints. In addition, as shown in our revised manuscript, we also directly address the presence of functional binding sites away from the usual transcriptional start sites, which we have seen in our own experimental datasets produced in our lab. To do this, we make use of a novel form of thermodynamic models where each state is built by sliding the RNAP along the promoter.
- Figure 14 provides some examples of how overlapping binding sites may affect the signal in information footprints and expression shift matrices. As shown in databases such as RegulonDB and EcoCyc, overlapping binding sites is commonly seen but it is so far unclear how these overlapping binding events would affect MRPA summary statistics. Our work here tackles precisely this issue.

Finally, of course, we are excited to make all of our data and resources available to everyone. This includes our website for this paper that we are currently continuing to develop. This website serves as a computational tool where the user may select and change different parameters of interest and generate information footprints for themselves. We plan to add more features to the website and provide functionalities for exploring a larger set of parameters and a wider range of regulatory architectures.

2. underscore the limitations of the thermodynamic equilibrium approach

Author's response: We consider this one of the most exciting frontiers of the subject of transcriptional regulation. We are with both reviewers in the sense that we think that thermodynamic models should be relentlessly scrutinized. Our laboratories are deeply engaged in both the theoretical dissection of transcriptional regulation as well as the design of experiments whose objective is precisely to understand how energy consumption and broken detailed balance lead to transcriptional behavior that is not within the purview of the thermodynamic models. The details of our arguments on this very important topic are spelled out in our point-by-point responses below. We are grateful for this discussion and agree that the limits and validity of such models is a centerpiece of current explorations of this subject. We have added an appendix in our Supporting Information that attempts to give a cursory but scholarly survey of the state of the art in thermodynamic models in biology writ large and in the study of transcriptional regulation in particular. This survey attempts to highlight some key successes and failures of the thermodynamic model approach. In the paragraphs below, we summarize our own current and tentative thinking on the subject in the hope that maybe the reviewers and editor will have thoughts of their own to share.

We believe the level of discourse about thermodynamic models in the field could and should be greatly improved. Their implicit use is far more widespread than people acknowledge and often their rejection is based on unsubstantiated philosophical remarks about the inapplicability of equilibrium thinking. We believe a more nuanced discussion in this field would help propel a meaningful theory-experiment dialogue forward. Rather than dismissing such models because "living organisms are not in equilibrium," a more nuanced view would recognize that apparently overly simplistic assumptions such as a gas of non-interacting electrons, or ionization of atoms in stars based on equilibrium thinking (Saha equation), to name a few examples, are often more powerful than we originally think. The applicability of the seemingly naïve thermodynamic models goes well beyond the study of transcription since their use is pervasive for all sorts of molecular reactions in biology. One of the most successful and well documented examples is in the context of chemotaxis where there has been a profound discourse between theory and experiment. We hope the editors and reviewers will agree that we need to be cautious about dismissing work based upon thermodynamic models – though we offer more details below – we

suggest that a careful examination of the ways in which such models have been used to achieve subtle and impressive data collapse in the context of both chemotaxis and quorum sensing indicate that these approaches are often a reasonable and ubiquitous null model.

One aspect of this discussion that we think is important to clarify is that although we are quite excited about alternatives to thermodynamic models and are actively engaged with precisely these questions both experimentally and theoretically, the occupancy probability mindset (of which the thermodynamic models are a particular, specific instance) are way more pervasive than is commonly acknowledged. For example, the entire field of synthetic biology, including the original genetic switch [14] and the repressilator [15], is replete with examples of equations of the form

$$\frac{dA}{dt} = -\gamma A + r\frac{\left(\frac{A}{K}\right)^n}{1+\left(\frac{A}{K}\right)^n}$$

in which A is some protein of interest (we are being very schematic here), γ is the protein degradation rate, r is the transcription rate (which for this case is self-activated), and n is the Hill coefficient. The second term on the right side is an occupancy term that characterizes the probability that the promoter will be occupied by the activator. Though it is not often acknowledged out loud, this is a thermodynamic model of the same variety as those we are using. Many high-profile papers on topics ranging from bacterial sporulation to bifurcations in hematopoiesis all use occupancy thinking [16, 17, 18]. Many examples of such models can be seen in the books by Uri Alon [19] and Markus Covert [20]. The differential equations that are the centerpieces of these books have right-hand sides that are based on the probability of occupancy, often with Hill functions. In fact, as shown in Fig R1(B) and (C), Hill functions are a simplified version of thermodyanmic models in which the probability of certain singly occupied states are arbitrarily simply set to zero. As seen from Fig R1(D), despite our own strong preference for a full states and weights (as opposed to the Hill function), from a phenomenological perspective the differences between them are extremely subtle.

However, the hidden assumption of occupancy reaches much more deeply into the subject including in the context of ChIP-Seq experiments and even sequence gazing itself. We have earlier shown experimentally that there is a total breakdown of the thermodynamic models [21] and we are fans of the work of Johan Elf who has similar examples of the failure of quasi-equilibrium or occupancy probability thinking [22]. By way of contrast, there are also many powerful, careful examples where such models produce results that are in complete accord with experiment [23, 24, 25, 26]. In fact, we would claim that is the rule rather than the exception. To that end, here we provide a long list of examples (and the list could be much longer) where the thermodynamic model approach is either explicitly or implicitly in play. We used this list to help us organize our own thinking on the subject as we wrote our new S1 Appendix in response to the need to be more circumspect about thermodynamic models and we thought it might be of interest to the reviewers and editor.

- A.V. Hill introduced what we now call the Hill function to describe *equilibrium* binding of oxygen to hemoglobin and was explicit about the shortcomings of the model as we say in our appendix since it did not include the states of intermediate occupancy [27, 28].
- The seminal paper by McGhee and von Hippel in 1974 [29] presents an equilibrium model on protein-DNA binding, accounting for both non-interacting and cooperative ligands on a one-dimensional lattice.
- In their 1982 and 1985 papers, Ackers and Shea [30, 31] develop thermodynamic models for the cooperative binding interactions of the bacteriophage lambda repressor with the lambda right operator. The model uses equilibrium binding data to predict repression curves, which

highlights the importance of cooperative interactions between repressor dimers at adjacent operator sites.

- The genetic switch paper by Collins (in particular, Equations 1a and 1b) [32] and the repressilator paper by Elowitz [15] - both of these classics in synthetic biology write down equations of the form given above with the regulation of genes entering through Hill functions that are a thermodynamic model, whether the authors say so explicitly or not.

- Goldbeter is famous for having made a model of circadian rhythms [33]. This work writes down coupled differential equations and as usual, the right hand sides have production terms that are proportional to the probability of different states that are written in the Hill form.

- Eq 1 and Eq 2 of Suël et al. (2006) [34] are other instances of occupancy thinking with the right-hand side of the equations reflecting explicitly the probability of certain states that are obtained using simplified statistical mechanics.

- In the paper by Walczak, Tkacik, and Bialek (2010) [35], there is a whole section on the "regulation function" and the authors explain why Hill functions are a powerful and useful approach (and we illustrate that in Fig R1 by considering a comparison between Hill treatments of occupancy and the full states and weights approach).

- In the beautiful example presented by Sivak and Thomson (2014) [36], stem cell fate is described using an auto-activation switch and, yet again, the underlying mathematical description offers differential equations with the right-hand side given by an occupancy function of the Hill form.

- The paper by Kuhlman et al. (2007) [23] happily goes beyond Hill functions to do a full statistical mechanical model of activation and repression in the *lac* operon. We were requested to discuss the limitations of thermodynamic models, and this paper constitutes a serious, rigorous effort to address that point. The conclusion is largely that the thermodynamic framework is a powerful tool for describing thoughtfully designed experiments.

- Wingreen group both on chemotaxis and quorum sensing. In both of these studies, thermodynamic models were used to systematically and rigorously examine data from the Berg and Sourjik labs (see Figure 5 of Keymer et al. [37]) and the Bassler lab (see Figure 6 of Swem et al. [38]). The signature of the success of these models is the data collapse (figures referred to above). Note that in both cases, the x-axis in these figures is explicitly the free energy. Even earlier, Mello and Tu wrote a thermodynamic model (not using these words, but it doesn't matter what the words are – there are states and weights and partition functions) to describe chemotaxis [39]. Tu in 2008 has a PNAS paper in which he carefully describes the shortcomings of this approach [40].

- Martin and Wall on the *mar* operon [41, 42] is another study cut from the same mold as the study of Kuhlman et al., where the thermodynamic model framework was used carefully to study transcription in this operon.

- Doughty et al. (2024) [43] – this high profile paper from Greenleaf and Bintu does a beautiful single-molecule job of measuring the connection between the occupancy of transcription factors, nucleosomes and the level of expression. As can be seen, their null models are all couched in the language of thermodynamic models. They use those words explicitly, though many of the papers cited throughout this list do not. Regardless, all of them share a focus on calculating the probability of different states of promoter occupancy.

- Though it is not about transcription or occupancy, we want to call attention to the recent paper of Julicher, Hyman and Weber on thermodynamic models (and associated experiments) for studying phase condensates in *C. elegans* [44]. Of special note is a separation of time scales argument about local heating due to ATP hydrolysis. Note also that they have a phase diagram from within a living embryo.

- Mirny et al. (2010) [45] investigates the statistical mechanics of competition and interaction between transcription factors and nucleosomes. Using thermodynamic-model-based thinking, Mirny shows that nucleosome-mediated cooperativity allows flexibility in regulatory regions, which is analogous to the kind of cooperativity in hemoglobin.

- Grigorova et al. (2006) from Carol Gross' group [46] explores transcription regulation by activator recruitment and sigma factor competition using an equilibrium model of RNAP binding.

- Papers by Stormo and colleagues [5, 47] use thermodynamic models to infer binding energies of transcription factors based on expression data. Our work directly adopts this kind of approach and utilizes it in the opposite direction, where we use sequence-specific binding energies in the context of thermodynamic models in order to predict expression levels.

- We could go on with this list. It seems impolite to refer to our own work, but we too have worked hard on rigorously testing thermodynamic models. It is discouraging to have to refer to our own work - the lead corresponding author has made it a policy for his entire career to *never* tell someone else to cite his work. But again, the tenor of this discussion seems wide of the mark given the extreme subtlety of the parameter free data collapse of the data from Brewster et al. [26] that is provided in Figure 4 of Weinert et al. (2014) [48]. This is a stringent and demanding summary of years of data obtained using single-cell and population methods, enzymatic assays and fluorescence measurements, etc. and is based on the thermodynamic framework. Similarly, in Figure 7(B) of Razo et al. in Cell Systems [25] we show another surprisingly robust data collapse of a huge number of experimental situations where genes have been induced, inspired by the work of Wingreen et al. referred to above. Garcia's paper in 2011 [24] kicked off our own attempts to carefully examine these systems with a theory-experiment dialogue in which specific parameters such as transcription factor copy number and DNA binding site strength were tuned explicitly to test the models. In some ways, this work is just a "mopping up" operation on the earlier amazing work of Müller-Hill and Oehler. We reiterate, we are not partisans who are interested in defending thermodynamic models – we will be perfectly content to discover when they are found wanting. But having spent more than a decade in this and other fields using such models, the act of exposing their shortcomings is not as easy as it sounds and cannot be based upon philosophical objections to equilibrium thinking in biology.

- We are also aware of the other literature on the failure mode of thermodynamic models. Kreamer et al. (2005) [49] shows a miserable failure of trying to use the thermodynamic framework in the context of *P. aeruginosa*. That said, it is really not clear if it is the model which fails or simply incomplete knowledge. We are now currently engaged in using information footprints to try and finally sort this mess out.

- Garcia et al. (2012) [21] reports another really interesting failure mode of the thermodynamic models which, like the Kreamer paper, includes confusion surrounding the issue of binding site position.

- Eck et al. (2020) [50] critiques the MWC models in the context of allosteric regulation. Their study focuses on the regulatory dynamics of the hunchback gene by activator Bicoid and transcription factor Zelda in Drosophila embryos and demonstrated that no thermodynamic model could recapitulate hunchback transcription.

- Another of our favorite papers that shows an apparent breakdown of occupancy thinking is in the context of the glucocorticoid receptor [51]. Occupancy thinking leads to the expectation that stronger operators will more strongly affect gene expression and this paper defies that expectation.

We want to emphasize again, we are not partisans that are interested in defending thermodynamic models. It is confusing to be having this debate when there is such a huge literature on the use of these models and because of the fact that although we use these models regularly, we are actually trying very hard to rigorously come up with a coherent, careful picture of the general rules of transcriptional regulation. Indeed, in our laboratories, the subject of greatest interest is when equilibrium models do *not* work. We are working constantly to understand how energy investment alters signaling and regulation. On the other hand, we believe that as a field we should be clear about our null models and the list above should make it very clear that occupancy thinking is ubiquitous. Null models should not be a subject of philosophical debate. Instead, they should be debated on the basis of a scholarly, rigorous, quantitative dialogue between theory and experiment. Our current favorite unifying framework is that by Jeremy Gunawardena [52] which has the breadth to encompass equilibrium models as the limit of the more general broken detailed balance framework.

This is all to say: a) we make no claims that thermodynamic models are definitively the way to go and we have rewritten the introduction to make it entirely clear that there is still work to be done to understand the limits of validity of these approaches, b) we are actively engaged in developing more general approaches with concrete predictive power in the spirit of Jeremy Gunawardena [52], c) we find that the thermodynamic model framework provides us a powerful platform for having gold standards where we know the answer going in so that we can test our thinking on information footprints and expression shift matrices, and d) we believe the story of models of transcription need more rigorous, careful work and less verbal assertions either about the success or shortcomings of such approaches. Finally, as noted above, we added an appendix to the paper that attempts to explain our own sense of the limits and validity of thermodynamic models.

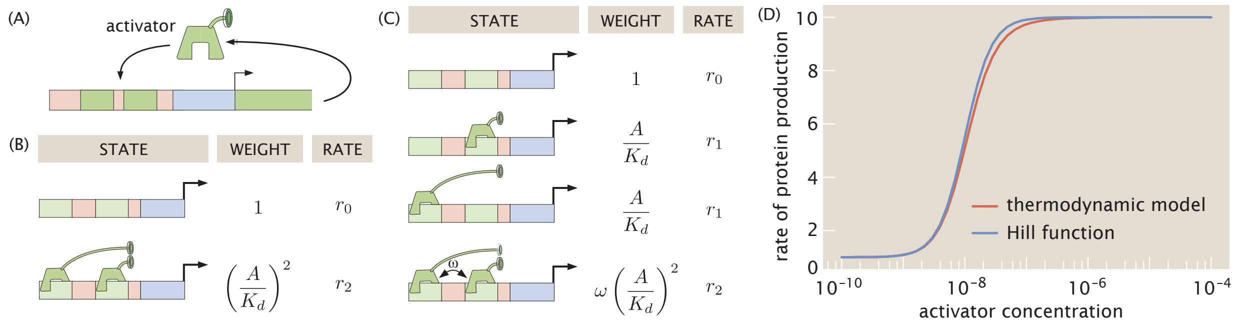

Figure R1: **Comparison between the Hill function and a full thermodynamic model for an auto-activation switch.** (A) Architecture of the auto-activation gene circuit. The activator can bind on its own promoter such that when there is sufficient level of activator present, the level of transcription will be enhanced relative to its basal value. (B) States, weights and rates for the auto-activation switch when binding is described by a Hill function. (C) States, weights and rates for the self-activated promoter described by a thermodynamic model. ω represents the cooperative binding energy between the two activators. (D) Comparison of the rates of activator production as a function of activator concentration in the thermodynamic model and the Hill function.

Reviewer 1 comments

Pan et al. have attempted to develop a theory for massively parallel reporter assays (MPRAs) that tries to delineate the relationship between DNA sequence and phenotype. In particular, the authors used the so-called "thermodynamic model" of gene regulation to develop a pipeline to characterize how the different biological and experimental parameters control measured MPRA data. These parameters include the transcription factor (TF) binding site copy number, limited TF resource, etc. These biophysical parameters dictate the degree of mutual dependence between mutations in the regulatory region and expression levels. The authors systematically characterized the effects of various parameters on MPRA data. Moreover, authors showed how to optimise MPRA experimental designs.

The paper is very clearly written. The approach of "developing a theory of the experiment" is particularly important in biology. The paper should definitely be accepted. I have the following comments that the authors can try to address.

Main points

1. A figure describing the model and a brief introduction to the statistical ensemble / distinct microstates involved would be nice for people unfamiliar with the background literature. This can go to the methods or the supplementary section.

   > **Author's response:** We agree with the reviewer that an introduction to thermodynamic models will be useful to some of our readers. This section has been included in the S2 Appendix of the Supporting Information, which includes a figure on how we conceptualize and write down thermodynamic models (included here as Fig R2). We thank the reviewer for the suggestion.

2. I wonder if the assumption of equilibrium is universally applicable to analyse MPRA data. Is it possible to extend the model to consider the scenario where the detailed balance condition is broken for TF binding to regulatory DNA.

   > **Author's response:** This is a fantastic point and something that we are constantly and actively engaged in thinking about. As noted above, one of our primary objectives in this work is as a "theory of the experiment" that will help us to interpret the results from MPRAs. As such, the use of thermodynamic models allows us to create a synthetic dataset which we can use in turn to see how well our algorithms for discerning regulatory architectures work. As can be seen from the list of references that we provided in our general comments above, vast amount of previous works from both our group and others have demonstrated that the equilibrium assumption is often powerful and appropriate in many systems. Therefore, within the closed analysis of synthetic datasets and information footprints, we believe thermodynamic models provide a great platform for better understanding the nature of real experimental data.
   >
   > Nevertheless, as we emphasized earlier, we are not defenders of thermodynamic models and in fact, we very much doubt they will work in all circumstances. Indeed, part of the goal of this work is to figure out how to recognize the breakdown of equilibrium thinking in MPRAs. We are fully aware that the equilibrium assumption may not be universally applicable. For instance, as mentioned in the same list of references in the general comments, Kreamer et al. [49] attempted to use equilibrium thermodynamic models to predict the binding strength of regulatory element BqsR in *P. aeruginosa*. However, the results cannot be reconciled with experimental measurements, which may be explained by the invalidity of the equilibrium assumption in *P. aeruginosa*, though that remains an open question.

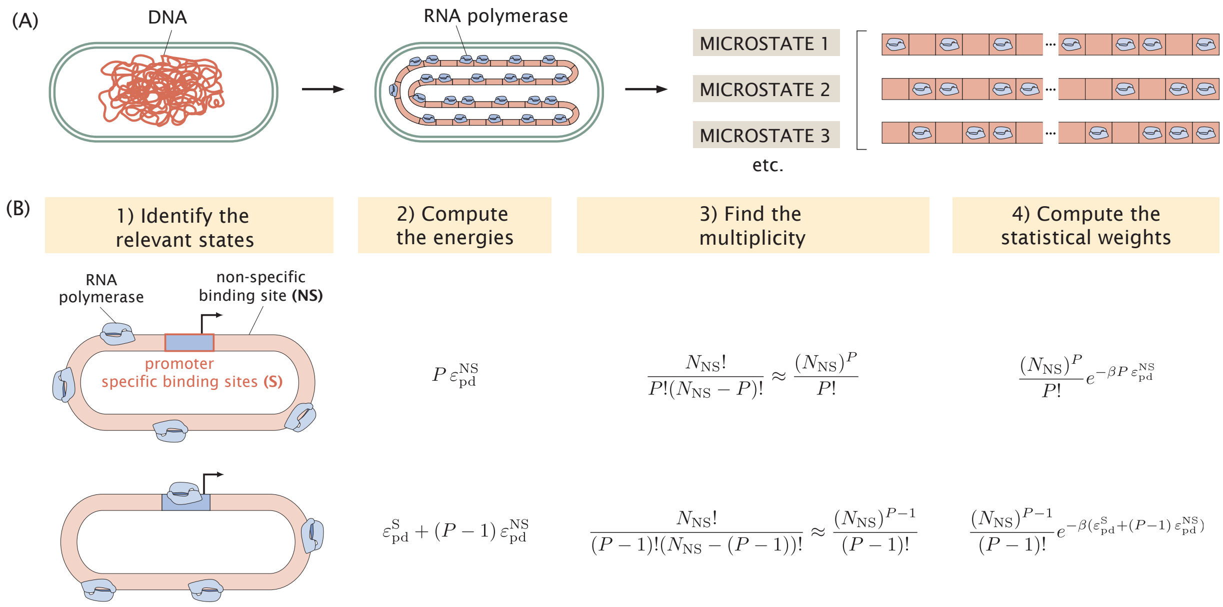

Figure R2: **Writing down thermodynamic models for transcriptional regulation.** (A) Conceptualizing binding events along the genome as microstates. Each of the red boxes is a site along the genome to which RNAP and transcription factors can bind. Each of the possible configurations of binding is considered a microstate. (B) Protocol for writing down a thermodynamic model. The protocol involves four important steps. Firstly, all the relevant promoter states need to be identified. Secondly, the energies of each state are computed. Next, we compute the multiplicity, which tells us the number of possible configurations associated with each state. Finally, statistical weights are written down based on the energies and the multiplicity terms. For the energy terms ε, the superscript NS refers to non-specific binding, the superscript S refers to specific binding, the subscript pd refers to RNAP (p) binding to DNA (d).

To address the limitations of the thermodynamic models, one of our favorite approaches – which subsumes the thermodynamic models as a special case – is the use of graph theory, where we can work out the steady-state probabilities when detailed balance is broken. This approach is originally introduced by Gunawardena [52], and Mahdavi, Salmon et al. from our group have used the approach in the context of transcriptional regulation in their manuscript, which is currently in revision and available on bioRxiv [53]. We are excited to extend the analysis in the original version of the paper and we have included an example of what the information footprints will look like in the cases where detailed balance is broken. Specifically, we can use the graph-theoretic model to calculate the probability of the transcriptionally active states, which can then be used to predict the expression levels of promoter variants and allow us to build synthetic datasets with broken detailed balance.

To see how this can be done, let us consider a promoter with the simple activation regulatory architecture. Following the approach presented by Mahdavi, Salmon et al. [53], the regulatory architecture may be described using a directed square graph with four vertices and eight edges, as shown in Fig R3(A) (which is now also included in the main text as Fig 15). Each of the vertices represents one of the four possible promoter states: empty (E), bound by RNAP (P), bound by the activator (A), or bound by both RNAP and the activator (AP). As explained in our main text, we can use the Matrix Tree Theorem to write down the statistical weights of each state as a function of the rate constants. We can then calculate the probability of the transcriptionally active states, $p_{\text{active}} = p_{\text{P}} + p_{\text{AP}}$, by summing up the weights of the RNAP-bound state and the

activator-RNAP-bound state and dividing by the total weights of all four states.

The challenge here now lies in how to preserve sequence-dependence in p_{active}. One possible way to achieve this is to first estimate the rate constants $k_{\text{on}}^{\text{eq}}$ and $k_{\text{off}}^{\text{eq}}$ at equilibrium for each binding event. As a starting point, we assume that binding is diffusion limited and therefore $k_{\text{on}} = k_{\text{on}}^{\text{eq}}$ is constant. On the other hand, given the dissociation constant K_d, we have that

$$k_{\text{off}}^{\text{eq}} = k_{\text{on}}^{\text{eq}} \times K_d.$$

In particular, K_d can be written as

$$K_d = c_0 e^{\beta \Delta \varepsilon},$$

where $c_0 = 1\,M$ is the reference concentration of the standard state and $\Delta\varepsilon$ is the binding energy of the transcription factor at its binding site, which can be calculated using energy matrices. As described in our paper, energy matrices map binding site sequence to energy terms with base-pair specificity. As a result, this confers sequence-specificity to K_d and therefore $k_{\text{off}}^{\text{eq}}$. Finally, we can estimate k_{off} at non-equilibrium by taking into consideration the energy U invested to break detailed balance, where

$$k_{\text{off}} = k_{\text{off}}^{\text{eq}} \times e^{\beta U}. \tag{1}$$

We can then use k_{on} and k_{off} for each edge to calculate p_{active} and predict the expression levels of mutant promoters without imposing equilibrium constraints.

As shown in Fig R3(C), panel 2), using the graph-theoretic approach to produce synthetic datasets at equilibrium produces an information footprint that is qualitatively similar to the footprint produced using thermodynamic models. Moreover, when we break detailed balance at the edge from the activator dissociates from the state where the activator and RNAP are both bound, we observe interesting behaviour in the relationship between mutations and expression levels. For example, as shown in Fig R3(C), panel 4), in the cases where a positive energy is invested to break detailed balance and when activator concentration is high, the binding of activator displaces the binding of RNAP. As a result, the activator effectively behaves as a repressor. This result is consistent with what has been reported by Mahdavi, Salmon et al. [53]. Given these results, we believe the non-equilibrium approach outlined above shows great promise. We are very excited to explore this further in a future paper and we appreciate the reviewers' interest in our future efforts on this front.

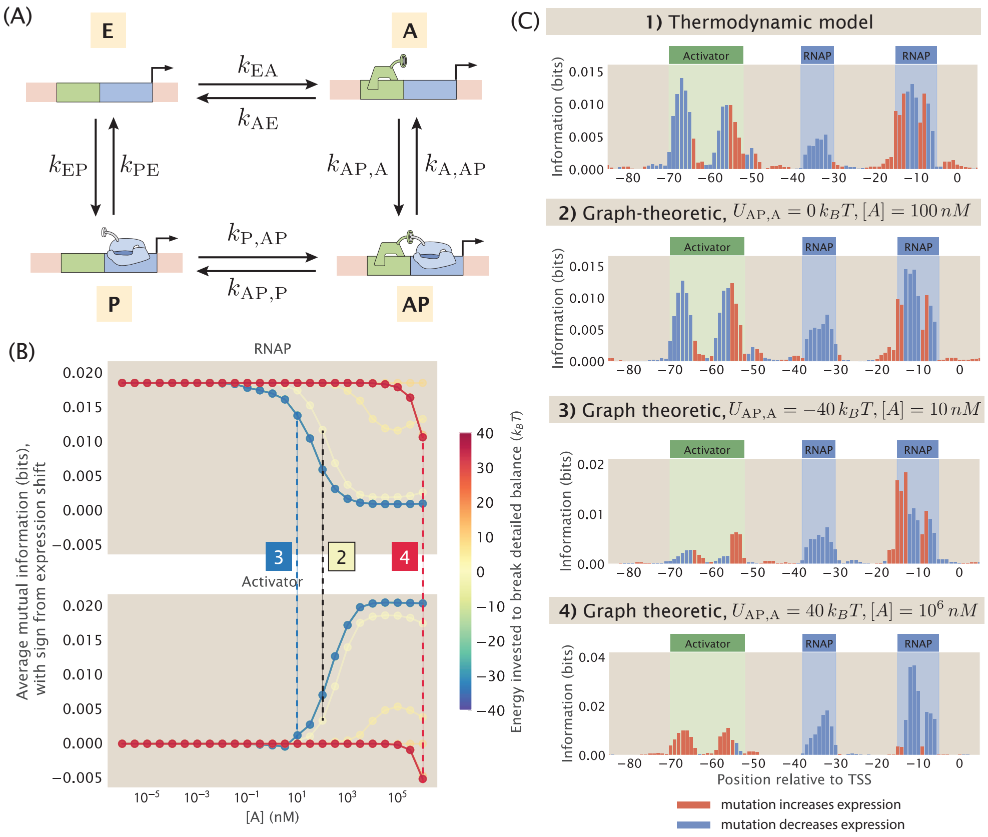

Figure R3: **Building synthetic datasets with broken detailed balance.** (A) Directed square graph that describes the kinetic processes of a simple activation promoter. (B) Changes in average mutual information at the RNAP and activator binding sites when the concentration of the activator ($[A]$) and the energy invested to break the detailed balance at the AP $\rightarrow$ A edge ($U_{\mathrm{AP,A}}$) are changed. The sign of the value on the y-axis is based on the expression shift values calculated using Eq 9 in the main text. The three numbered labels point to datapoints for which we show the corresponding information footprints in (C). (C) Information footprints built using the thermodynamic model and the graph-theoretic model. The second footprint is a kinetic/graph-theoretic treatment of the equilibrium case.

Minor Points

1. What is N_s? In line 79 it is referred to as the number of non-binding sites, in 82 it is referred to as the total number of base pairs in the genome.
2. The reference in line 76 (1) should be (17).
3. In line 322, it is mentioned that when R1 > R2, the signal at the R1 binding site is higher but in the figure 8C 3) it is lower. I think the authors mean to say R2.
4. In Figure 8C, subfigures 5) and 4) have the same captions.

Author's response: Regarding the first point, N_{NS} is the total number of non-specific binding sites. In our analysis, since the number of binding sites is on a much smaller order of magnitude, we assume that the number of non-binding sites is approximately equal to the number of base pairs in the genome.

Points 2-4 have been corrected. We thank the reviewers for pointing out these errors.

Reviewer 2 comments

Pan et al. present a theoretical study to infer the function of regulatory sites in promoters from data from massively parallel reporter assays (MPRA) using the so-called thermodynamic equilibrium model of gene regulation. The paper is clearly written and the results all seem valid (apart from some small errors that we noticed, as detailed below) and I do not have much to object to the contents or the interpretations of the results provided. However, two main points temper my enthusiasm regarding this work:

1. The corresponding author has published a whole host of papers already on the thermodynamic equilibrium model approach to transcription regulation (in particular for prokaryotes) including papers on the various effects explored here (e.g. the titration effect paper of Brewster et al) as well as on the use of this approach in the context of MPRA (e.g. in collaboration with Justin Kinney). Given this existing literature, I struggled to identify what is really novel here. I first thought that maybe an integrated computational tool would be presented but it does not look like that to me. It also was not obvious to me what qualitatively new effects were being explored, or how the theory presented goes beyond what is already in the literature. I guess the paper could maybe be of value as an introduction to the thermodynamic model of prokaryotic gene regulation and interpretation of MPRA data for students that are starting in this field. But this is maybe a bit thin for a paper in PLoS Comp Biol. I would suggest that the authors revise the paper so as to focus on the aspects that they consider novel and bring these out more clearly.

Author's response: We apologize for being unclear about the objectives of the paper and what it has accomplished. It is a bad sign that the reviewer struggled to figure out what was new. Indeed, we have written two *experimental* papers on the Sort-Seq and Reg-Seq approaches to regulatory dissection and as will be clear from the our Reg-Seq website and Appendix R1 with over 3000 information footprints, we are now trying to get this strategy to work genome-wide so we can decipher the regulatory architectures for more or less every gene in *E. coli.* We ask that the reviewer visit the website for our experimental MRPA pipeline where we show more than 3000 distinct information footprints. In themselves, these footprints are not interesting. They are interesting to the extent they shed light on regulatory architectures of the kind shown in Figure 4 of Ireland et al. [54]. The current paper is quite distinct and the way we think of it is as a concrete, precise theory of the experiment in which we can better understand what dictates the features of both information footprints and expression shift matrices so that we are better able to use those treatments of the data to infer actionable regulatory architectures. In this sense, nearly everything in this paper is new.

This paper does build on prior work on building equilibrium thermodynamic models of transcriptional regulation. However, our previous work has not used thermodynamic models with the statistical mechanical energy matrices for calculating sequence-specific binding energies $\Delta\varepsilon_{\text{S}}$. This now allows us to take any mutational distribution we want and to compute the level of gene expression. Despite the reviewer's assertion that we have done such work before, neither we nor anyone else (to the best of our knowledge) has done anything like this. Essentially, we have used the thermodynamic models thousands of times here to compute thousands of "single-cell" expression values and we capture those results in experimentally accessible summary statistics of information footprints and expression shift matrices. We are confident that not only will this allow us to better understand MPRA experiments, but it will also make it possible to have a more realistic treatment of the evolutionary implications of mutations that goes beyond the very cool models in Gerland and Hwa [55], Lynch and Hagner [56], and Berg et al. [57] In those earlier works, their energy matrices treated all mutations as equivalent and assigned them an equal energy penalty ε. Our use of thermodynamic models (or rate-based models that are described in one of our earlier points and included in the revised manuscript) that are sequence-dependent allows us to ask a much broader array of questions.

Concretely, in response to the unfortunate confusions we caused for the reviewer:

(a) We have provided the computational tool suggested by the reviewer and will continue to expand both the experimental and computational parts of the website so that it can be used to complement the qualitative information in RegulonDB.

(b) We have rewritten the abstract and introduction to make it clear that this is a new generation of base-pair specific thermodynamic models in which the simplified specific ε_S and ε_{NS} binding energies are replaced by sequence-specific binding energies.

2. I do not object to the thermodynamic equilibrium modeling that is used here, but I think it should be made clear that this is an extremely simplified model of what actually happens inside prokaryotic cells and that we currently do not know whether it is actually generally a quantitatively accurate model. Cells are typically growing and expanding exponentially, their DNA is replicated (which interferes with transcription), gene copy numbers vary as the DNA is replicated (and there has been recent evidence that this drives variations in expression at the single cell level), levels of TFs may be fluctuating on time scales that are not necessarily slow compared to the time scales of transcription, translation and dilution of proteins (which invalidates the thermodynamic model's assumptions). There are all kinds of non-equilibrium effects in the process of transcription and translation (eg. just the fact that RNA polymerase leaving the promoter when it starts transcribing is an irreversible non-equilibrium process, but also the super-coiling resulting from transcription, just to give two arbitrary examples). Although I agree that the equilibrium model that assumes transcription rate is proportional to equilibrium polymerase occupancy is a good starting model, we actually have no idea yet of how valid this model is generally for quantitatively describing gene regulation in prokaryotes. Given this, I am more than a little annoyed at the really grandiose tone in this manuscript, starting from the title. There is virtually zero acknowledgment in the manuscript that this model is a highly simplified model that may well not be appropriate in many situations. This needs to change. The authors need to point out how strong the simplifications are that are being made and that this is just a reasonable starting model and that we do not know yet whether it is generally quantitatively accurate.

Author's response: This part of the reviewer's response makes it clear how far we fell short in communicating the point of our paper and also in apparently conveying some blind faith in thermodynamic models (which we do not have at all). The whole point of this current project is based upon a complete dissatisfaction that we apparently share with the reviewer about the state of the art in transcription. At the risk of being repetitive, the starting point of our work is the observation that for more than 60% of the genes in *E. coli.* As shown in Fig R4, we literally know nothing about how those genes are regulated. As part of our efforts to overcome that ignorance, we have an ambitious and successful experimental program in using MPRAs in conjunction with information theory to infer regulatory architectures for previously unannotated promoters and then the use of mass spectrometry to identify the transcription factors that bind those putative binding sites. We have already performed these experiments on over a hundred promoters in more than 30 growth conditions, meaning that we have over 3000 distinct information footprints and expression shift matrices to consider (again, please see our experimental Reg-Seq website and Appendix R1, where we present over 3000 information footprints that we have generated for the *E. coli* transcriptome under more than 30 different growth conditions.) The present work is aimed at that analysis in the context of *E. coli* with no claim that it has the grandiose implications that the reviewer attributed to us.

At the same time, though we share the reviewer's cautious outlook on the role of thermodynamic models, we find it to be inconsistent with the literature to make assertions about having "no idea" whether such models work. We feel the need to push back on that claim, especially in light of the use of thermodynamic models with way more success than failure in the past three decades.

Beginning with the classic work of people such as von Hippel [58], Ackers and Shea [30, 31], Mark Ptashne [59], Ann Hochschild [60], Carol Gross [46], Kathy Matthews [61], Bob Schleif [62, 63, 64], Gary Stormo [5, 47] and many more from the previous great generation of biologists, huge quantifiable progress has been made. The classic papers on simple repression and DNA looping from Müller-Hill [65, 66] and Oehler [67, 68] were so far ahead of their time and led to careful, rigorous theoretical work based on thermodynamic models such as that of the beautiful results of Vilar and Leibler [69], Vilar and Saiz [70, 71], Kuhlman and Hwa [23]. Moreover, the thermodynamic framework is often the go-to approach of many, many groups other than our own as evidenced by the nearly 1000 citations of the Bintu et al. papers [72, 73] that attempted to present the fundamentals of these models in a pedagogical way. Given the achievements of so many people, we feel that such a strong dismissal (without any literature citation) is unwarranted. As we offered earlier in our response, we have tried to give a careful and systematic analysis of the successes and failures of thermodynamic models both in our revised Introduction and S1 Appendix of our Supporting Information. We apologize for giving the impression of grandiosity, and we have changed our title to "Deciphering regulatory architectures from synthetic single-cell expression patterns" in order to be more specific about the aims of this paper.

Finally, as noted in the response to reviewer 1, we are extremely enthusiastic about approaches that make no quasi-equilibrium assumptions and have written a long paper that examines the implications of broken detailed balance [53]. As discussed in our new Results section on "Builiding synthetic datasets under non-equilibrium conditions", the graph-theoretic approach used by Mahdavi, Salmon et al. [53] shows strong promise to be incorporated into our current pipeline. That said, the signature of non-equilibrium effects in the context of MPRAs is not entirely transparent. As part of the pleasure of responding to this review, we interviewed Yuhai Tu, Jeremy Gunawardena and Jordan Horowitz and two of the three of them argued that we will likely require dynamical information (not present in the MPRA approach) to unequivocally detect non-equilibrium effects. We remain unsure about this point. We are actively seeking experimental signatures of the breakdown of thermodynamic models such as those exhibited in the work of the laboratory of Johan Elf [22] or papers from Yuhai Tu in the chemotaxis setting [40, 74, 75]. We are excited to further pursue this direction in a future paper.

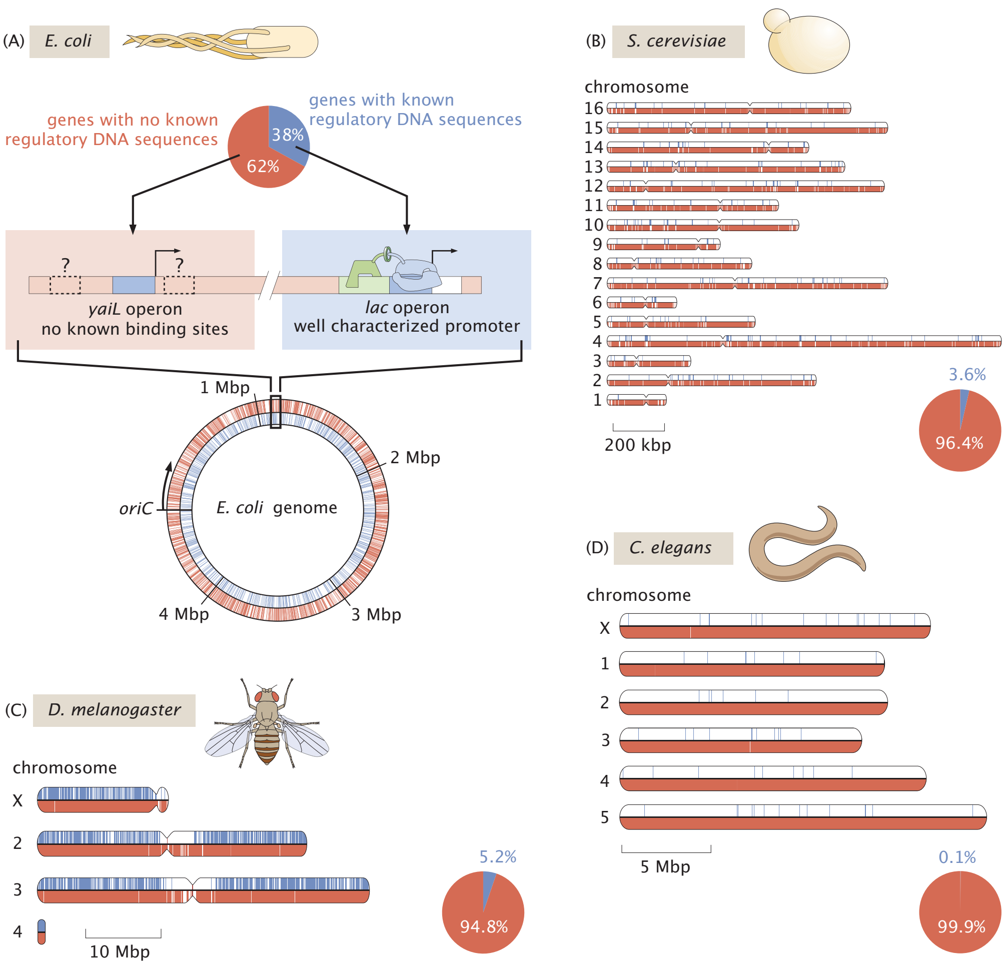

Figure R4: **Regulatory ignorance in key model organisms.** (A) Regulatory ignorance in the *E. coli* genome. According to current databases (as of 2016), there are 872 operons for which we have some knowledge of their regulatory architecture (shown in blue), while there are 1762 operons for which we currently have no regulatory knowledge (shown in red). (B) Regulatory ignorance in the *Saccharomyces cerevisiae* genome. Roughly 96% of the genes have no regulatory annotation. (C) Regulatory ignorance in the *Drosophila melanogaster* genome. Roughly 95% of the genes have no regulatory annotation. (D) Regulatory ignorance in the *Caenorhabditis elegans* genome. Over 99% of the genes have no regulatory annotation.

I have a few more minor concerns which mainly consider cases where the authors seem to be drawing stronger and more general conclusions than I think can be supported by what is shown, and also some things that were unclear:

1. What are the "states" i in equations (3) and (4)? Are these different σ_{70} binding sites? (or maybe also alternative sigma factor sites). And what is the mechanistic interpretation of having different rates r_i at these different sites?

> **Author's response:** We apologize that this was not more clearly explained in the original version of our paper. Here, i are the indices of the transcriptionally active states. For example, for a promoter with the simple activation regulatory architecture, there are two states that are transcriptionally active, one is where only RNAP is bound and one is where both RNAP and the activator are bound. It is conceivable that the rates of transcription are different for these two states. Therefore, the average rate of mRNA production is the weighted average of the mRNA

production for each of the two states. We have clarified this further in the main text of the paper.

2. Lines 131-135. I understand that these simplifications (only two bins and only mutation vs. wild- type) will generally increase mutual information, but they obviously also lose information in that we can no longer distinguish the effects of different letters nor distinguish small from large changes in expression. So I do not think this simplification is always 'optimal'. This warrants more discussion.

Author's response: We realize that there is a misunderstanding here, our choice to calculate mutual information by putting expression levels in two bins and coarse-graining base identities does not increase mutual information. In fact, reducing the number of bins in general leads to a lower mutual information, as can be seen when a comparison is made between the top and bottom information footprints in Fig S3(C) (originally Fig S1(C)). However, as we have discussed in S4 Appendix of our Supporting Information, the bin reduction does lead to a higher signal-to-noise ratio, which is crucial for identifying regulatory architectures for unannotated promoters. We note in passing for the reviewer's curiosity (and in the hope that maybe they will think about this point), in the original Kinney work and in the recent work from Tkacik, more bins were used. We have worked hard on this point in both Sort-Seq and Reg-Seq and believe this is quite a subtle point and the idea that "more bins is better" seems to be wide of the mark. One of the intriguing ways that we pursued this experimentally was to grab a bin of cells and resort. Anyway, this last point is *not* offered as part of our "response." Rather, it is just something that has been in the back of our minds for years that maybe the reviewer has some insights about.

The goal of this work is building a tool to help us to decipher regulatory architectures in the context of MPRAs. We are aware that we may lose some information on how different mutations at each position contribute to the subtle changes in expression levels. However, as mentioned in our earlier responses, the second summary statistics of MPRA datasets, namely expression shift matrices, are very useful in giving us base-pair resolution information on how mutations alter gene expression. Expression shift matrices show both the sign and magnitude of the change in expression levels for all possible mutations at all positions. Specifically, the value in the expression shift matrix at position l corresponding to base b is given by

$$\Delta s_{b,l} = \begin{cases} \dfrac{1}{n}\displaystyle\sum_{i=1}^{n} \xi_{b,i,l}\left(\dfrac{c_i}{\langle c\rangle} - 1\right), \text{ where } \langle c\rangle = \dfrac{1}{n}\displaystyle\sum_{i=1}^{n} c_i, & \text{if } b \text{ is the mutated base identity} \\ 0, & \text{if } b \text{ is the wild-type base identity} \end{cases} \tag{2}$$

where $\xi_{b,i,l} = 1$ if the base at position l in the i-th promoter variant corresponds to base identity b and $\xi_{b,i,l} = 0$ otherwise. We were remiss in the previous submission in not including this metric. We now present expression shift matrices in the context of our computational pipeline and for different regulatory architectures in Fig 1 and Fig 3 of the main text (also shown in Fig R5 and R6). Again, we note that both information footprints and expression shift matrices are summary statistics from thousands of individual cells (both experimentally and theoretically). As summary statistics, they are not a complete representation of the sequence-expression linkage. This work is precisely designed to understand such summary statistics.

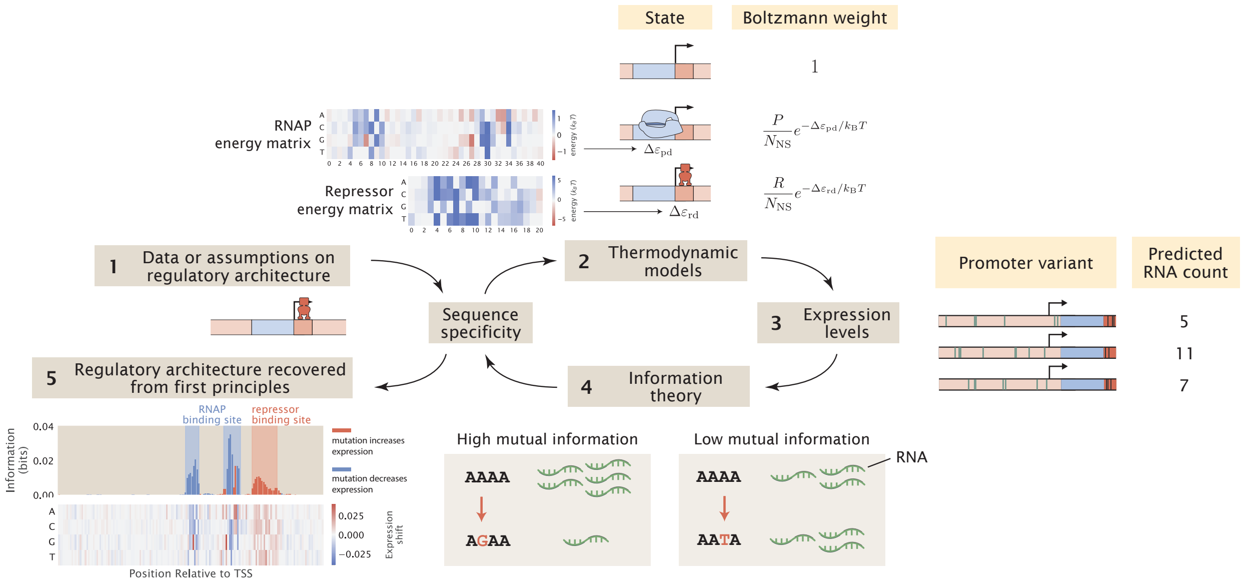

Figure R5: **Revised Figure 1 on the computational pipeline for deciphering regulatory architectures from first principles.** An example of an expression shift matrix has been added to panel 5.

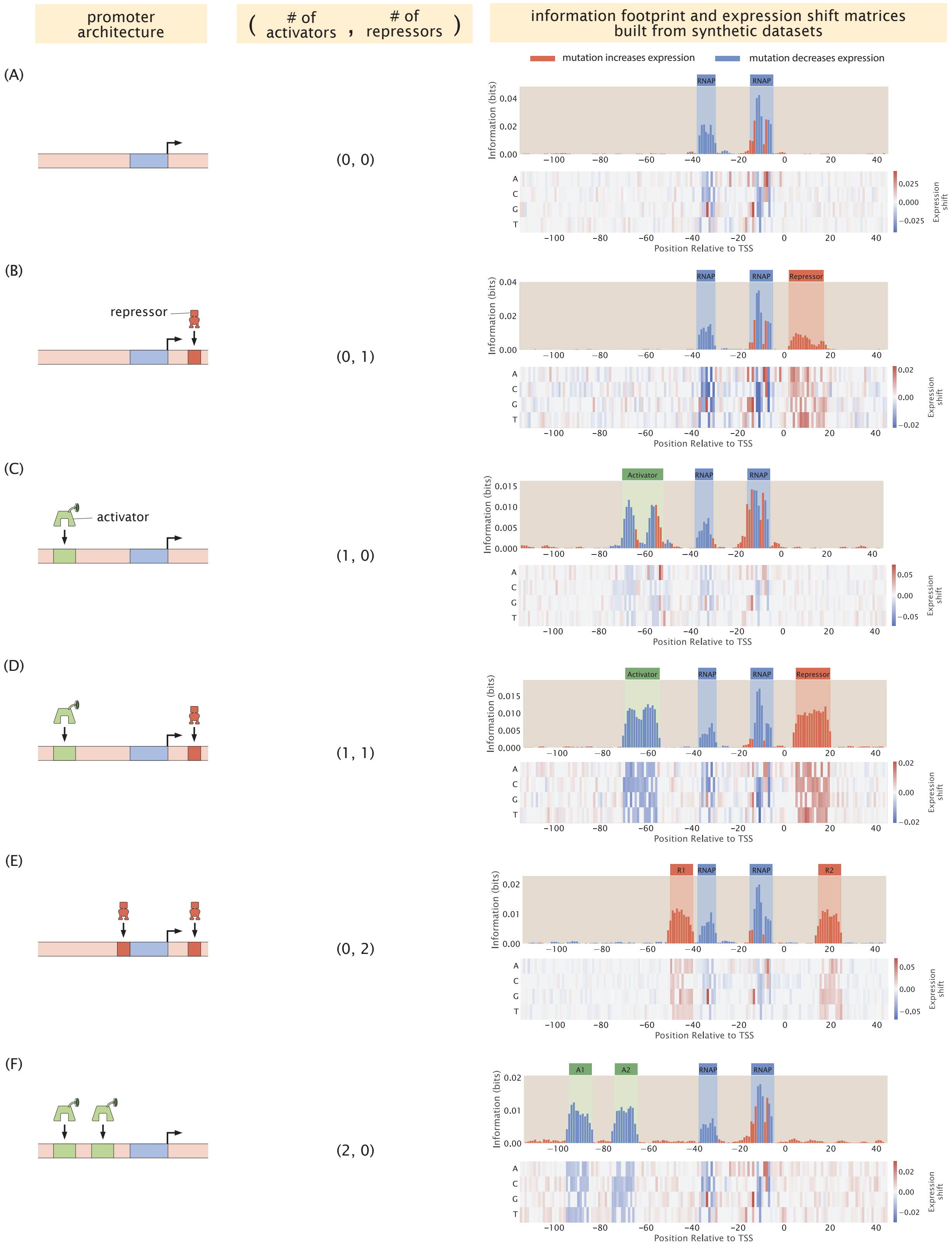

Figure R6: **Revised Figure 3 on building information footprints and expression shift matrices based on synthetic datasets of different regulatory architectures.** For each of the regulatory architecture, we added an expression shift matrix that reflects the expected binding sites.

3. AND and OR models for function of promoters with two repressor sites. There should be more discussion that these strict AND and OR functions are an idealizations (which may not be anywhere close to how repressor sites actually interact in real promoters) and more generally, that we do not really know how to model the interactions between regulatory sites if there are more sites per promoter (i.e. how the transcription rate depends on the precise binding configuration of the TFs). There are plenty of promoters in E. coli which can have 5 or more binding sites and multiple sigma sites. It would be good for the authors to acknowledge that it is not obvious how the methods they present can be extended to such more complex situations.

Author's response: We are a little confused by the reviewer's point. For a given hypothesised architecture, though there is a combinatorial explosion of states, we and everyone else who uses statistical mechanical models know how to write down all the states and their statistical weights. Perhaps the reviewer is pointing out that the harder problem of determining the rate $\langle r \rangle = \sum r_i p_i$ summed over all transcriptionally active states involves many unknown parameters. We certainly agree, and with the combinatorial explosion, the more transcription factors that bind, the more undetermined parameters. As we have said elsewhere in the response, one of the main points of this work is to explore exactly such questions since we control the parameters and can see what response they elicit.

Moreover, while it is true that some promoters have annotations of 5 or more transcription factor binding sites on databases such as RegulonDB and EcoCyc, these annotations are aggregated over all growth conditions that have been studied. In reality, when we examine transcription factor binding for each promoter under a single growth condition, most genes in *E. coli* are regulated by no more than two transcription factors during transcription initiation. As shown in Fig R7, which is adapted from Mahdavi, Salmon et al. [53], over 80% of the promoters are regulated by fewer than two transcription factors. Nevertheless, we are in agreement with the reviewer that it is a limitation of our pipeline that we do not consider the rare promoters that are regulated by more than two transcription factors. We have addressed this point in the fourth paragraph of our Discussion section.

On the other hand, if the reviewer's concern is that AND and OR logic gates may not represent the full spectrum of interactions between transcription factors, then we do wholeheartedly agree. However, as discussed by Buchler et al. [8] and de Ronde et al. [9], only a limited number of logic gates are attainable through parameter variations, and a thermodynamic model can be written down for each of the possible logic gates. This means that our thermodynamic-model-based computational pipeline can be easily adapted to consider all possible types of interactions between transcription factors.

To give an example of how this can be done, let us consider the exclusive-or (XOR) logic gate in a promoter regulated by two repressors, which is another important and interesting logic gate other than the AND and OR gates. As illustrated in Fig R8(A), under the XOR gate, gene expression is repressed when only one of the repressors is present at high concentrations, but not when both of the repressors are present at high concentrations. One possible mechanism by which this may occur is if the interaction between the two repressors is repulsive. As shown in Fig R8(B) and R8(C), when the copy number of the second repressor is kept constant at 25 and the copy number of the first repressor is increased from 0 to 50, the signal at the first repression binding site increases and the signal at the second repressor binding site decreases. This behaviour is consistent with the definition of XOR logic gates and further demonstrates that our computational pipeline can handle a diverse range of interaction regimes. We have now included the example of XOR gate in the S8 Appendix of our Supporting Information.

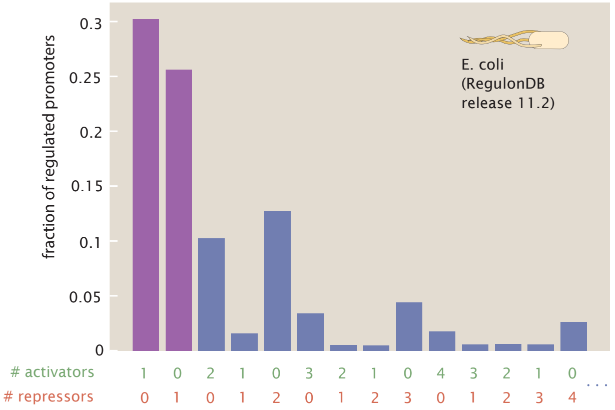

Figure R7: **Distribution of regulatory architectures in *E. coli* based on census from RegulonDB.** The figure is adapted from Mahdavi, Salmon et al [53]. Note that we worry that databases such as RegulonDB are incomplete since they do not say how growth conditions alter which transcription factors are bound. Our experimental and theoretical work aims to clarify this.

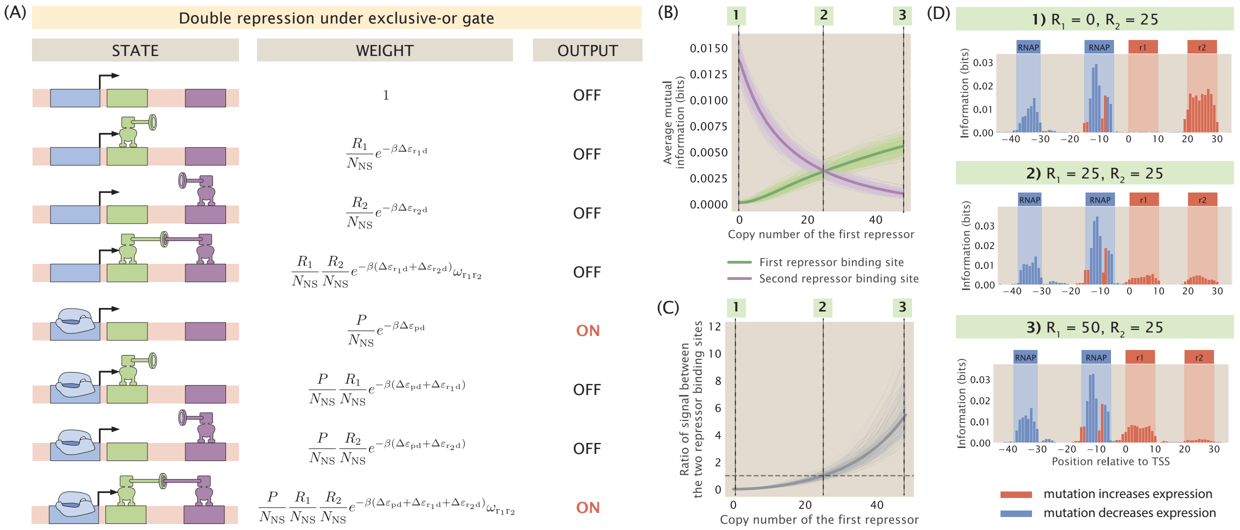

Figure R8: **Changing repressor copy number for a double-repression promoter.** (A) States-and-weights diagram of a promoter with the double repression regulatory architecture and under the exclusive-or (XOR) gate. (B) Changes in the average mutual information at the two repressor binding sites when the copy number of the first repressor is increased. For the energy matrices of the repressors, the interaction energy between the repressor and a site is set to $0\,k_BT$ if the site has the wild type base identity and set to $1\,k_BT$ if the site has the mutant base identity. To enforce the XOR logic, the interaction energy between the repressors is set to $5\,k_BT$. 200 synthetic datasets are simulated and the trajectory for each of the synthetic dataset is shown as an individual light green or light purple curve. The average trajectories across all 200 synthetic datasets are shown as the bolded green curves and the bolded purple curves. The three numbered labels correspond to the information footprints shown in (D). (C) Ratio of average mutual information between the two repressor binding sites when the copy number of the first repressor changes. The individual trajectories (plotted in light grey) and mean trajectory (plotted in dark grey) are from the same 200 synthetic datasets used in generate the plot in (B). As expected, the ratio is equal to 1 when the copy number of the first repressor is equal to the copy number of the second repressor. (D) Representative information footprints with three different combination of repressor copy numbers.

4. In equation (15) the authors derive an 'optimal' mutation rate by taking the parameters of the LacI site and σ_{70} site at the lac promoter and working out a mutation rate that puts these sites on equal footing. But the expression for kappa (equation (12)) depends on several parameters that can be very different at different binding sites and promoters and it is not even guaranteed that there is a mutation rate that makes the expected effect of mutations on different sites equal. I therefore find it quite misleading that this is presented as if it were some general optimization procedure.

Author's response: If we understand the reviewer correctly, we are guilty of creating a misunderstanding. The word "optimal" is used here in a very specific context. We want to ask the question: for a given set of copy numbers and binding site strengths, what choice of mutation rate will give us the most signal for both the RNAP and transcription factor binding sites within the context of the summary statistics used to describe MPRAs? We are not talking about "optimality" in any way that people use that word in the literature. For example, a mutation rate would not be optimal for us when performing MPRA experiments when we only see strong signals at the RNAP binding sites and not at the transcription factor binding site, or if we only see a strong signal at the transcription factor binding site and not at the RNAP binding site. We have also clarified this in the main text (Section 1.2) of our revised paper.

That said, we completely agree that there are many parameters at play in the derivation of optimal mutation rate. To examine the general applicability of our proposed optimization procedure, where we find the mutation rate that yields equal Boltzmann weights for the RNAP and the transcription factor, we have calculated the optimal mutation rate while changing RNAP and transcription factor copy numbers as well as RNAP and transcription factor binding energies. In particular, we are keen to explore the parameter space that honors known copy numbers. As seen in Fig R9, the proteomic study from Li et al. [76] shows that transcription factor copy numbers in *E. coli* ranges from 1 to 10^4 copies per cell. Schmidt et al. [77] also similarly reports that the distribution of transcription copy numbers is from 10 to 10^4 copies per cell. In Fig R9, we have fully explored the effects of transcription factor copy numbers on mutation rate within this physiologically relevant range. Additionally, as shown in Table 1 of Grigorova et al. [46], the copy number of RNAP is typically on the order of magnitude of 10^3 to 10^4. The range of RNAP copy numbers that we selected in our investigation also covers this physiological range.

As shown in Fig R10 (also shown in Fig S4 of our new S7 Appendix in the Supporting Information), we have varied these parameters two at a time, and we observe that increasing the free energy of the transcription factor increases the optimal mutation rate and increasing the free energy of the RNAP decreases the optimal mutation rate. Importantly, we find that within physiological ranges of copy numbers and binding energies, the mutation rates stay within 0.50, which is a reasonable range in the context of MPRA mutant library design.

Again, we would like to note that we are performing this investigation as a "theory of the experiment", and this is done with the very practical purpose of informing experimental design in MPRAs. Here, we are simply trying to understand how parameters should be controlled when we design mutant libraries for MPRAs. The MPRAs as we are using them are a discovery tool. Going in, for an unannotated promoter, we do not know anything about copy numbers, binding site strengths, or even what transcription factors are involved. As a result, we have to make guesses when we design a mutant library and order an oligo pool. These calculations are intended to give us a sense of how to best approach this design. Even though we use the word "optimal" from the real experimental perspective, since we do not know copy numbers of the various factors nor their binding site strengths, this study of "optimality" helps us make better guesses. We know of no a priori way to design best libraries for our experiments and we hope the reviewer understands that library design matters because we are talking about tens of thousands of dollars.

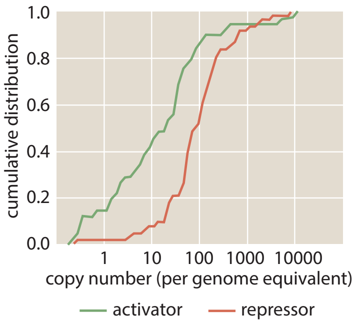

Figure R9: **Cumulative distribution of transcription factor copy numbers in *E. coli*.** Cumulative distribution of the copy numbers of both activators and repressors. The figure is adapted from Li et al. [76].

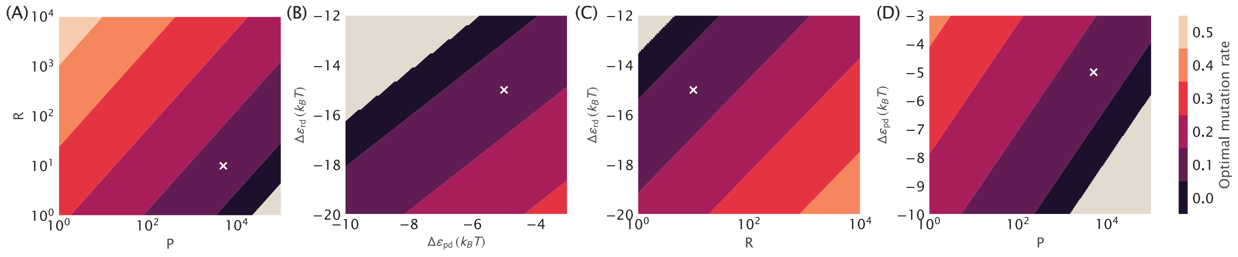

Figure R10: **Optimal mutation rate for single transcription factor binding.** Numerical solutions for the mutation rate calculated by finding the mutation rate that leads to equal Boltzmann weights between the RNAP bound state and the transcription factor bound state using Eq **??**. The white crosses mark the standard set of parameters: $R = 10$, $P = 5000$, $\Delta\varepsilon_{\rm rd} = -15\,k_{\rm B}T$ and $\Delta\varepsilon_{\rm pd} = -15\,k_{\rm B}T$. Panels (A) to (D) vary 2 of the 4 parameters, while the other two stay constant. Gray color indicates regimes where no mutation rate can be found that fulfills the criteria of $\kappa = 1$.

5. Figure 8B, bottom panel. I do not understand that the curves do not intersect at 25 copies because in that case the situation is completely symmetric and both repressors should have the same mutual information. This makes me doubt this plot is correct.

Author's response: We are very grateful to the reviewer for this important observation. We agree that given the symmetry of the architecture, we should expect approximately equal mutual information when the copy numbers of the two repressors are the same and so the two curves should intersect at 25. After some investigation and troubleshooting, we found that the reason behind the non-intersection is that the footprints generated under this set of conditions are more variable than usual. This high variability can be seen from Fig R11. Here, we re-simulated 200 synthetic datasets for the OR-logic double repression promoter. We calculated the ratio of the average mutual information between the two repressor binding sites and plotted this ratio against the copy number of the first repressor for each of the 200 synthetic datasets. Even though the average ratio is approximately 1 when the copy number of the two repressors are the same, we see that this ratio changes significantly from simulation to simulation and can go to as high as 1.2 and as low as 0.8. In the original version of this figure, the curves show the average results across only 20 synthetic datasets – we believe this low sample size coupled by the high variability caused the two average curves to not intersect.

To address this issue, we have revised Fig 8 in the main text (included here as Fig R12) and now plot the trajectories of 200 synthetic datasets as well as the average trajectories. By increasing

the number of synthetic datasets to 10 times higher than what was in the original manuscript, we see that the variability issue is resolved and the two average curves now intersect when the copy number of the first repressor is 25. We have pointed out in our main text the high variability across the 200 trajectories. Finally, we would like to assure the reviewer that we have gone through our other figures and we do not find variability to be an issue in any of our other plots.

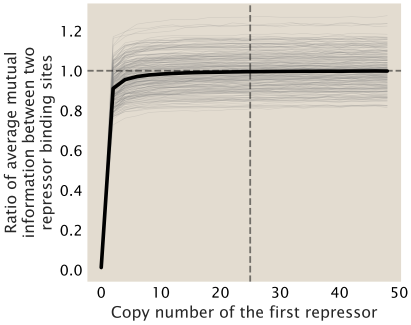

Figure R11: **Ratio of average mutual information between two repressor binding sites for the OR-logic double repression promoter.** For each value of R_1 copy number, 200 synthetic datasets are simulated for a double repression promoter under OR logic and the ratio between the signal at the two repressor binding sites are calculated. The trajectory for each of the synthetic dataset is shown as an individual grey curve and the average trajectory is shown as the black curve.

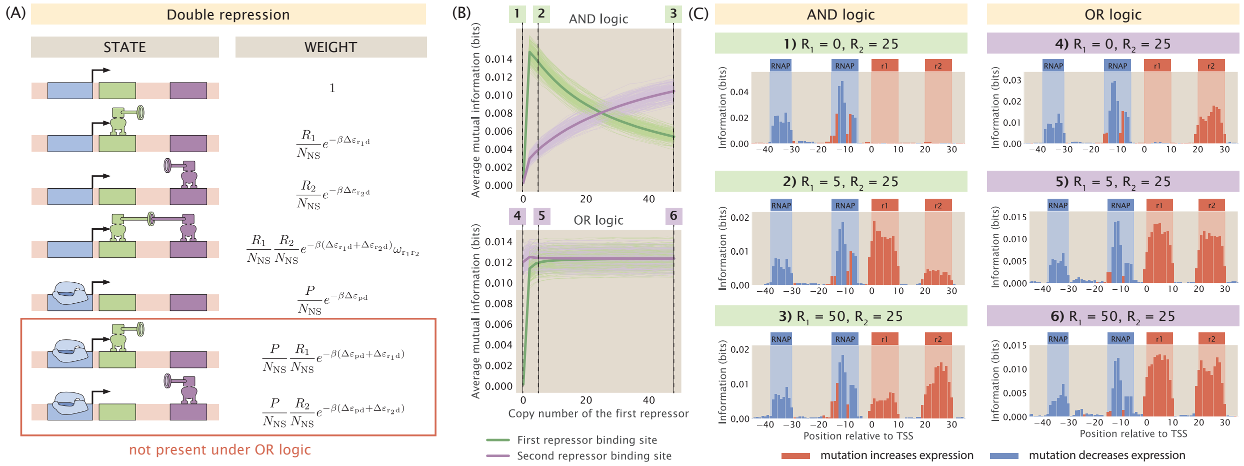

Figure R12: **Revised Fig 8 on changing repressor copy number for a double-repression promoter.** (A) States-and-weights diagram of a promoter with the double repression regulatory architecture. The bottom two states are only present under AND logic and not present under OR logic. (B) Changing the copy number of the first repressor under AND logic and OR logic affects the signal at both repressor binding sites. For the energy matrices of the repressors, the interaction energy between the repressor and a site is set to 0 k_BT if the site has the wild-type base identity and set to 1 k_BT if the site has the mutant base identity. The interaction energy between the repressors is set to -5 k_BT. 200 synthetic datasets are simulated and the trajectory for each of the synthetic dataset is shown as an individual light green or light purple curve. The average trajectories across all 200 synthetic datasets are shown as the bolded green curves and the bolded purple curves. The numbered labels correspond to footprints in (C). (C) Representative information footprints of a double repression promoter under AND and OR logic.

6. Section 3.1. I understand that for the particular example studied here (i.e. the Lac promoter once again) the extrinsic noise did not have a large effect on the signal-to-noise, but that cannot generally be the case. If there are TF binding sites whose effects are weak enough and the noise is large enough, surely at some point the noise will become larger than the signal.

Author's response: We thank the reviewer for raising this interesting and practical point. Like the reviewer, we want to know the conditions under which the summary statistic of the information footprint loses it efficacy. We are trying to use this analysis to understand real world data in order to formulate regulatory hypotheses. Therefore, we have tried to only focus on levels of copy number fluctuations that are physiologically relevant.

Assuming that the extrinsic noise in copy numbers primarily comes from asymmetrical partitioning during cell division, one method to measure fluctuations in transcription factor copy numbers between cells is the dilution method developed by Rosenfeld, Young et al. in the labs of Elowitz and Alon [78]. As seen in Fig R13, based on Brewster et al. [26] who utilized the dilution method, transcription factor copy numbers typically vary by less than 20% of the mean copy number. Additionally, we consider the proteomic measurements from Schmidt et al. [77] and Balakrishnan et al. [79]. In these cases, the coefficient of variation for transcription factor copy numbers is less than 2 even across very different growth conditions. These numbers are not hypothetical. They are the result of careful measurements.

With these empirical data, we can then define our distributions of copy numbers to respect the known levels of fluctuation. In our section on extrinsic noise, we use a Log-Normal distribution to model the copy numbers of RNAPs and transcription factors in order to avoid negative values. As explained in S12 Appendix of our Supporting Information, let X be the copy number of the RNAP or the transcription factor that we are modelling, we define the Log-Normal distribution as

$$\log X \sim \text{Normal}\left(\log \mu, (\alpha \log \mu)^2\right). \tag{3}$$

From this and the known mean and variance of a Log-Normal distribution, we can derive that the coefficient of variation is given by

$$\text{CoV}(X) = \frac{\mathbb{E}(X)}{\text{Var}X} = \sqrt{e^{(\alpha \log \mu)^2} - 1}.$$

Rearranging this expression, we can write down α in terms of μ and $\text{CoV}(X)$, where

$$\alpha = \frac{\sqrt{\log\left[\text{CoV}(X)^2 + 1\right]}}{\log \mu}$$

This means that we can derive a Log-Normal distribution that obeys the empirical mean and coefficient of variation for transcription factors.

As shown in Fig R14(A), we simulated noisy synthetic datasets using Log-Normal distributions with $P = 5000$ and $R = 100$ as the mean copy numbers and a range of coefficients of variation from 0.1 to 10^2. In particular, as shown in Fig R14(B), we examine the footprints with the following three coefficients of variation:

- 0.2, which is based on the Brewster et al. dilution assay [26];
- 2, which is based on the Schmidt et al. proteomic study that covers different growth condition [77];
- 10^2, an unphysiologically high level of coefficient of variation.

We see that while the signal-to-noise ratio decreases as the coefficient of variation increases, signal and noise are easily distinguishable from each other even when the coefficient of variation is unrealistically high.

We agree with the reviewer that in the unlikely scenario of huge noise, our ability to decipher the meaning of information footprints and expression shift matrices would be compromised both in the theoretical models considered here, and more importantly, in the case where we are trying to make inferences based upon real data. However, as mentioned in an earlier comment and shown in Fig R9, proteomic studies by Schmidt et al. [77], Li et al. [76] and Grigorova et al. [46] report that the copy number of transcription factor copy numbers in *E. coli* is between 1 and 10^5 and the copy number of RNAPs is between 10^3 and 10^4. As shown in Fig R15, when the coefficient of variation is set to 100, the copy number of RNAPs can reach as high as 10^9 and the copy numbers of repressors can reach as high as 10^7, both of which are very unphysiological. Again, as shown in Fig R14(B) panel (3), even at this level of copy number fluctuation, the noise in the footprint is low. As a result, we suspect that noise effects beyond these levels in the footprints would be exceptional.

Fig R14 is now included in the main text of the manuscript in place of the original Fig 12 and Fig R15 is now included in the Supporting Appendix in place of the original Fig S8. Note that in the original manuscript, we had a plot of signal-to-noise ratio against "coefficient of variation", but the coefficient of variation on the x-axis there is actually the value of α that we discussed above. The figure is now changed and plots signal-to-noise ratio against the actual coefficient of variation of the Log-Normal distribution since this number is more interpretable.

On the other hand, the reviewer raises an important point that when the signal from binding events is sufficiently low, even low levels of noise may affect our interpretation of information footprints and expression shift matrices. In Sections 2.1 to 2.4 of our paper, we address the cases where the signal may be lowered under certain combination of biological parameters such as transcription factor binding energy and binding site copy numbers. We have now added a new plot in S13 Appendix of our Supporting Information (also shown in Fig R16) that specifically references the section where the signal is lowered during weak repressor binding in order to acknowledge this potential issue. However, even when the signal is low and the coefficient of variation is set to as high as 100, we can still identify signal at the repressor binding site. Therefore, it seems that this is only an issue under unrealistically high levels of noise. For now, we hypothesize that in these cases, the footprints will be too noisy to use to infer regulatory architectures.

Finally, we appreciate the reviewer's concern that our investigation of noise using the Lac promoter under the simple repression regulatory architecture may not be generalizable. To address this concern, we have tested the cases where there are copy number fluctuations under the other four common regulatory architectures (simple activation, double repression, double activation, and repression-activation). As shown in Fig R17, the noise in the footprints remains low regardless of the architecture even when copy numbers are allowed to fluctuate to 10 times above or below the average copy number. We have included this result in the S14 Appendix of our Supporting Information. Thank you for all of these suggestions.

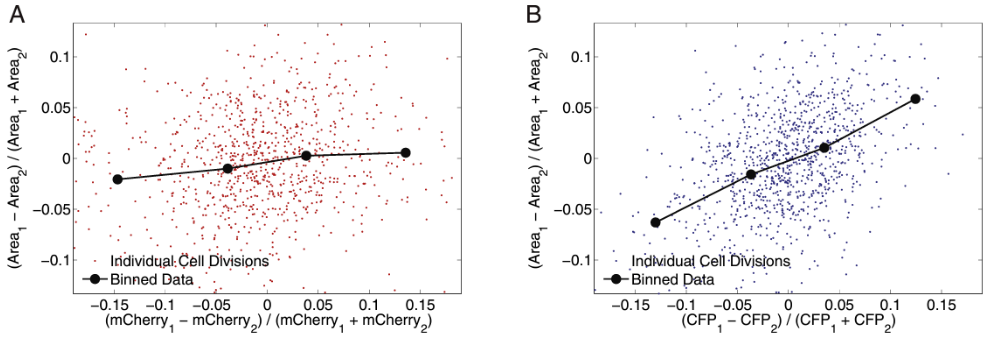

Figure R13: **Dilution effects during asymmetrical cell division measured by Brewster et al. [26].** The figure is taken from Fig S3 of Brewster et al. (A) and (B) show the change in fluorescence levels of LacI-mCherry and CDF between pairs of daughter cells upon cell division.

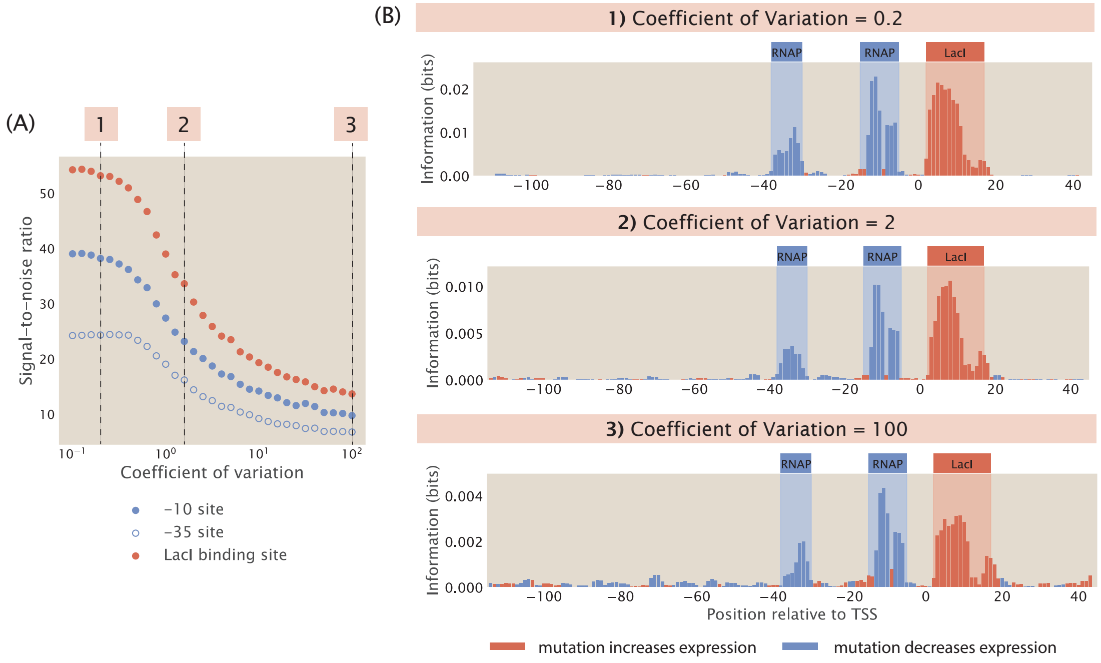

Figure R14: **Revised Fig 12 on Adding extrinsic noise to synthetic datasets.** (A) Increasing extrinsic noise lowers the signal-to-noise ratio in information footprints. For all synthetic datasets used to generate the data points, the copy numbers of RNAP and repressors are drawn using the distribution in Eq 3. In the Log-Normal distribution, μ is set to 5000 for RNAPs and 100 for repressors. Numbered labels correspond to footprints in (B). (B) Representative information footprints with three levels of extrinsic noise.

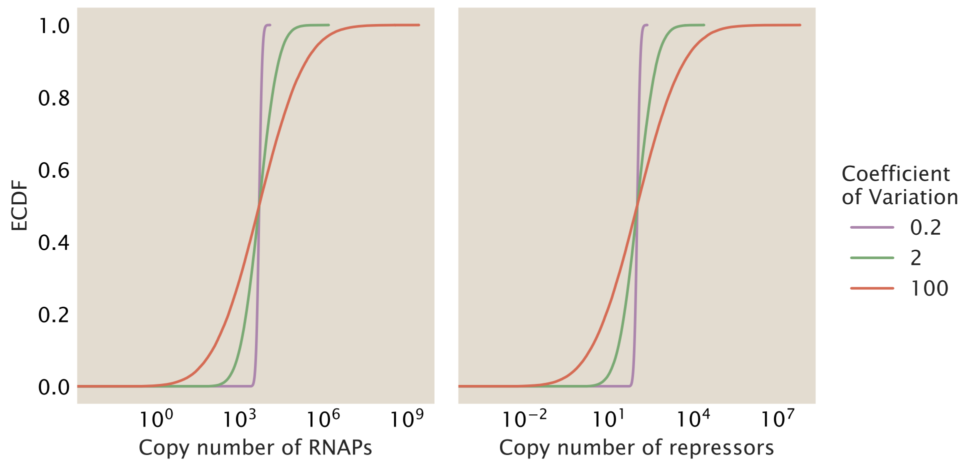

Figure R15: **Distribution of RNAP and repressor copy numbers under different levels of extrinsic noise.** CDFs for the copy numbers of RNAP and repressors modelled using a Log-Normal distribution and under three different levels of coefficient of variation.

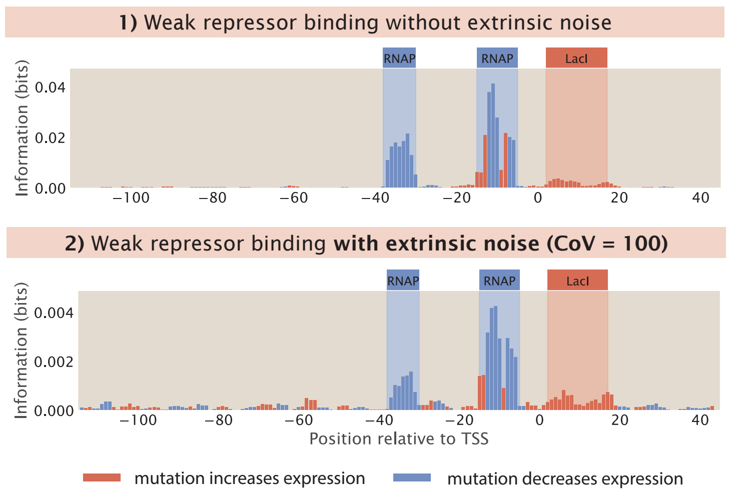

Figure R16: **Effects of extrinsic noise on information footprints with weak repressor binding.** The repressor binding energy $\Delta\varepsilon = -11\,k_BT$ for both footprints. In the top footprint, no extrinsic noise is introduced. In the bottom information footprint, the Log-Normal distribution from which RNAP and repressor copy numbers are drawn has a coefficient of variation of 100.

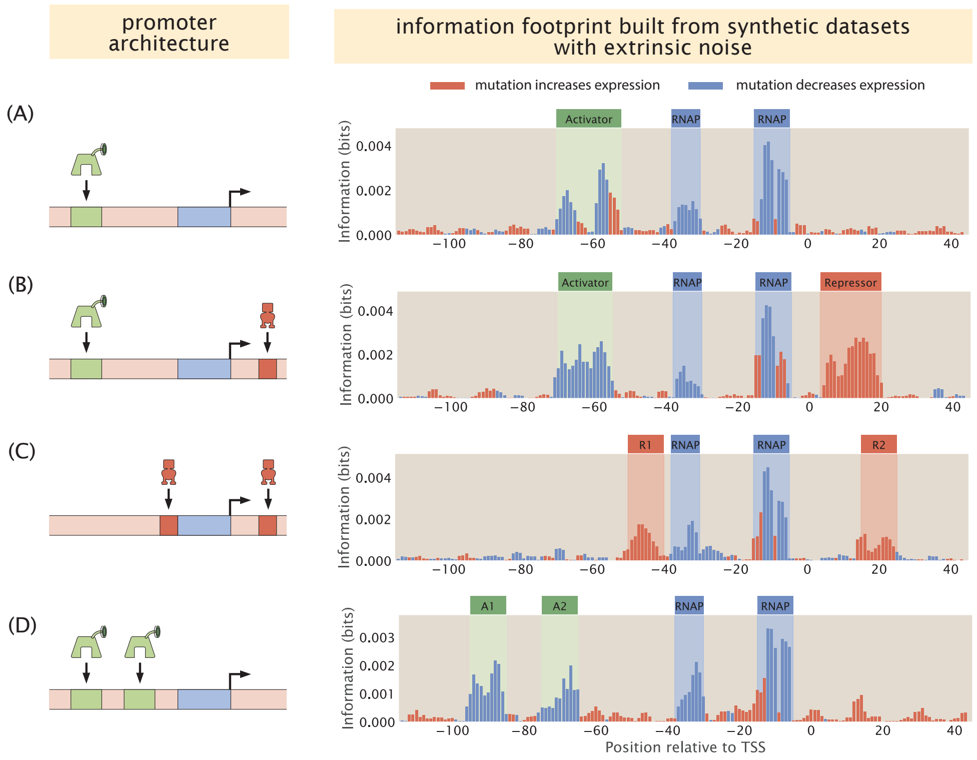

Figure R17: **Information footprints built from synthetic datasets with extrinsic noise under common regulatory architectures.** In all four plots, the coefficient of variation for RNAP and transcription copy numbers is set to 10. Panel (A) to (D) show the footprints from the simple activation architecture, repression-activation architecture, double repression architecture, and the double activation architecture, respectively.

7. Section 3.2. A similar comment applies here. Not every promoter has a clear dominant sigma site and in some cases it might be hard to even clearly distinguish 'spurious' from 'functional' binding.

Author's response: We thank the reviewer for raising this important issue and we share the reviewer's cautionary tone. This has alerted us to the fact that our current use of the word "spurious" may be misleading. The point of Section 3.2 is to challenge the thermodynamic models that we have used in the earlier sections of the paper where we only allow RNAP to bind at a designated binding site, which in this case is the canonical -10 and -35 binding sites along the promoter. To do this, we relax the model and allow RNAP to slide across the promoter so that it can bind at any position. This new model is illustrated through the states and weights diagram in Fig 13(A) of the main text (also shown in Fig R18(A)). By sliding the energy matrix for RNAP across the promoter in one-base-pair increments, we generate a distribution of non-specific binding energies, some of which are stronger than others and will generate non-zero mutual information. The reason for this investigation is to better understand the features of our real data like that shown in the original Reg-Seq website by Ireland et al., our new iteration of Reg-Seq website, and in Appendix R1. The interpretation of such data is always complicated by the presence of noise and the calculations here are to better understand the noise that comes from non-specific binding.

We realize that it may be confusing to refer to off-site binding events as spurious. To address this issue, we have revised our language and now simply call these binding events as non-specific binding events along the promoter. On the other hand, we define functional binding sites as

strong, specific binding sites that lead to a consistent production of mRNA transcripts. These functional binding sites may include the canonical σ binding sites, as well as non-canonical functional binding sites where a mutation leads to strong binding at an otherwise non-specific binding site.

For the latter category of functional binding sites, we reference the study by Yona et al. [80], where the authors have shown that in *E. coli*, there are cases where only one mutation is needed to convert a random sequence into a functional promoter. This short mutational distance between a random sequence and a functional promoter implies that there is a non-negligible likelihood for binding sites to be found at random positions along the promoter. We include these cases as functional binding sites. Indeed, we observe this phenomenon in the data from the Reg-Seq experimental pipeline in our lab. For example, as shown in the information footprint for the araB promoter in Fig R19(A), under growth conditions where arabinose is not present, a strong peak occurs around the -80 position. When mapped to the expression shift matrix shown in Fig R19(B), we observe that this is mainly caused by a point mutation that creates a TATA-like motif at that position. This suggests that a new functional binding site has indeed been created as a result of mutations. We have included a new footprint in Fig 13 of our main text (also shown in Fig R18(B)) where we simulated the scenario where this type of non-canonical functional binding occurs. In particular, we have engineered the promoter sequence such that there is a TATA-like motif upstream of the canonical binding sites. A mutation may easily convert such a site to a functional binding site, creating strong signal in the information footprint.

Given the above definitions, we do expect functional binding sites to be positionally specific and distinguishable from non-specific binding sites. As discussed by Vvedenskaya et al [81], the transcription start site (TSS) for each promoter is generally highly specific. Hence, in the case of canonical σ binding sites, we do expect them to be found in highly specific positions along the promoter. On the other hand, a mutation can only create a functional binding sites under specific sequence contexts. For example, a TATA-like motif can only be created through mutations when the wild-type sequence already has a short mutational distance from the TATA-box sequence. As a result, we also only expect to find non-canonical binding sites at specific positions where the required sequence contexts are available.

Finally, we acknowledge that in real world data, the line between functional binding and non-specific binding may be blurrier than what we have defined above. However, we would like to note one of the main points of this paper is to prepare us to better understand the complexity of real world data. Specifically, the goal of Section 3.2 is to see what non-specific and functional binding events may look like in the summary statistics of MRPAs. Our hope is that our results can serve as a reference point and aid us – and others – in developing regulatory hypotheses when real experimental data is being analyzed.

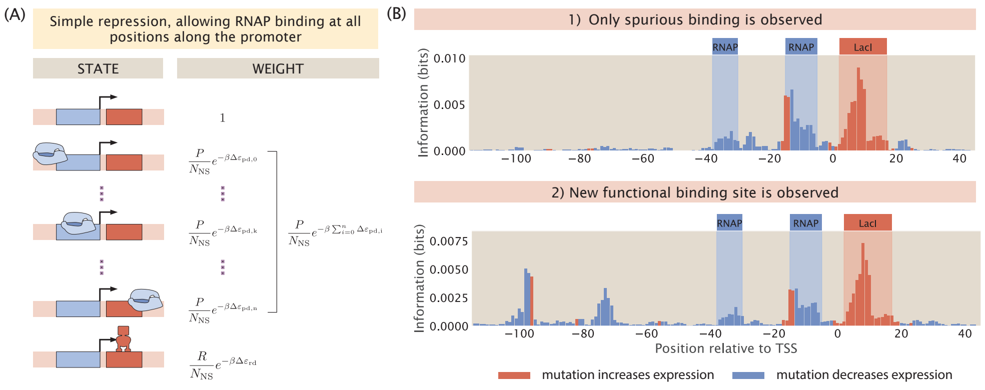

Figure R18: **Revised Fig 13 on off-site RNAP binding.** (A) States-and-weights diagram of a simple repression promoter where off-site RNAP binding is allowed. The index i correspond to the position of the first base pair to which RNAP binds along the promoter. 0 is at the start of the promoter sequence; k is at the canonical RNAP binding site; $n = 160 - l_p$ is index of the most downstream binding site where the promoter is assumed to be 160 base-pair long and l_p is the length of the RNAP binding site. (B) Information footprints of a promoter under the simple repression regulatory architecture with non-specific binding (top) and with a new functional binding site (bottom). The bottom plot is created by inserting the sequence "TAGAAT", which is one letter away from a TATA-box, at the -80 position.

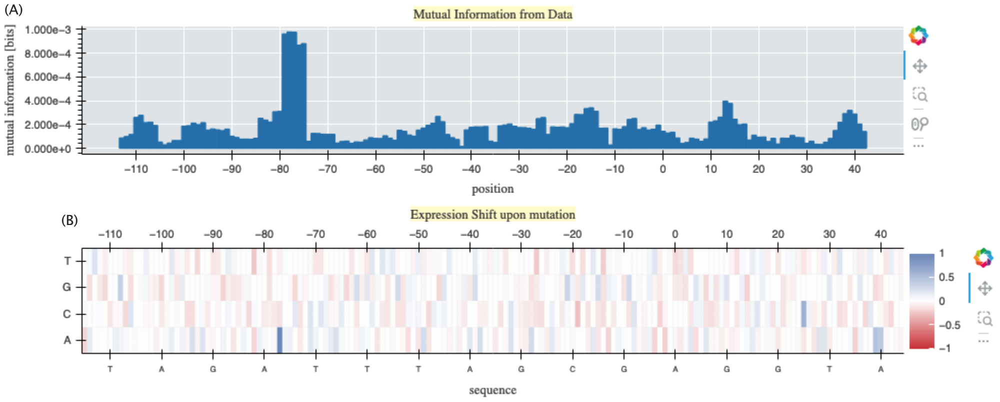

Figure R19: **Reg-Seq output for the araB promoter produced from growth experiments where the media contains glucose and no arabinose.** (A) Information footprint of the araB promoter. The mutual information has been smoothed with a window size of 2. (B) Expression shift matrix of the araB promoter. At position -77, a mutation into A creates a TATA-box, leading to a large positive change in expression levels. Both plots are obtained from our Reg-Seq website that showcases the experimental data produced in our lab.

8. Section 3.3 shows that overlapping sites are difficult to interpret. But aren't overlapping sites highly common in *E. coli* promoters? (especially if weaker sites are included). This warrants some discussion.

> **Author's response:** The reviewer is exactly right. The whole goal of the allied experimental project is to definitively determine for every promoter in *E. coli*, what is the underlying regulatory architecture. The already known regulatory architectures already tell us overlapping binding sites are a common feature, and the point of Section 3.3 is to show the difficulties associated with analyzing MPRA datasets when there are overlapping binding events. As is

the goal of the rest of the paper, this is part of our theory of the experiment. Here, we are providing a benchmark for what the footprints may look like when there are overlapping binding events. Our hope is that this can help us and others better anticipate the challenges that may arise when one is trying to figure out the positions of the transcription factor binding sites from MPRA summary statistics such as information footprints and expression shift matrices.

In our paper, we specifically address the overlapping binding events between the RNAP and a repressor. Given that simple repression is a very common architecture and that repressors often sterically block RNAP from accessing the promoter by binding to the same binding site as the RNAP, we deem this to be an important scenario to consider. In our revised paper, we offer a more precise discussion on the limits of MPRAs in the presence of overlapping binding events. Instead of choosing an arbitrary position within the RNAP binding site where the repressor may bind, we now slide the repressor along the RNAP binding site to see how much overlap can be tolerated before the two binding sites are no longer distinguishable from each other.

To do this, we design promoter sequences that maximize binding strengths based on the energy matrices of the RNAP and the repressor. At positions along the promoter that are only bound by RNAP, we choose the base that minimizes the binding energy of the RNAP. The same applies for positions that are only bound by the repressor. On the other hand, at overlapping binding sites, base identities that minimize the total binding energy by the RNAP and the repressor are chosen. The base identities of the rest of the promoter sequence are chosen at random. In Fig R20, we plot the information footprints and the expression shift matrices for promoters where the RNAP and repressor binding sites overlap to different degrees. In the information footprints, the signals from the two binding events are still clearly segmented and distinguishable from each other unless the vast majority of the binding sites are overlapping. In the expression shift matrices, even when the repressor binds directly on top of the -10 RNAP binding site, some signals from RNAP binding is still preserved.

It is true that other types of overlapping binding events may also occur. However, as mentioned in our response to an earlier comment, we suspect that the many of the "overlapping" binding sites displayed on RegulonDB actually reflect transcription factor binding under different growth conditions. That is to say, even though the *positions* of the binding sites may overlap, these transcription factors do not actually bind at the same time under the same condition, and therefore they cannot be qualified as overlapping binding events. Unfortunately, information on which binding site associates with which growth condition is lacking on RegulonDB, and therefore we are unable to provide an exact value on the frequency of overlapping binding sites. However, we again invite the reviewer to examine Fig R7, which shows the fraction of promoters that fall under different regulatory architectures. Over half of the architectures are regulated by only one transcription factor (either a repressor or an activator). This would imply that overlapping transcription factor binding events would not take place in the majority of the cases. Nevertheless, with our algorithm, it is possible to simulate the MPRA readout of any manner of binding events. For example, the case where activator and repressor binding sites overlap would be very similar to the case where RNAP and repressor binding sites overlap.

We are not trying to be encyclopedic about all possible scenarios in this paper, nor are we claiming that we have solved all problems for all circumstances. As seen in the Appendix R1, it would be surprising if we could successfully infer the regulatory architectures for all 3000 of our information footprints. Nevertheless, this exercise is meant to illustrate what we would expect for those cases in which there are overlapping binding sites. We would be delighted for other suggestions on how to handle tricky situations.

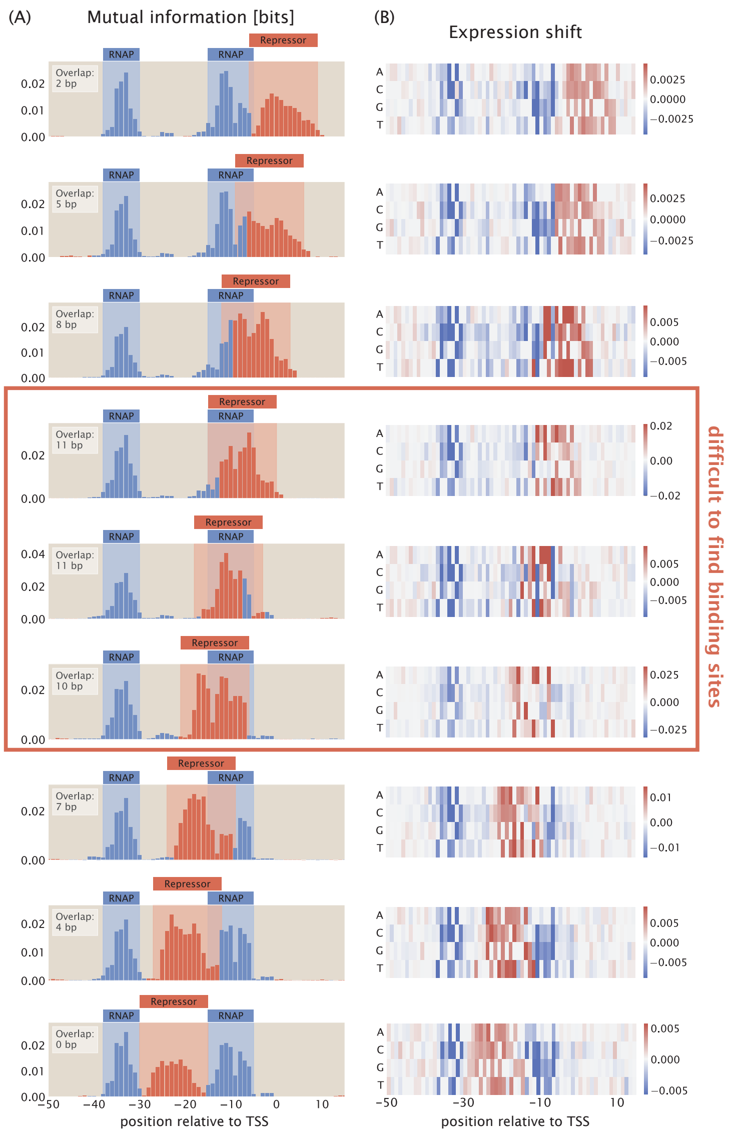

Figure R20: **Changing the degree of overlap between the RNAP and repressor binding sites.** (A - B) Information footprints and expression shift matrices of a simple repression promoter with overlapping binding sites. The promoters are designed to maximize binding strength given the known energy matrices of the RNAP [7] and the LacI repressor [6]. The degree of overlap in the information footprints and expression shift matrices in each row is noted at the upper left hand corner of the footprints.

Finally, there were a number of places where I noted small errors/typos:

1. In Figure 7(B) the panel titles should be swapped – the upper one depicts the case where $\Delta\varepsilon_{rd} = -10\,k_BT$, while in the bottom one $\Delta\varepsilon_{rd} = -20\,k_BT$.
2. In the denominator of equation (39), $\frac{R_I}{N_{NS}}e^{-\beta\Delta\varepsilon^I_{rd}}$ should be replaced by $\frac{R_A}{N_{NS}}e^{-\beta\Delta\varepsilon^I_{rd}}$.
3. In Figure 8(C) 5), $R_1 = 5$ instead of $R_1 = 0$.
4. Equation (42) seems incorrect. The numerator should not be 1 but the same as the numerator of equation (41).
5. Line 424 – concentration of the inducer goes up to $10K_A$instead of K_A
6. In line 305, 'strong binding' should be replaced by 'weak binding'.
7. Line 322 – it should state when $R_1 < R_2$ instead of when $R_1 > R_2$.

Author's response: We thank the reviewers for pointing out these errors. They have been corrected in the main text.

Regarding point 5, we know that in the plot we explore a range of inducer concentrations up to 10 K_A. However, here we were specifically referring to the part of the plot where inducer concentration increases from 10^{-2} K_A to 1 K_A, where average mutual information decreases. Notice that average mutual information stabilizes at around 0 bits when inducer concentration is increased beyond 1 K_A.

References

1. Kinney JB, Murugan A, Callan Jr CG, and Cox EC. Using deep sequencing to characterize the biophysical mechanism of a transcriptional regulatory sequence. Proc. Natl. Acad. Sci. U. S. A. 2010 May; 107:9158–63
2. Urtecho G, Tripp AD, Insigne KD, Kim H, and Kosuri S. Systematic Dissection of Sequence Elements Controlling σ70 Promoters Using a Genomically Encoded Multiplexed Reporter Assay in Escherichia coli. Biochemistry 2019 Mar; 58:1539–51
3. Lagator M, Sarikas S, Steinrueck M, Toledo-Aparicio D, Bollback JP, Guet CC, and Tkačik G. Predicting bacterial promoter function and evolution from random sequences. Elife 2022 Jan; 11
4. Lagator M, Sarikas S, Steinrück M, Toledo-Aparicio D, Bollback JP, Tkacik G, and Guet CC. Structure and Evolution of Constitutive Bacterial Promoters. bioRxiv 2020 May :2020.05.19.104232
5. Zhao Y, Granas D, and Stormo GD. Inferring binding energies from selected binding sites. PLoS Comput. Biol. 2009 Dec; 5:e1000590
6. Barnes SL, Belliveau NM, Ireland WT, Kinney JB, and Phillips R. Mapping DNA sequence to transcription factor binding energy in vivo. PLoS Comput. Biol. 2019 Feb; 15:e1006226
7. Brewster RC, Jones DL, and Phillips R. Tuning promoter strength through RNA polymerase binding site design in *Escherichia coli*. PLoS Comput. Biol. 2012 Dec; 8:e1002811
8. Buchler NE, Gerland U, and Hwa T. On schemes of combinatorial transcription logic. Proc. Natl. Acad. Sci. U. S. A. 2003 Apr; 100:5136–41
9. Ronde W de, Rein ten Wolde P, and Mugler A. Protein logic: a statistical mechanical study of signal integration at the single-molecule level. Biophys. J. 2012 Sep; 103:1097–107
10. Lindsley JE and Rutter J. Whence cometh the allosterome? Proc. Natl. Acad. Sci. U. S. A. 2006 Jul; 103:10533–5

11. Raser JM and O'Shea EK. Noise in gene expression: origins, consequences, and control. Science 2005 Sep; 309:2010–3

12. Fu AQ and Pachter L. Estimating intrinsic and extrinsic noise from single-cell gene expression measurements. Stat. Appl. Genet. Mol. Biol. 2016 Dec; 15:447–71

13. Elowitz MB, Levine AJ, Siggia ED, and Swain PS. Stochastic gene expression in a single cell. Science 2002 Aug; 297:1183–6

14. Cherry JL and Adler FR. How to make a biological switch. J. Theor. Biol. 2000 Mar; 203:117–33

15. Elowitz MB and Leibler S. A synthetic oscillatory network of transcriptional regulators. Nature 2000 Jan; 403:335–8

16. Kuchina A, Espinar L, Çağatay T, Balbin AO, Zhang F, Alvarado A, Garcia-Ojalvo J, and Süel GM. Temporal competition between differentiation programs determines cell fate choice. Mol. Syst. Biol. 2011 Dec; 7:557

17. Eldar A, Chary VK, Xenopoulos P, Fontes ME, Losón OC, Dworkin J, Piggot PJ, and Elowitz MB. Partial penetrance facilitates developmental evolution in bacteria. Nature 2009 Jul; 460:510–4

18. Huang S, Guo YP, May G, and Enver T. Bifurcation dynamics in lineage-commitment in bipotent progenitor cells. Dev. Biol. 2007 May; 305:695–713

19. Alon U. An Introduction to Systems Biology: Design Principles of Biological Circuits. CRC Press, 2006

20. Covert M. Computational Systems Biology. Academic Press, 2005

21. Garcia HG, Sanchez A, Boedicker JQ, Osborne M, Gelles J, Kondev J, and Phillips R. Operator sequence alters gene expression independently of transcription factor occupancy in bacteria. Cell Rep. 2012 Jul; 2:150–61

22. Hammar P, Walldén M, Fange D, Persson F, Baltekin O, Ullman G, Leroy P, and Elf J. Direct measurement of transcription factor dissociation excludes a simple operator occupancy model for gene regulation. Nat. Genet. 2014 Apr; 46:405–8

23. Kuhlman T, Zhang Z, Saier Jr MH, and Hwa T. Combinatorial transcriptional control of the lactose operon of *Escherichia coli.* Proc. Natl. Acad. Sci. U. S. A. 2007 Apr; 104:6043–8

24. Garcia HG and Phillips R. Quantitative dissection of the simple repression input-output function. Proc. Natl. Acad. Sci. U. S. A. 2011 Jul; 108:12173–8

25. Razo-Mejia M, Barnes SL, Belliveau NM, Chure G, Einav T, Lewis M, and Phillips R. Tuning Transcriptional Regulation through Signaling: A Predictive Theory of Allosteric Induction. Cell Syst 2018 Apr; 6:456–469.e10

26. Brewster RC, Weinert FM, Garcia HG, Song D, Rydenfelt M, and Phillips R. The transcription factor titration effect dictates level of gene expression. Cell 2014 Mar; 156:1312–23

27. Hill A and Paganini-Hill A. The possible effects of the aggregation of the molecules of haemoglobin on its dissociation curves. J. Physiol. 1910

28. Hill AV. The Combinations of Haemoglobin with Oxygen and with Carbon Monoxide. I. Biochem. J 1913 Oct; 7:471–80

29. McGhee JD and Hippel PH von. Theoretical aspects of DNA-protein interactions: co-operative and non-co-operative binding of large ligands to a one-dimensional homogeneous lattice. J. Mol. Biol. 1974 Jun; 86:469–89

30. Ackers GK, Johnson AD, and Shea MA. Quantitative model for gene regulation by lambda phage repressor. Proc. Natl. Acad. Sci. U. S. A. 1982 Feb; 79:1129–33

31. Shea MA and Ackers GK. The OR control system of bacteriophage lambda. A physical-chemical model for gene regulation. J. Mol. Biol. 1985 Jan; 181:211–30

32. Gardner TS, Cantor CR, and Collins JJ. Construction of a genetic toggle switch in *Escherichia coli.* Nature 2000 Jan; 403:339–42

33. Goldbeter A. A model for circadian oscillations in the Drosophila period protein (PER). Proceedings of the Royal Society of London. Series B: Biological Sciences 1997 Jan; 261:319–24

34. Süel GM, Garcia-Ojalvo J, Liberman LM, and Elowitz MB. An excitable gene regulatory circuit induces transient cellular differentiation. Nature 2006 Mar; 440:545–50

35. Walczak AM, Tkacik G, and Bialek W. Optimizing information flow in small genetic networks. II. Feed-forward interactions. Phys. Rev. E Stat. Nonlin. Soft Matter Phys. 2010 Apr; 81:041905

36. Sivak DA and Thomson M. Environmental statistics and optimal regulation. PLoS Comput. Biol. 2014 Sep; 10:e1003826

37. Keymer JE, Endres RG, Skoge M, Meir Y, and Wingreen NS. Chemosensing in *Escherichia coli*: two regimes of two-state receptors. Proc. Natl. Acad. Sci. U. S. A. 2006 Feb; 103:1786–91

38. Swem LR, Swem DL, Wingreen NS, and Bassler BL. Deducing receptor signaling parameters from in vivo analysis: LuxN/AI-1 quorum sensing in Vibrio harveyi. Cell 2008 Aug; 134:461–73

39. Mello BA and Tu Y. Quantitative modeling of sensitivity in bacterial chemotaxis: the role of coupling among different chemoreceptor species. Proc. Natl. Acad. Sci. U. S. A. 2003 Jul; 100:8223–8

40. Tu Y. The nonequilibrium mechanism for ultrasensitivity in a biological switch: sensing by Maxwell's demons. Proc. Natl. Acad. Sci. U. S. A. 2008 Aug; 105:11737–41

41. Martin RG, Bartlett ES, Rosner JL, and Wall ME. Activation of the *Escherichia coli* marA/soxS/rob regulon in response to transcriptional activator concentration. J. Mol. Biol. 2008 Jul; 380:278–84

42. Wall ME, Markowitz DA, Rosner JL, and Martin RG. Model of transcriptional activation by MarA in *Escherichia coli*. PLoS Comput. Biol. 2009 Dec; 5:e1000614

43. Doughty BR, Hinks MM, Schaepe JM, Marinov GK, Thurm AR, Rios-Martinez C, Parks BE, Tan Y, Marklund E, Dubocanin D, Bintu L, and Greenleaf WJ. Single-molecule chromatin configurations link transcription factor binding to expression in human cells. 2024 Feb

44. Hyman AA, Weber CA, and Jülicher F. Liquid-liquid phase separation in biology. Annu. Rev. Cell Dev. Biol. 2014; 30:39–58

45. Mirny LA. Nucleosome-mediated cooperativity between transcription factors. Proc. Natl. Acad. Sci. U. S. A. 2010 Dec; 107:22534–9

46. Grigorova IL, Phleger NJ, Mutalik VK, and Gross CA. Insights into transcriptional regulation and sigma competition from an equilibrium model of RNA polymerase binding to DNA. Proc. Natl. Acad. Sci. U. S. A. 2006 Apr; 103:5332–7

47. Zuo Z and Stormo GD. High-resolution specificity from DNA sequencing highlights alternative modes of Lac repressor binding. Genetics 2014 Nov; 198:1329–43

48. Weinert FM, Brewster RC, Rydenfelt M, Phillips R, and Kegel WK. Scaling of gene expression with transcription-factor fugacity. Phys. Rev. Lett. 2014 Dec; 113:258101

49. Kreamer NN, Phillips R, Newman DK, and Boedicker JQ. Predicting the impact of promoter variability on regulatory outputs. Sci. Rep. 2015 Dec; 5:18238

50. Eck E, Liu J, Kazemzadeh-Atoufi M, Ghoreishi S, Blythe SA, and Garcia HG. Quantitative dissection of transcription in development yields evidence for transcription-factor-driven chromatin accessibility. Elife 2020 Oct; 9

51. Meijsing SH, Pufall MA, So AY, Bates DL, Chen L, and Yamamoto KR. DNA binding site sequence directs glucocorticoid receptor structure and activity. Science 2009 Apr; 324:407–10

52. Gunawardena J. A linear framework for time-scale separation in nonlinear biochemical systems. PLoS One 2012 May; 7:e36321

53. Mahdavi S, Salmon GL, Daghlian P, Garcia HG, and Phillips R. Flexibility and sensitivity in gene regulation out of equilibrium. bioRxiv 2023 Apr :2023.04.11.536490

54. Ireland WT, Beeler SM, Flores-Bautista E, McCarty NS, Röschinger T, Belliveau NM, Sweredoski MJ, Moradian A, Kinney JB, and Phillips R. Deciphering the regulatory genome of *Escherichia coli*, one hundred promoters at a time. Elife 2020 Sep; 9

55. Gerland U and Hwa T. On the selection and evolution of regulatory DNA motifs. J. Mol. Evol. 2002 Oct; 55:386–400

56. Lynch M and Hagner K. Evolutionary meandering of intermolecular interactions along the drift barrier. Proc. Natl. Acad. Sci. U. S. A. 2015 Jan; 112:E30–8

57. Berg J, Willmann S, and Lässig M. Adaptive evolution of transcription factor binding sites. BMC Evol. Biol. 2004 Oct; 4:42

58. Hippel PHV, Revzin A, Gross CA, and Wang AC. Non-specific DNA Binding of Genome Regulating Proteins as a Biological Control Mechanism: 1. The *lac* Operon: Equilibrium Aspects. Proceedings of the National Academy of Sciences 1974; 71:4808–12

59. Ptashne M. A Genetic Switch: Phage Lambda Revisited. New York: Cold Spring Harbor Laboratory Press, 2004

60. Dodd IB, Shearwin KE, Perkins AJ, Burr T, Hochschild A, and Egan JB. Cooperativity in long-range gene regulation by the lambda CI repressor. Genes Dev. 2004 Feb; 18:344–54

61. Swint-Kruse L and Matthews KS. Allostery in the LacI/GalR family: variations on a theme. Curr. Opin. Microbiol. 2009 Apr; 12:129–37

62. Dunn TM, Hahn S, Ogden S, and Schleif RF. An operator at -280 base pairs that is required for repression of araBAD operon promoter: addition of DNA helical turns between the operator and promoter cyclically hinders repression. Proc. Natl. Acad. Sci. U. S. A. 1984 Aug; 81:5017–20

63. Ogden S, Haggerty D, Stoner CM, Kolodrubetz D, and Schleif R. The *Escherichia coli* L-arabinose operon: binding sites of the regulatory proteins and a mechanism of positive and negative regulation. Proc. Natl. Acad. Sci. U. S. A. 1980 Jun; 77:3346–50

64. Schleif R and Lis JT. The regulatory region of the L-arabinose operon: a physical, genetic and physiological study. J. Mol. Biol. 1975 Jul; 95:417–31

65. Müller J, Oehler S, and Müller-Hill B. Repression of lac promoter as a function of distance, phase and quality of an auxiliary lac operator. J. Mol. Biol. 1996 Mar; 257:21–9

66. Oehler S, Alberti S, and Müller-Hill B. Induction of the lac promoter in the absence of DNA loops and the stoichiometry of induction. Nucleic Acids Res. 2006 Jan; 34:606–12

67. Oehler S, Amouyal M, Kolkhof P, Wilcken-Bergmann B von, and Müller-Hill B. Quality and position of the three lac operators of *E. coli* define efficiency of repression. EMBO J. 1994 Jul; 13:3348–55

68. Oehler S, Eismann ER, Krämer H, and Müller-Hill B. The three operators of the lac operon cooperate in repression. EMBO J. 1990 Apr; 9:973–9

69. Vilar JMG and Leibler S. DNA looping and physical constraints on transcription regulation. J. Mol. Biol. 2003 Aug; 331:981–9

70. Vilar JMG and Saiz L. The unreasonable effectiveness of equilibrium gene regulation through the cell cycle. bioRxiv 2023 Apr :2023.03.31.535089

71. Vilar JMG and Saiz L. Systems biophysics of gene expression. Biophys. J. 2013 Jun; 104:2574–85

72. Bintu L, Buchler NE, Garcia HG, Gerland U, Hwa T, Kondev J, and Phillips R. Transcriptional regulation by the numbers: models. Curr. Opin. Genet. Dev. 2005 Apr; 15:116–24

73. Bintu L, Buchler NE, Garcia HG, Gerland U, Hwa T, Kondev J, Kuhlman T, and Phillips R. Transcriptional regulation by the numbers: applications. Curr. Opin. Genet. Dev. 2005 Apr; 15:125–35

74. Tu Y. Quantitative modeling of bacterial chemotaxis: signal amplification and accurate adaptation. Annu. Rev. Biophys. 2013 Feb; 42:337–59

75. Hathcock D, Yu Q, Mello BA, Amin DN, Hazelbauer GL, and Tu Y. A nonequilibrium allosteric model for receptor-kinase complexes: The role of energy dissipation in chemotaxis signaling. Proc. Natl. Acad. Sci. U. S. A. 2023 Oct; 120:e2303115120

76. Li GW, Burkhardt D, Gross C, and Weissman JS. Quantifying absolute protein synthesis rates reveals principles underlying allocation of cellular resources. Cell 2014 Apr; 157:624–35

77. Schmidt A, Kochanowski K, Vedelaar S, Ahrné E, Volkmer B, Callipo L, Knoops K, Bauer M, Aebersold R, and Heinemann M. The quantitative and condition-dependent *Escherichia coli* proteome. Nat. Biotechnol. 2016 Jan; 34:104–10

78. Rosenfeld N, Young JW, Alon U, Swain PS, and Elowitz MB. Accurate prediction of gene feedback circuit behavior from component properties. Mol. Syst. Biol. 2007 Nov; 3:143

79. Balakrishnan R, Mori M, Segota I, Zhang Z, Aebersold R, Ludwig C, and Hwa T. Principles of gene regulation quantitatively connect DNA to RNA and proteins in bacteria. Science 2022 Dec; 378:eabk2066

80. Yona AH, Alm EJ, and Gore J. Random sequences rapidly evolve into de novo promoters. Nat. Commun. 2018 Apr; 9:1530

81. Vvedenskaya IO, Goldman SR, and Nickels BE. Analysis of Bacterial Transcription by "Massively Systematic Transcript End Readout," MASTER. Methods Enzymol. 2018 Oct; 612:269–302
