# Supplementary material for: Deciphering regulatory architectures of bacterial promoters from synthetic expression patterns
Source: PLoS Comput Biol. 2024 Dec 26;20(12):e1012697. doi: 10.1371/journal.pcbi.1012697 (PMC11709304; doi:10.1371/journal.pcbi.1012697)
Supplement: Attachment — Submitted filename: response_to_reviewers.pdf [file pcbi.1012697.s014.pdf]

Thank you very much for submitting your manuscript "Deciphering regulatory architectures from synthetic single-cell expression patterns" for consideration at PLOS Computational Biology.

As with all papers reviewed by the journal, your manuscript was reviewed by members of the editorial board and by several independent reviewers. In light of the reviews (below this email), we would like to invite the resubmission of a significantly-revised version that takes into account the reviewers' comments.

Please address all the reviewer comments carefully, including their suggestions on improving the paper's flow and presentation and on making the computational pipeline more accessible. This will be the last round of major revisions for this manuscript.

Author's response: We thank the editor and the three reviewers for their useful and detailed comments on our work. We have included below a list of point-by-point responses to the new reviewer's comments. In particular, based on the reviewer's suggestions, we have made the following major edits to improve the paper's flow and presentation:

- We have changed our title to "Deciphering regulatory architectures of bacterial promoters from synthetic expression patterns" in order to clarify that our paper primarily focuses on microbial systems and does not involve single-cell methods.
- We have added to our results section more detailed explanations of how our results translate to actionable strategies for MPRA design and interpretation.
- We have moved a number of derivations from the main text to the Supplemental Information, including the analytical results on hitch-hiking effects, the states-and-weights diagram for a double repression promoter, the method of chemical potential, the derivation of the thermodynamic model for an inducible repressor, and the derivation of RNAP being bound under non-equilibrium. We hope this helps to address the reviewer's concerns that our results are buried in the derivations.
- We have reorganized our Supplemental Information for better clarity and we added more explicit call outs to each of the Supplemental Appendices within the main paper itself.

In addition, in order to make the computational pipeline more accessible, we have made the following changes to our Github repository:

- We have organized our code into a directory that contains all the jupyter notebooks for producing the main text plots and a directory that contains all the notebooks for producing the supplementary plots.
- We have added more detailed instructions for accessing and running our code in the README.md files.
- We have added markdown explanations in our notebooks to help users understand and be able to run our code.
- We have added docstrings, which include descriptions of input and output, to all of the functions in the custom 'tregs' python package.

It has been greatly helpful to engage with the reviewer's comments. We believed our paper is substantially improved and we hope that it is now ready for publication in PLOS Computational Biology.

Reviewer 1 comments

The authors have adequately addressed all the comments.

We thank the reviewer for their very helpful comments in the previous round of revision and for this positive feedback.

Reviewer 3 comments

In this paper the authors provide thermodynamic models for 6 simple promoter architectures that are commonly found in *E. coli*: one repressor, one activator, two repressors act together, two repressors that have an xor relationship and an activator, a repressor and double activation. The binding energy is derived from the sequence motif and it is assumed that only binding of the TF at the original site can induce or repress transcription. Binding sites are assumed not to overlap, but new binding sites may appear in the genome and thus compete with the original site for limited TF-proteins. The model appears to require a number of detailed input parameters such as the number of RNA-polymerase and transcription factor molecules in a cell (maybe a table with required input parameters would be nice to have).

Author's response: We appreciate the reviewer's summary of our work. That said, there appear to be some points of confusion that we would like to clear up. The reviewer is right to state that we are examining the six most common regulatory architectures. As shown in Fig R1, which is adapted from Mahdavi, Salmon et al. [1], the six common regulatory architectures that we listed in Fig 2 of our main text cover over 80% of the promoters in *E. coli*. Hence, although the reviewer's words make it sound as though we chose the regulatory architectures to examine based on simplicity, we chose them based on the regulatory reality that is relevant to experimental interpretation.

In addition, in our simulations, we are not "assuming" that transcription factors can only regulate expression when they are bound at the original binding site or that binding sites do not overlap (please see Figure 14 of our paper in direct contradiction with this remark). Instead, we are *prescribing* the conditions under which the transcription factor binds to the original unmutated binding site and when the binding sites are non-overlapping in our simulations it is because we are mimicking known regulatory circumstances and we are interested in seeing what the summary statistics would look like in these circumstances. It is just as easy for us to prescribe that the transcription factors can regulate expression when they are bound away from the original binding site or when there are overlapping binding sites, which are in fact also common. For example, in Fig 13 of our paper, we slide RNAP across the entire promoter by calculating the binding energies based on the energy matrix at all possible positions and we can just as easily slide transcription factors across the promoter using the same procedure. In Fig 14, we have engineered binding sites with various degrees of overlap and plot the summary statistics. So to reiterate, although the reviewer seems to imply that there is some restriction on the class of architectures or assumptions, there are not. The comment that we are considering binding sites that don't overlap is strange given that this comment is in direct contradiction with actual evidence. Our Figure 14 is precisely about overlapping binding sites which we consider one of the most interesting and useful plots in the paper.

Secondly, we thank the reviewer for the suggestion of summarizing the required input parameters for our model. We have created a figure that highlights the input parameters, shown here as Fig R2 and included as Fig 2 in the main text. We hope the figure serve the same function as the table that the reviewer had in mind.

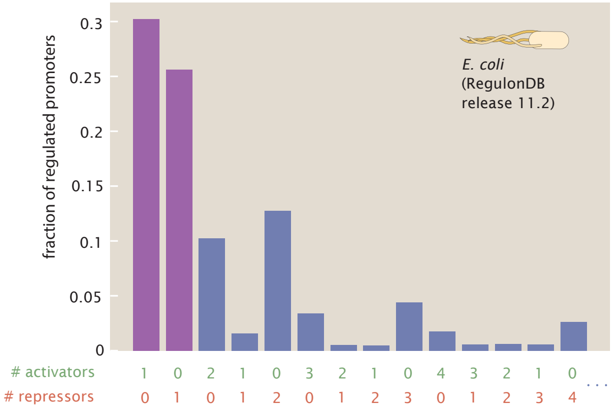

Figure R1: **Distribution of regulatory architectures in *E. coli* based on a census from RegulonDB.** The figure is adapted from Mahdavi, Salmon et al [1]. The histogram summarizes the frequency of different regulatory architectures starting with simple activation and simple repression highlighted in purple. Note that we worry that databases such as RegulonDB are incomplete since they do not say how growth conditions alter which transcription factors are bound. Our experimental and theoretical work aims to clarify this.

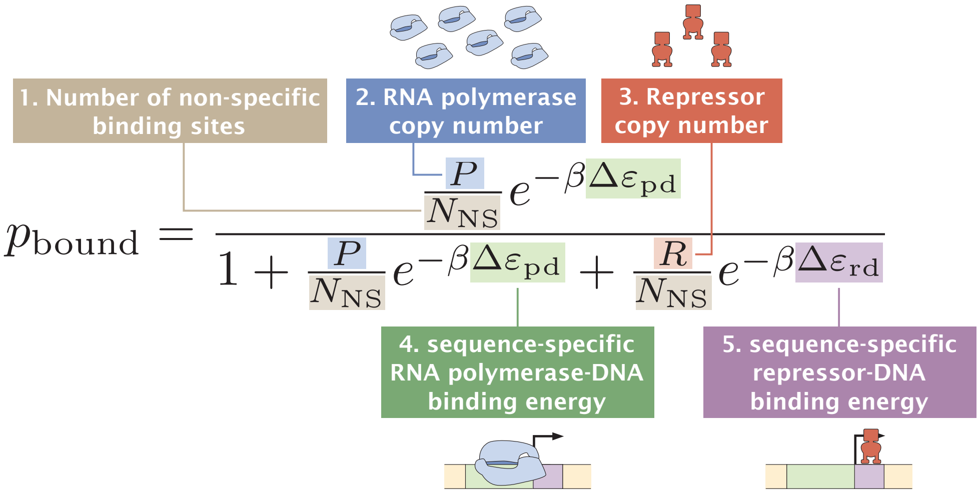

Figure R2: **Parameters required for the thermodynamic model of a promoter with the simple repression regulatory architecture.** For a promoter with the simple repression regulatory architecture, five parameters are required. These parameters are (1) the number of non-specific binding sites, N_{NS}, (2) the copy number of the RNA polymerase, P, (3) the copy number of the repressor, R, (4) the sequence-specific binding energy of the RNA polymerase at its binding site, $\Delta\varepsilon_{\text{pd}}$, and (5) the sequence-specific binding energy of the repressor at its binding site, $\Delta\varepsilon_{\text{rd}}$. All of these parameters will affect the output of an MPRA assay.

Based on this the authors can then construct a synthetic RNA-seq data-set that predicts expression for all variants of the simulated promoter. This data is then used to calculate the mutual information between the sequences and the expression level, which can in a real life MPRA be used to predict the binding sites. The authors exemplify the LacZYA promoter. Here it would be nice to compare the simulated data to what the real data from their earlier papers show.

Author's response: We appreciate the reviewer's suggestion and have made a figure with side-by-side comparison of the simulated information footprints and footprints built from experimental datasets obtain by Ireland et al. [2] The figure is shown below in Fig R3 and included in Appendix S3.3 of the Supplemental Information.

It is crucial to note that direct comparison between these simulated footprints and experimental data cannot be done rigorously without precisely knowing the transcription factor copy numbers in the experimental situations as well as the energy matrix for the particular transcription factor(s) that control the gene of interest. This is not our objective since the whole point of the Reg-Seq and other MPRAs is to discover the architectures in cases where we don't even know which transcription factors are in play, let alone their copy numbers. Hence, although we agree with the reviewer that it is interesting to see these examples side by side, it can also be deceiving. This is not a strict comparison because for such a comparison to be made we would need to make sure to use precisely the correct energy matrix for each and every transcription factor in question as well as the precise transcription factor copy numbers. Again, the whole point of the MPRAs we and others are doing is to discover the regulatory architectures. Hence, going in, we know neither the energy matrices nor the transcription factor identities nor their copy numbers.

Of course, for gold standard genes where we already know these parameters, we and others have worked diligently to explore the limits of validity of such models. In Fig R9 included below, we provide one example to help the reviewer see the standards we bring to our work on regulatory dissections. Those two graphs show parameter-free theoretical predictions that were done before the corresponding experiments. Both of these figures make it crystal clear that the level of expression depends upon transcription factor copy number and DNA binding strength. The results in Figure R9(b) show precisely the importance of the titration effect that the reviewer later in their review refers to as a "thought experiment" which it most decidedly is not. These experiments demonstrate the kinds of effects that are present in MPRA studies when using plasmids rather than genome integration. All told, the present work aims to explore how MPRAs are governed by these unknown parameters so that when dealing with experimental data, we and others are better able to anticipate and understand the implications of both experimental and biological parameters.

Until here this sounds like a general enough approach that might also work in eukaryotes, even though some of the assumptions, especially the lack of epistatic interactions and the strong position dependence, are unlikely to be correct.

Author's response: We are concerned that the reviewer is perhaps not fully aware of the long history of the use of thermodynamic models, not just in prokaryotes, but in eukaryotes as well. As we have emphasized already in the paper and in both this and the previous round of responses to reviewer's comments, we are not patriots of thermodynamic models. We love chemical master equations, we love graph theory and broken detailed balance. And, we know how to use these approaches and do use them [1, 3]. We wrote a whole long section in the SI that addresses precisely the point of both the successes and shortcomings of this class of models. We are concerned that the reviewer did not look at this appendix. The point of using thermodynamic models here is not to establish the definitive, end-all set of descriptions of transcription. The point is to examine exactly how the summary statistics used in MPRAs depend both upon biological and experimental parameters. These are questions that are critical to our bigger goal of the high-throughput dissection of the regulatory architectures in *E. coli* and *Drosophila*. As we have demonstrated in our response to the previous round of reviews and in the paper itself, we can make (and have made) these same calculations using other kinds of models, but the main point of the exercise is to learn something about how mutating the promoter region alters the level of expression.

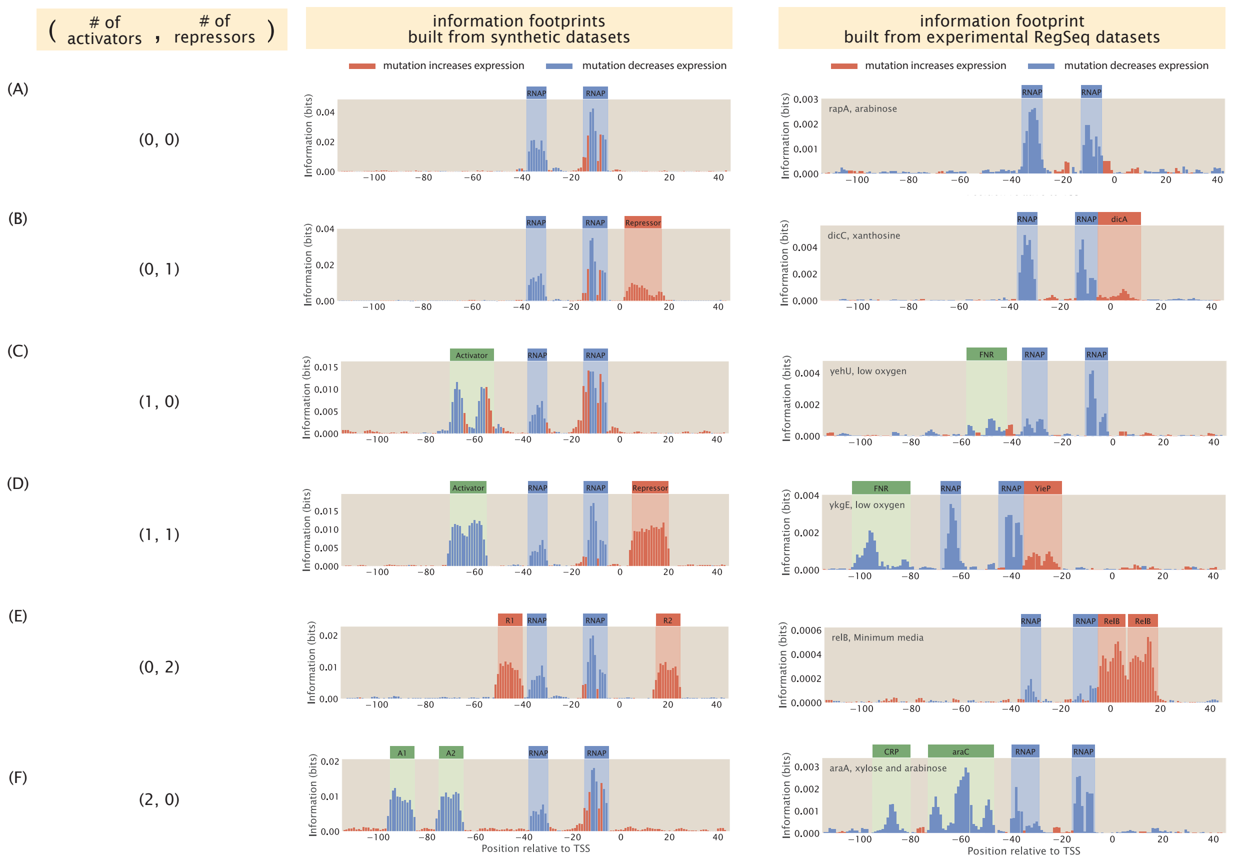

Figure R3: **Comparison of information footprints built from synthetic datasets and information footprints built from experimental datasets by Ireland et al.** Comparison of synthetic footprints and experimental footprints for the six most common regulatory architectures. The experimental footprints are built from datasets obtained through Reg-Seq, an MPRA developed by Ireland et al. [2] from our group. Note that to directly compare the theoretical and experimental profiles would require us to know the copy numbers of activators and repressors which we do not know when doing an MPRA of previously unannotated architectures and hence the direct quantitative comparison is not the objective of this work, nor is it even possible. Making head-to-head statistical comparisons of the simulated data and the experimental data is not useful.

As a brief survey, here we point to a number of works using these models in eukaryotes including Segal et al. [4], Eck et al. [5], White et al. [6], Park et al. [7], Kim et al. [8], Kuang et al. [9], He et al. [10], Fakhouri et al. [11], Doughty et al. [12], Gertz et al. [13], Raveh-Sadka et al. [14], Chickarmane et al. [15]. The list is much longer. That said, the thermodynamic framework remains to this day, whether it is said so explicitly or not, the fundamental null hypothesis in organisms of all types as illustrated by this short list of examples. Not only are these models used explicitly, but the interpretation of ChIP-Seq data in terms of occupancy of proteins on DNA, the construction of dynamical models using differential equations all appeal to what we might call the occupancy hypothesis where the occupancy is characterized by a rational function of the form $F(x)/G(x)$, where x is the concentration of some transcription factor and almost always there is some equilibrium binding function hiding in these assumptions. It is simply not a true statement at all that these kinds of models are limited to prokaryotes. This kind of dismissive remark should be based on literature citations and scholarship. Second, the whole point of the kind of exercise we are engaged in is to remove this discussion from

philosophical discussions based on people's personal beliefs about the validity or the lack thereof of such models and to do the hard work of constructing a theory-experiment dialogue to map out the space of failures of such models. To do so, we need a "theory of the experiment." This reviewer has raised many interesting points that we agree with and the whole point of our work is to explore them. For example, transcription factor copy number is something we and everyone else do not know when doing MPRAs. We need to figure out how varying the copy number alters the MPRA signal. Similarly, a priori when doing an MPRA, we (and everyone else) do not know the binding site strength (or K_d) of TFs and the basal transcription apparatus for their binding sites. Again, the point of our analysis is to provide a theoretical reckoning of the strength of these effects so that we and others know what to expect in these financially and intellectually costly experiments.

Secondly, we agree with the reviewer that possible epistatic interactions between base positions are important to consider. However, existing literature such as Benos et al. [16], Takeda et al. [17] and von Hippel and Berg [18] have shown that a linear model is largely sufficient to describe protein-DNA interactions in many cases. As a result, we believe that the additive model is a useful baseline to describe sequence-energy mappings. Nevertheless, as we mentioned in our responses to the previous round of reviewer comments, we are not patriots of the kind of thermodynamic models that we are using. These models are useful, but we are fully aware of the literature that illustrates the limitations of the additive model, such as the work by Polly Fordyce [19] and Sebastian Maerkl [20]. We are very excited to explore the limitations of the additive assumption in our future work.

That said, the reviewer should be aware of the practical roadblocks to getting datasets that would allow this kind of models. One potential issue that we would like to clarify is that it would require a lot more data to build a more generalized thermodynamic model. We have already performed such inferences of generalized energy matrices and since inferring these energetic descriptions involves pairwise correlations, we need far more data. As shown in Fig R4, even without considering epistasis effects, our estimate for the cost of library synthesis for a genome-wide treatment of *E. coli* is in excess of 50 thousand dollars. To obtain sufficient data required for inferring non-additive models is currently cost-prohibitive. However, our *computational* framework described in this work would still hold if the additive energy models were to be substituted by non-additive models. The only difference is that the sequence-dependent energies used in the thermodynamic models are calculated differently with respect to the underlying sequences and the rest of the analysis would not be affected. One direction that we are excited to pursue in a future paper is to examine how the features of the synthetic datasets would differ when the additive models are replaced by the non-additive models. However, we believe this to be outside of the scope of the current paper and is for sure beyond the scope of current data.

Finally, we wholeheartedly agree with the reviewer that the question of binding site position is a fascinating and important one that we have considered both experimentally and theoretically. As demonstrated in Fig 13 of our main text, we can slide the energy matrix across the promoter and still observe signals at spurious binding sites that are away from the original binding positions. Though the determination of energy models with epistasis is an important next step requiring much more data than many MPRAs yield, the approach used here is the best current way we are aware of for exploring the challenging problem of binding site position dependence, a much trickier problem that is certainly not reserved for eukaryotes. Indeed, in our own earlier work we have worked diligently both theoretically and experimentally to rigorously characterize the role of such position dependence. For example, see Fig R5 from our earlier Cell Reports paper [21]. In multiple papers on DNA looping, we and others showed we could dissect the role of binding site position quantitatively [22]. When one has an energy matrix, epistasis or not, it provides a falsifiable model of binding for every position on a genome.

Next, the authors go through experimental design aspects that I feel are rather specific to microbial systems: specifically finding the optimal mutation rate to identify the TF footprints. Mutation rate and sequencing depth go hand in hand, and the authors also provide advice on that. However, what I am missing here is a

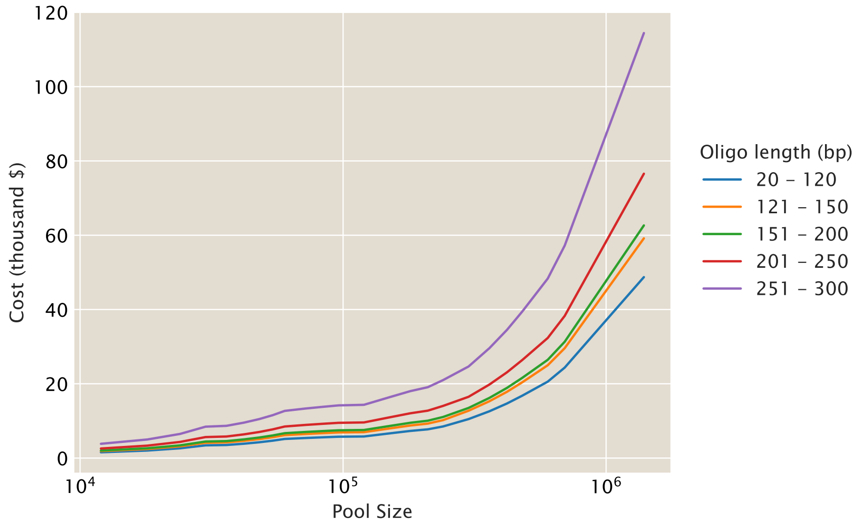

Figure R4: **Cost of synthesizing mutant library of different sizes.** The numbers are obtained from the Twist oligo pool cost sheet. Note that these costs take into account academic discounts that are offered for the synthesis of large pools.

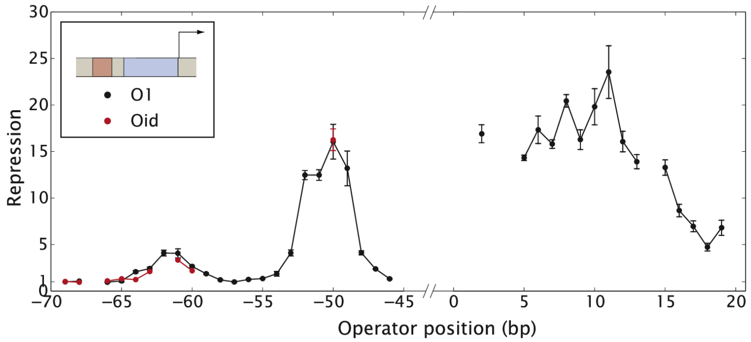

Figure R5: **Binding site position leads to different repression behavior along the promoter.** Level of repression at different binding site positions for an O1 Lac operator and an Oid Lac operator. The figure is adapted from Garcia et al. [21].

discussion about how much this depends on biological properties: i.e. the size and information content of the TF motif as well as the expression level of the associated TF. Only in a later chapter it is mentioned that those factors are a cause for differences in the detection power, biasing the detection towards highly expressed TFs with strong binding sites.

Author's response: The reviewer is right to point out that we use the experimental and biological parameters that are mostly relevant for microbial systems. Given that there is far more microbial biomass on our planet than animal biomass and the overwhelming importance of microbes to both health and the environment, it is hard to see why researchers should feel that working on microbes is somehow an intellectual backwater. Nevertheless, for the sake of clarity, we have acknowledged in our abstract and introduction that our work focuses on microbial systems. That said, as we will explain in detail below, one of the authors of this paper is fully engaged in the use of these methods in dissecting regulatory architectures and the whole reason we did several of the calculations in this paper (i.e. biased mutations) is because of the work in the eukaryotic case.

Furthermore, we are confident that the general approach proposed in this work can be adapted to study eukaryotic systems as well, especially since MPRAs and thermodynamic models have both been previously utilized to study eukaryotic transcription. In a highly-cited paper, Melnikov et al. [23] used MPRAs to dissect transcriptional regulatory elements in human cells. As noted above, the laboratory of Hernan Garcia, who is one of the authors on this paper, is actively engaged in developing MPRAs in *Drosophila*. For example, Falo-Sanjuan et al. [24], a recent paper from the Garcia lab, developed a method to introduce mutations at target sites within the genome of *Drosophila* whole animals and cell culture, which lays important experimental foundation to perform MPRAs in multicellular organisms. There are also an abundance of literature that tests the use of thermodynamic models in the study of transcriptional regulation in eukaryotic systems. As mentioned in our responses to the first round of reviewer comments, Doughty et al. [12] use thermodynamic models as a null model to describe the connection between the nucleosome and transcription factor occupancy and expression level in human cells. Furthermore, the Garcia lab has also been using, testing and challenging thermodynamic models in eukaryotic systems for many years [25].

We agree with the reviewer that the optimal mutation rate would depend on biological parameters such as the expression level of the transcription factor. We refer the reviewer to Appendix S4.2 of our Supplemental Information, where we have already discussed how the optimal mutation rate varies as a function of the parameters involved in our thermodynamic models, such as the binding energies and the copy numbers of the RNAP and the transcription factors. It is also really important to bear in mind the goal. We are trying to solve problems in which we, nor anyone else, know the architecture of a given promoter of interest. Similarly, we don't know the copy number of the TF that might regulate a gene of interest since the whole point is to discover them. That being said, from careful proteomic studies, as shown in Fig R6 (a figure also included in the previous round of revisions), we do have a reasonable idea of both the range of copy numbers and their associated K_d [26, 27]. As a result, when we search for "optimal" mutation rate, it really is quantitative guess work given all that we know and do not know. We remain somewhat confused by the reviewer's response. When anyone undertakes these kinds of experiments to make discoveries in cases where we truly know nothing going in, we are *all* flying blind. The goal of our calculations is to get a sense of how the experimental data we and others will be obtaining will depend upon these unknown biological parameters.

Finally, we appreciate the reviewer's suggestion that we take a closer look at how optimal mutation rate depends on the size and information content of the binding site. We have added another figure in our Supplemental Information where we examine these effects, included here as Fig R7. As a reminder, the optimal mutation rate can be found by solving for θ using the following equation

$$\kappa = \frac{R}{P} e^{-\beta(\Delta\varepsilon_{\mathrm{rd}} - \Delta\varepsilon_{\mathrm{pd}} + \theta(l_r \Delta\Delta\varepsilon_{\mathrm{rd}} - l_p \Delta\Delta\varepsilon_{\mathrm{pd}}))} = 1. \tag{1}$$

Here, we can vary l_r and l_p in order to vary the size of the binding motif. Moreover, we can also alter the change in binding energy due to each mutation as a way of manipulating the information content of the binding motif. Simply put, if a binding motif has high information content, we would expect mutations to lead to a large effect on the total binding energy, i.e. $\Delta\Delta\varepsilon_{\mathrm{pd}}$ or $\Delta\Delta\varepsilon_{\mathrm{rd}}$ would be high. On the other hand, if a binding motif has low information content, we would expect mutations to have low impact on the total binding energy, i.e. $\Delta\Delta\varepsilon_{\mathrm{pd}}$ or $\Delta\Delta\varepsilon_{\mathrm{rd}}$ would be low. As shown in Fig R7, when we vary the length of the RNAP and repressor binding sites or when $\Delta\Delta\varepsilon_{\mathrm{pd}}$ and $\Delta\Delta\varepsilon_{\mathrm{rd}}$ are changed, the optimal mutation rates also stay within a range of below 0.4.

Importantly, since the authors frame the paper as a computational pipeline for experimental design of MPRAs, I would appreciate that the simulation results are translated into the more traditional terms of power analysis such as the AUC.

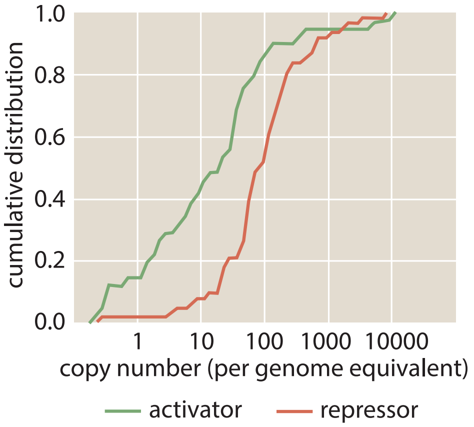

Figure R6: **Cumulative distribution of transcription factor copy numbers in *E. coli*.** Cumulative distribution of the copy numbers of both activators and repressors. The figure is adapted from Li et al. [26].

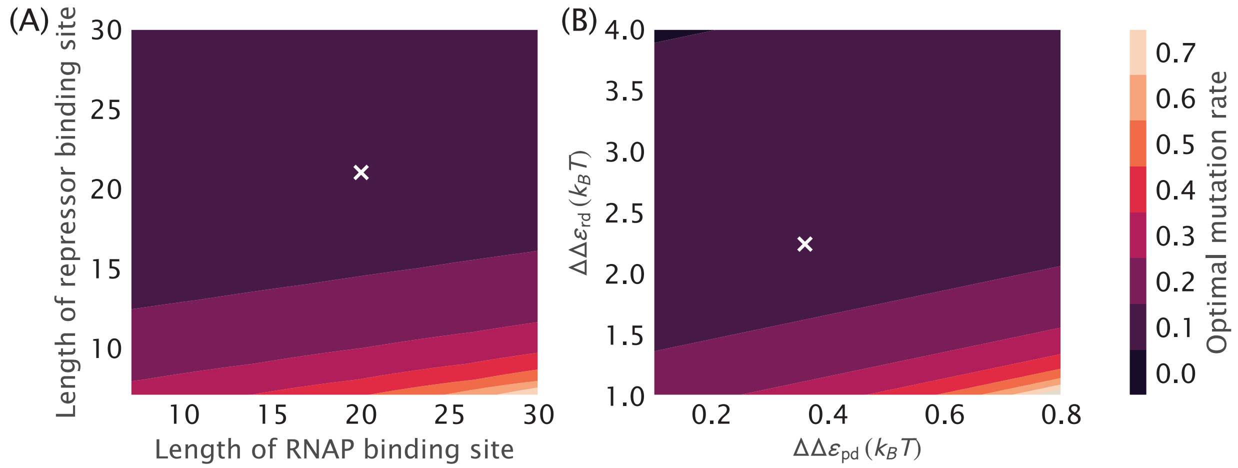

Figure R7: **Optimal mutation rate as a function of the size of the binding sites and information content of energy matrices.** Numerical solutions for the mutation rate calculated by finding the mutation rate that leads to equal Boltzmann weights between the RNAP bound state and the transcription factor bound state using Eq 1. Panel (A) varies the length of the RNAP and repressor binding sites. Panel (B) varies $\Delta\Delta\varepsilon_{\rm pd}$ and $\Delta\Delta\varepsilon_{\rm rd}$, which are the change in total binding energy given a single mutation at the RNAP binding site and at the repressor binding site. The white crosses mark the standard set of parameters: $l_r = l_p = 20$, $\Delta\Delta\varepsilon_{\rm pd} = 0.36\,k_{\rm B}T$ and $\Delta\Delta\varepsilon_{\rm rd} = 2.24\,k_{\rm B}T$.

Author's response: While we appreciate the suggestion, we worry we were not clear in our objectives. We are confused by this response. We do not frame our paper as simply a computational pipeline for the experimental design of MRPAs, though we certainly know that it is truly helpful in that regard since our two laboratories already use these ideas. The goal of this work is much broader than the reviewer is acknowledging. The goal is to understand how summary statistics that emerge from tools such as MPRAs depend upon all of the experimental and biological parameters. We are not in the business of critiquing the work of others. That said, many high throughput studies make no reference to how parameters such as transcription factor copy number, binding site strength, mutation rate, growth conditions, which inducers are used, etc.. will alter the measured output from MRPAs. Our goal in this paper is to derive insights into all of these parameters (and others). We would argue that

an AUC power analysis is not helpful in our work since we are not proposing a classifier, nor are we performing statistical testing in this work. It is not even clear what the reviewer wants us to subject to a statistical test. For example, as noted above, though we can look at information footprints from experiment and simulation side by side (as we have done after the useful suggestion of the reviewer), it is not useful to ask how well the two sets of footprints "agree" with each other. They are not meant to agree with each other since the whole point is that we do not know the parameters in the experimental situation - that is the whole point, we are trying to ultimately discover them, but to do so, we first need to discover the regulatory architecture itself.

As is the simulation results provoke interesting thoughts, but I do not see how a research can translate them into actionable advice on MPRA design and interpretation. The insights with respect to promoter architecture and TF-concentration as well as competition for binding sites also remain not more than interesting thought experiments. Moreover, they are highly dependent on the initial assumptions, such as the importance of the exact location. Also here, I do not see concrete advice on how these insights will be helpful to real life examples with unknown TF architectures. Can the simulations help to distinguish such scenarios?

Author's response: Again, we apologize that we have not made our objectives clear in our writing. We consider this work to be extremely practical since every section examines realistic scenarios that one can expect to encounter (an we have encountered) when performing MPRAs. The reviewer brings up both design and interpretation and we hope both here and in the detailed list below we might change their outlook. Firstly, on the question of design, in both of our laboratories these computational tools are already in use. There are big conceptual and financial questions involved in constructing MPRA libraries and the computations performed here have already had practical implications in our own work. We note that the results of our earlier work in *E. coli* are already including in RegulonDB [2, 28] and we suspect that if we do another 1000 genes, others will adopt identical or similar approaches.

We are confused by the reviewer's comments on interpretability, especially since the reviewer has not cited or provided any scholarly references to the literature of the field where anyone else has even tackled these questions. We are great admirers of many other works in the field (Kosuri [29, 30], Kinney [31], Palsson [32, 33, 34]); however, to the best of our knowledge, all of them are silent on the question of how MPRA results will inevitably depend upon variables such as mutation rate, transcription factor copy number, transcription factor binding strength, growth condition and transcription factor inducer, and whether the genes are expressed on plasmid constructs or integrated into the genome, etc. In previous works from our lab, we have demonstrated repeatedly and unambiguously how these variables systematically tune gene expression [3, 35, 22, 36, 37, 38, 39]. Every one of the papers we have just cited has a figure that shows how tuning some experimental or biological parameter systematically tunes gene expression. Hence, it is important for this field to understand how MPRA outputs depend upon those variables that are often hidden or completely unknown, and this work addresses exactly this point. If the reviewer knows differently, it would be most helpful to tell us what we have missed.

To elaborate on the points that we made above, below is a section-by-section explanation of how our analysis can be applied to MPRA design and interpretation. We also link here as we did in the previous round of comments to enormous datasets that depend critically upon exactly the kind of analysis performed in this paper (see our website for the new iteration of Reg-Seq and Appendix R1)

- Section 1.2 discusses how changing mutation rate and adding mutational biases affect the level of signals in information footprints. This section informs the reader on how to choose an appropriate mutation rate in the design of an MPRA library. In particular, we also propose an analytical framework to precisely calculate the optimal mutation rate. On the other hand, the analysis on

mutational biases is from the very practical consideration that in some systems, the methods for engineering MPRA libraries would lead to mutational biases. For example, as mentioned in our response to a previous comment, the Garcia lab is actively engaged in developing methods for introducing mutations at target sites in the *Drosophila* genome. As shown in Fig R10, *in vivo* mutagenesis using the somatic hypermutation enzyme AID from sea lamprey, which is currently the most efficient method of mutagenesis they have found for their studies, is highly biased towards C to T and G to A mutations [24]. Our simulations, which are shown in Fig 4(C) in the main text, take a look at exactly these mutational biases and help to establish expectations on how the information footprints would be affected when mutation biases are present. These are not thought experiments – they are representations of specific behaviors. The Garcia lab is explicitly using these ideas in the design of real world experiments – we find it troubling that the reviewer so easily dismisses work as "thought experiments" without even carefully performing the relevant background scholarship.

- Section 1.3 examines the noise in information footprints as a function of library size. This is very practical as library size needs to be carefully chosen in order to balance high signal-to-noise ratio to the cost of library synthesis. As shown in Fig R8, Ireland et al. has shown that in Reg-Seq, the reproducibility of downstream inference models increases as the size of the MPRA library increases [2]. Inspired by these experimental results, we wanted to very carefully examine how the library size alters the summary statistics. In *E. coli*, there are on the order of a few thousand promoters [30]. This means to achieve the goal of dissecting the regulatory architectures of every gene in the *E. coli* genome, we need an MPRA pipeline that can be used to dissect 10^3 promoters at a time. In previous iterations of Reg-Seq [2], we used a mutant library of 1,500 sequence variants for each promoter, where each sequence variant contains scattered random mutations. If we continue to use a library with 10^3 sequences for every promoter in *E. coli*, the total number of sequence variants would exceed 10^6. In our lab, mutant libraries are synthesized in the form of oligo pools. Fig R4 shows the cost of synthesizing different sizes of oligo pools with different oligo lengths. Here, we see that the cost of synthesizing an oligo pool with 10^6 oligos that are 212 bp long (the size of our oligo constructs) is around 60 thousand dollars, which is prohibitively expensive. Using our computational pipeline, we can estimate the minimum library size required to obtain summary statistics using libraries of different design, which will help us to cut down this cost.

- Section 2.1 explores the significance of changes to the copy number and the binding energies of the repressor. Again, we are confused by this reviewer's pronouncements that this is just a thought experiment. As shown in Fig R9, our previous work in both bacteria and flies demonstrates that these effects are huge and systematic. To the best of our knowledge, the field generally does not even acknowledge that MPRAs will depend upon uncontrolled variables such as transcription factor copy number. Recent proteomic studies (see Figure R6) [26, 27] give us hints as to the range of transcription factor copy numbers and the point of these calculations is to explore how MPRAs and the corresponding summary statistics will depend upon those copy numbers. As stated in the main text, here our results serve as a cautionary note for MPRA interpretation. Since weak binding or low transcription factor copy numbers can both drastically lower the signal, we cannot completely rule out the possibility that a binding site is still present just from the lack of signal in information footprints. Again, we are not in the business of critiquing previous work, but the reviewer must be aware that most MRPA studies do not even consider the question of how the results depend upon copy number.

- Section 2.2 takes a close look at how changing transcription factor copy number affects information footprints under different regulatory architectures. This is important because transcription factor knock-out and knock-down experiments are a useful way of verifying which transcription factors are bound at a given binding site. One's naïve expectations might be that once a transcription factor is knocked down, we would no longer observe a signal at the associated binding site.

However, when the knock-down method is leaky, we can still see appreciable signal at the binding site and the level of the signal depends on the regulatory logic. This helps us to correctly interpret the presence of signals when transcription factors are knocked down.

- Section 2.3 focuses on the competition between transcription factor binding sites. We would be deeply grateful if the reviewers were to consult our 2014 Cell paper by Brewster et al. which demonstrates that our results are anything but thought experiments [36], as seen in Fig R9. There, we systematically, rigorously and quantitatively explored exactly the titration effects that this reviewer erroneously claims are idle intellectual speculations. On the contrary, this is again extremely practical because the method for delivering MPRA libraries into cells may change the copy number of the transcription factor binding sites. For example, mutant libraries can either be integrated into the genome directly or delivered using plasmids. When the mutant library is integrated into the genome, the original copy number of the binding site would be preserved. On the other hand, when plasmid constructs are used, the copy number of transcription factor binding sites would depend on the plasmid copy number. In our current experimental endeavours, such as the one shown in Fig R11, we also see that the information footprints for some promoters contain different peaks when genome integration is used versus when plasmid constructs are used. We need to, as does the whole field, address the key question of how different the read-outs are expected to be when they are obtained from an experiment that uses genome integration and when they are obtained from an experiment that uses a plasmid system. This section investigates this point and informs the reader on which method of delivering the mutant library is appropriate and how to choose a plasmid with an appropriate copy number. Again, to the best of our knowledge we are the first and only people to address this question. Usual studies do not even acknowledge this could be an effect. How can this kind of careful analysis that is tied intimately and directly to experiments be referred to as "thought experiments?"

- Section 2.4 looks at inducer concentration. This is relevant because when MPRAs are performed, we often need to add the appropriate inducers to the media in order to activate the transcription factors of interest. This analysis performed in this section helps with the decision of an appropriate level of inducer concentration in the growth media. The caveat here is that we often do not know what the inducer is for a given transcription factor. In the experimental work in our own lab, we perform assays under 40 different growth conditions. However, even this vast array of growth conditions is not comprehensive. Again, we are puzzled by what the reviewer is referring to. In what sense is exploring how inducer concentration alters MPRA summary statistics a thought experiment? For an example of a very careful dissection of how inducer concentration systematically and quantitatively tunes the level of bacterial transcription, we point to our own earlier efforts at careful dissection of this effect [38].

- Section 3.1 to 3.3 feature a discussion on the various factors that could lead to noise in MPRAs. To annotate transcription factor binding sites from data, it is important that we are able to distinguish between signal and noise. In order to do this, we need to have a baseline understanding of how much noise is expected to arise from different sources. Our simulations provide exactly these insights.

We have edited the paper to make these practical implications clearer at the end of each results section. All in all, the goal of the simulations is the help the reader to choose appropriate experimental parameters and provide a baseline for what the MPRA output would look like in different scenarios. It is true that we prescribe a specific set of parameters and binding site locations in the examples provided in our analysis. However, as mentioned earlier, we have carefully chosen parameters that have been empirically measured and physiologically relevant in order to ensure the applicability of the insights generated from these analyses.

Finally, to answer the reviewer's question, the simulations would indeed help us to distinguish between different regulatory architectures. Again, our simulations establish expectations for what the footprints

would look like for different architectures. One of the current research directions that stemmed from this paper is to develop algorithms to automatically classify the regulatory architecture of a given promoter based on the measured information footprints, and these simulated datasets are extremely helpful as training data. This is outside of the scope of the current paper but we are excited to discuss this in our future works.

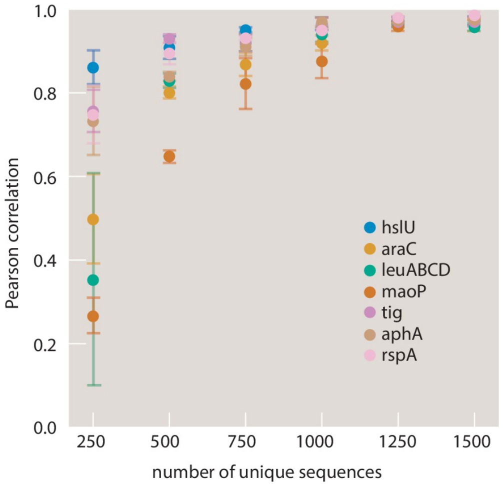

Figure R8: **Pearson correlation as a function of library size.** A the library size increases, the Pearson correlation between the energy matrix inference based on the full data set and the computationally subsampled data sets also increases, approaching 1.0, suggesting that the reproducibility of the energy matrix inference increases. The figure is adapted from Figure 1 in Appendix 1 of Ireland et al. [2].

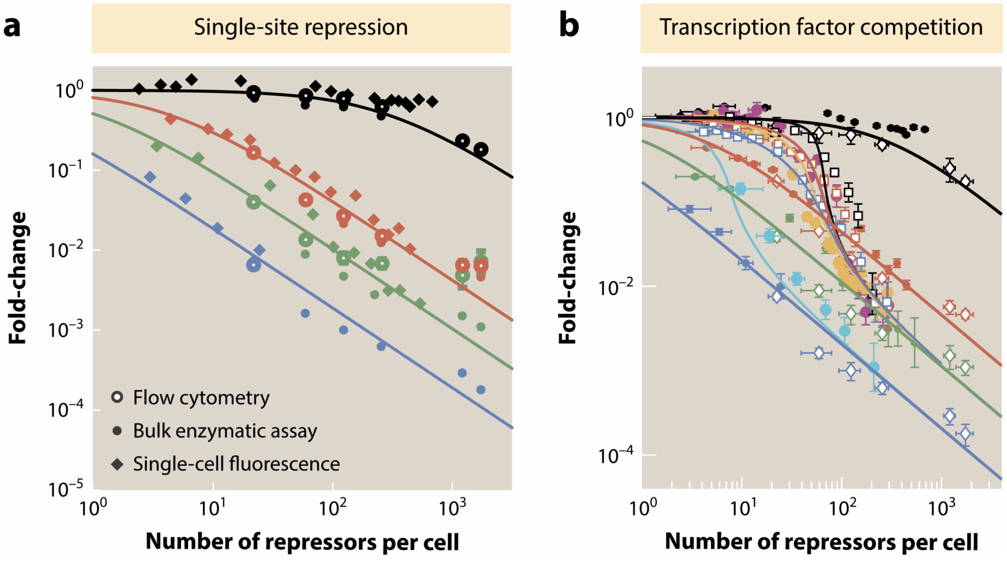

Figure R9: **Theory-experiment dialogue in simple repression.** The figure is taken from Phillips et al. [40]. The curves are parameter-free predictions of fold-change as a function of the number of repressors per cell. (a) Fold-change for simple repression with a single binding site. (b) Fold-change for simple repression while taking into account titration effects. The curves are parameter-free predictions before the measurements were even done.

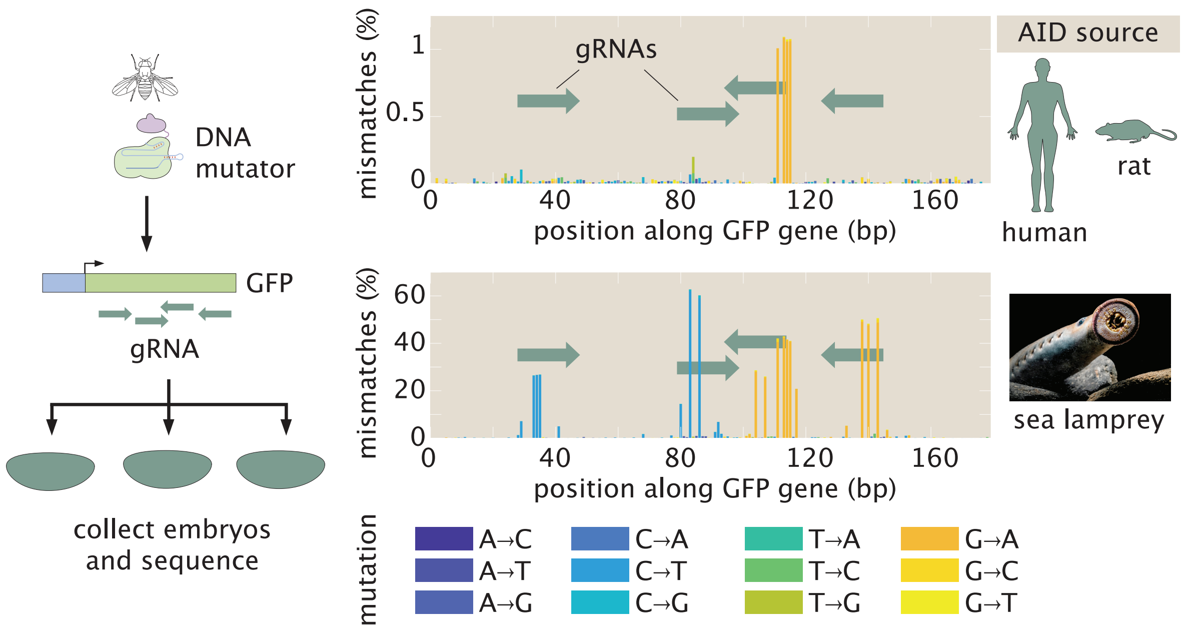

Figure R10: ***In vivo* mutagenesis in *Drosophila* introduces mutational biases.** To introduce mutations at target sites in *Drosophila* embryos, one method is to use a DNA mutator that introduces random mutations at sites targeted by gRNAs. A possible DNA mutator is the somatic hypermutation enzyme AID. The widely used version of AID originates from humans and rats; however, these AIDs have low mutation efficiency. In comparison, the AID from sea lamprey has much higher efficiency but still introduces mutations with high bias towards C to T and G to A mutations. The figure is adapted from Falo-Sanjuan et al. [24]. We argue that the use of models like these provided in our paper reveal how summary statistics from MPRAs will be altered by mutational biases. These are not thought experiments.

Adding the non-equilibrium model at the end is nice, but again I don't quite see the practical implications. If I understand correctly, only the simplest architecture is implemented and for some conditions the simpler and more flexible equilibrium model will suffice. Again a comparison of simulations to real life data would be helpful, to guide the user towards what model is appropriate.

Author's response: We are not sure if the reviewer saw the earlier round of comments and responses. The inclusion of the non-equilibrium example was an addition in response to an earlier reviewer comment to show that such an analysis is possible. We could of course repeat this analysis for every such architecture, but to do so is to write a whole new paper.

Similarly, we are not sure if this reviewer saw Appendix R1, which is a 300 page document containing our data for over 100 promoters in roughly 40 growth conditions. The whole point of this exercise is that these theoretical approaches are poised for synergy with these huge quantities of data. We agree with the reviewer that it would be informative to compare our simulations of non-equilibrium systems with experimental data. However, there is no known non-equilibrium transcription systems that have been intentionally studied in an MPRA, and so it is not possible for us to perform such a comparison at the moment. Nevertheless, the model developed in this paper can serve as a guide for experimentalists to infer potential out-of-equilibrium behavior. For instance, if a footprint resembling that of simple repression is observed under certain conditions in an activation regulatory system, this could suggest energy consumption.

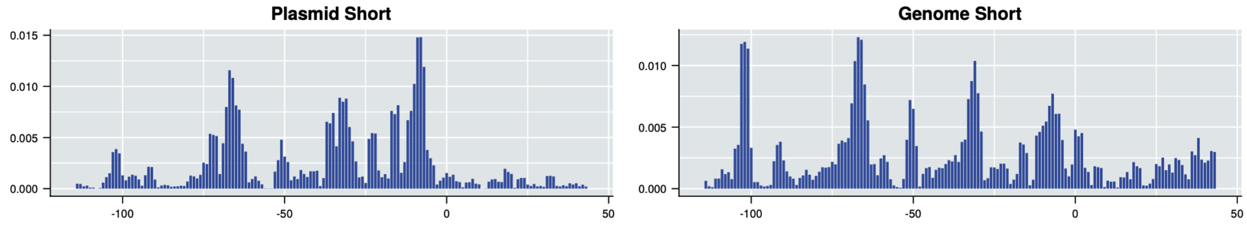

Figure R11: **Experimental information footprints obtained through a plasmid system and through genome integration.** For the rspA promoter, the peak at -100 base pair relative to the transcription start site appears in the footprint obtained through genome integration (right) but the peak is very weak in the footprint obtained through a plasmid system. NOte that both of these approaches are used experimentally and we are trying to systematically understand how that experimental choice alters the summary statistics from the data.

Furthermore, the paper is too long, it should be possible to shorten. Each chapter centres on a specific scenario and then repeats a similar train of logic. It should be possible to shorten and only describe the implications.

Author's response: We appreciate the reviewer's concern. To shorten the paper, we have moved a few parts of the main text to the Supplemental Information, including the analytical demonstration of hitchhiking effects, the states-and-weights diagram for a promoter with the double repression architecture, the explanation of the chemical potential approach for building thermodynamic models, the derivation of p_{bound} for an inducible repressor, and the derivation of p_{bound} for a promoter with broken detailed balance. We hope these changes make the paper cleaner and easier to digest.

Most importantly, the computational pipeline that I found on GitHub is a collection of jupyter notebooks that is rather sparsely annotated. If this is to be of use to other people more work is needed here. At an absolute minimum the functions provided in tregseq require a detailed description including I/O.

Author's response: We thank the reviewer for this very important suggestion. Indeed, we completely share the values represented by the reviewer's remarks. We have added markdown notes to explain each code cell in our jupyter notebooks and we also added more detailed documentation to our scripts to make the code more accessible.

In summary, given the limitations of the model both the introductory remarks and the discussion are vastly overstated. This paper could be of merit if a few results are clearly formulated and not buried between lengthy derivations and the overstated introduction and discussion. For most parts, this paper reads more like a review than the description of a computational pipeline as is claimed in the discussion. The authors need to decide on a focus and rewrite accordingly.

Author's response: We are keen to properly convey and highlight the main results of the paper. As mentioned in our response to an earlier comment, we have moved some of the derivations to the Supplemental Information in order to put more focus on our results. Nevertheless, we would greatly appreciate scholarship in this dialogue. What "model" is the reviewer referring to and in what sense are things overstated? It is hard to even know how to respond to such vague remarks. Moreover, as we have mentioned in our introduction, we are aware of the shortcomings of thermodynamic models and we discuss these shortcomings thoroughly in Appendix S1. Did the reviewer look at that appendix which was added as a result of the previous round of reviews? Furthermore, almost half

of our discussion is dedicated to addressing an array of limitations of thermodynamic models. This paper is not about promoting or celebrating thermodynamic models. It is about deeply understanding the strengths and limitations of high-throughput experimental approaches being used routinely in the study of transcription. The models we used here provide a actionable way to see in total detail how sequence relates to expression as a function of the key experimental and biological parameters in these assays.

On the subject of being "overstated", we urge the reviewer to critically examine some of the key tenets of the paper that we highlight in the introduction and discussion. First, is it true that in *E. coli* for more than half the promoters there is no regulatory knowledge as measured by the two principal databases EcoCyc and RegulonDB? Is it true that for roughly 30% of the proteins in *E. coli* that there functions are currently unknown as explained carefully by Palsson and coworkers [41]? The situation is worse in yeast and *Drosophila* for example, as evidenced by the corresponding databases and illustrated in Fig R12. Next, is it an overstatement to say that in previous MPRAs the role of both biological and experimental parameters are unknown? For a beautiful recent example of impressive forward progress on this question, we ask the reviewer to see the recent paper by Galagan et al. (for example their Figure 1) which attempts to deal with some of the huge regulatory uncertainty that attends the study of the *E. coli* genome [42]. Again, since one of us has spent more than a decade as a leader in the *Drosophila* field, we would expect that our statement that the same kind of regulatory challenges exist in the study of insect regulation is not an overstatement.

In this case, to the extent the reviewer is interested in helping us, it would have been useful to get some precise critiques or suggestions rather than the use of vague words like "overstated". In the previous round of reviews we gave lengthy and careful review of the use of thermodynamic models in prokaryotes and eukaryotes alike. Even as recently as this year a paper from several Stanford groups used such models as a jumping off point [12]. Second, the comment about reading like a review is not backed up by any scholarly citation. Again, as we pointed out in the previous round of reviews, neither this reviewer nor anyone else can or will show us examples of calculation of the summary statistics such as information footprints or expression shift matrices, of which our paper provides many. This field is full of examples of careful measurements using reporter assays with no attendant description of how mutation rate, plasmid vs chromosomal integration, transcription factor copy number or DNA binding strength (to name a few variables) are examined. Our goal was to provide a thorough, systematic and rigorous examination of all of the ways that MPRAs are influenced by experimental and biological parameters. That is a long list and we have tried to be thorough about it. We find it hard to understand why someone wants to critique us for being thorough.

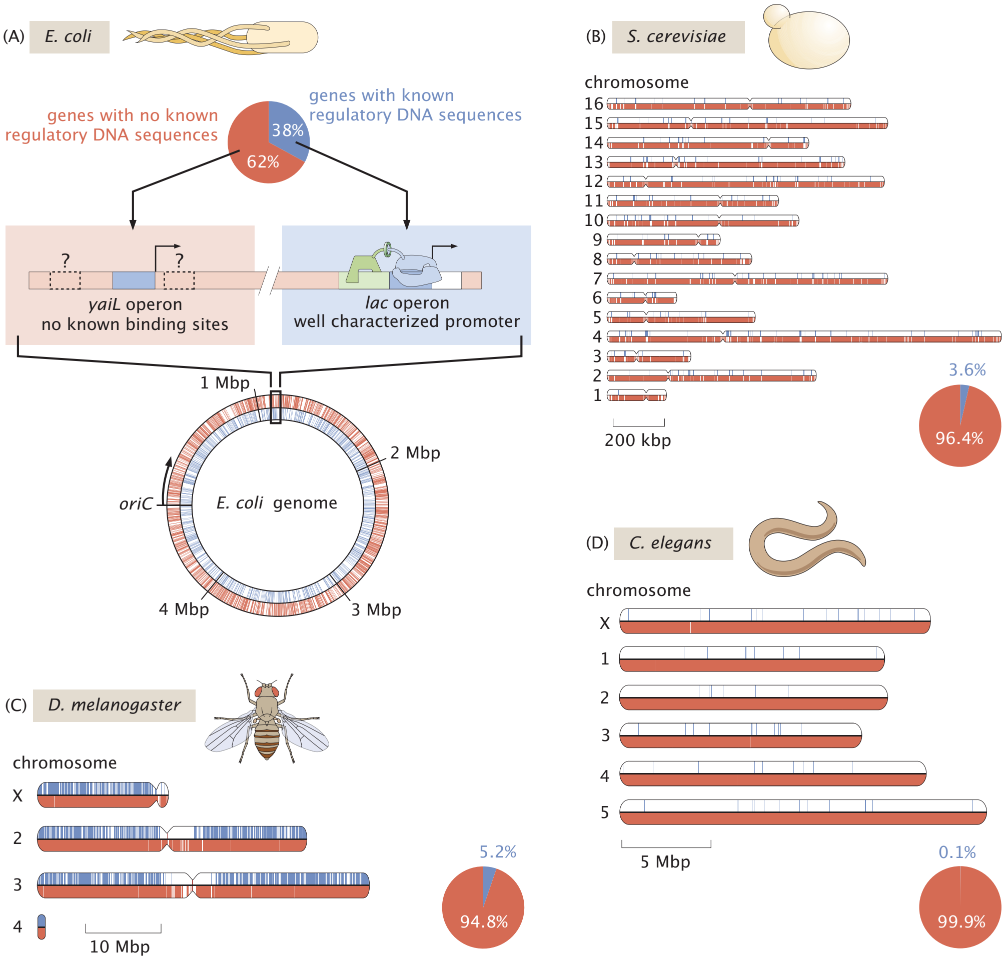

Figure R12: **Missing regulatory information in key model organisms.** (A) Missing regulatory information in the *E. coli* genome as reported in databases. According to current databases (as of 2016), there are 872 operons for which we have some knowledge of their regulatory architecture (shown in blue), while there are 1762 operons for which we currently have no regulatory knowledge (shown in red). (B) Missing regulatory information in the *Saccharomyces cerevisiae* genome. Roughly 96% of the genes have no regulatory annotation on the publicly available databases. (C) Missing regulatory information in the *Drosophila melanogaster* genome as reported on publicly available databases. Roughly 95% of the genes have no regulatory annotation. (D) Missing regulatory information in the *Caenorhabditis elegans* genome as presented in publicly available databases. Over 99% of the genes have no regulatory annotation. MPRAs provide an exciting opportunity to respond to this regulatory challenge. However, going from MPRA data to a full description of the regulatory architecture is a challenging task and the models used in this paper make it much easeier to understand the MPRA data and how it is altered by experimental and biological parameters.

Minor points

I find the title misleading. Even though a single cell is simulated, it is not intended for single cell MPRA analysis. Also the title should make it clear that the focus is on microbial systems not to mislead people like me who work on mammalian single cell genomics.

Author's response: We agree with the reviewer that the title may be confusing for the readers, and we have changed the title to "Deciphering regulatory architectures of bacterial promoters from synthetic expression patterns". We hope this change addresses the reviewer's concern.

Many of the appendices appear to be basic repetitions of what is already in the main paper, this makes it extremely difficult to find the relevant information. Again I could not find a real pipeline description.

Author's response: We are unsure of why the reviewer see the appendices as a repetition of what is in the main paper, as all of the appendices contain missing details that were not provided in the main text. More importantly, one of our fundamental beliefs in writing papers is that we want our readers to be able to reproduce every single item throughout the paper and thorough, detailed SIs are one approach to that challenge. We have reorganized our Supplemental Information to group related subsections, which will hopefully make it easier for the readers to find relevant information. We have also added sentences to our main text to make sure that each of the Supplemental Appendices is explicitly called out. We believe that given the format of PLOS Computational Biology, if the manuscript is published, the end of the paper will contain a list of all appendices, which should help the readers find the relevant information. We also show the list of all appendices at the end of our manuscript after the references. If someone doesn't want to read the SI material, they can simply ignore it. The paper stands on its own merits, but the thorough SI is intended for practitioners that are interested in every detail down to the last factor of 2. Again, we are puzzled by why anyone would want to critique someone else for attempting to be thorough and helpful to future readers. As readers, we certainly appreciate detailed and well organized SIs.

To the reviewer's other point, the pipeline is described in detail in Sec 1.1 and illustrated by Fig 1 in our paper. If the reviewer is looking for some other kind of pipeline description, we would appreciate it if the reviewer could explain what they have in mind. We note also, this paper is not about presenting a pipeline. It is about providing a rigorous theory of the experiment to help us and everyone else to understand how MPRA results depend upon biological and experimental parameters.

References

1. Mahdavi SD, Salmon GL, Daghlian P, Garcia HG, and Phillips R. Flexibility and sensitivity in gene regulation out of equilibrium. Proc. Natl. Acad. Sci. U. S. A. 2024 Nov; 121:e2411395121
2. Ireland WT, Beeler SM, Flores-Bautista E, McCarty NS, Röschinger T, Belliveau NM, Sweredoski MJ, Moradian A, Kinney JB, and Phillips R. Deciphering the regulatory genome of *Escherichia coli*, one hundred promoters at a time. eLife 2020 Sep; 9:e55308
3. Jones DL, Brewster RC, and Phillips R. Promoter architecture dictates cell-to-cell variability in gene expression. Science 2014 Dec; 346:1533–6
4. Segal E, Raveh-Sadka T, Schroeder M, Unnerstall U, and Gaul U. Predicting expression patterns from regulatory sequence in Drosophila segmentation. Nature 2008; 451:535–40
5. Eck E, Liu J, Kazemzadeh-Atoufi M, Ghoreishi S, Blythe SA, and Garcia HG. Quantitative dissection of transcription in development yields evidence for transcription factor-driven chromatin accessibility. Elife 2020; 9:e56429
6. White MA, Parker DS, Barolo S, and Cohen BA. A model of spatially restricted transcription in opposing gradients of activators and repressors. Mol Syst Biol 2012; 8:614
7. Park J, Estrada J, Johnson G, Vincent BJ, Ricci-Tam C, Bragdon MD, Shulgina Y, Cha A, Wunderlich Z, Gunawardena J, and DePace AH. Dissecting the sharp response of a canonical developmental enhancer reveals multiple sources of cooperativity. eLife 2019 Jun; 8:e41266

8. Kim YJ, Rhee K, Liu J, Jeammet S, Turner MA, Small SJ, and Garcia HG. Predictive modeling reveals that higher-order cooperativity drives transcriptional repression in a synthetic developmental enhancer. Elife 2022; 11:e73395

9. Kuang Y, Pyo A, Eafergan N, Cain B, Gutzwiller LM, Axelrod O, Gagliani EK, Weirauch MT, Kopan R, Kovall RA, Sprinzak D, and Gebelein B. Enhancers with cooperative Notch binding sites are more resistant to regulation by the Hairless co-repressor. PLoS Genet 2021; 17:e1009039

10. He X, Samee MA, Blatti C, and Sinha S. Thermodynamics-based models of transcriptional regulation by enhancers: the roles of synergistic activation, cooperative binding and short-range repression. PLoS Comput Biol 2010; 6

11. Fakhouri WD, Ay A, Sayal R, Dresch J, Dayringer E, and Arnosti DN. Deciphering a transcriptional regulatory code: modeling short-range repression in the *Drosophila* embryo. Mol Syst Biol 2010; 6:341

12. Doughty BR, Hinks MM, Schaepe JM, Marinov GK, Thurm AR, Rios-Martinez C, Parks BE, Tan Y, Marklund E, Dubocanin D, Bintu L, and Greenleaf WJ. Single-molecule chromatin configurations link transcription factor binding to expression in human cells. bioRxiv 2024 Feb :2024.02.02.578660

13. Gertz J, Siggia ED, and Cohen BA. Analysis of combinatorial cis-regulation in synthetic and genomic promoters. Nature 2009; 457:215–8

14. Raveh-Sadka T, Levo M, Shabi U, Shany B, Keren L, Lotan-Pompan M, Zeevi D, Sharon E, Weinberger A, and Segal E. Manipulating nucleosome disfavoring sequences allows fine-tune regulation of gene expression in yeast. Nat Genet 2012; 44:743–50

15. Chickarmane V, Enver T, and Peterson C. Computational modeling of the hematopoietic erythroid-myeloid switch reveals insights into cooperativity, priming, and irreversibility. PLoS Comput Biol 2009; 5:e1000268

16. Benos PV, Bulyk ML, and Stormo GD. Additivity in protein-DNA interactions: how good an approximation is it? Nucleic Acids Res. 2002 Oct; 30:4442–51

17. Takeda Y, Sarai A, and Rivera VM. Analysis of the sequence-specific interactions between Cro repressor and operator DNA by systematic base substitution experiments. Proc. Natl. Acad. Sci. U. S. A. 1989 Jan; 86:439–43

18. Hippel PH von and Berg OG. On the specificity of DNA-protein interactions. Proc. Natl. Acad. Sci. U. S. A. 1986 Mar; 83:1608–12

19. Le DD, Shimko TC, Aditham AK, Keys AM, Longwell SA, Orenstein Y, and Fordyce PM. Comprehensive, high-resolution binding energy landscapes reveal context dependencies of transcription factor binding. Proc. Natl. Acad. Sci. U. S. A. 2018 Apr; 115:E3702–E3711

20. Maerkl SJ and Quake SR. A systems approach to measuring the binding energy landscapes of transcription factors. Science 2007 Jan; 315:233–7

21. Garcia HG, Sanchez A, Boedicker JQ, Osborne M, Gelles J, Kondev J, and Phillips R. Operator sequence alters gene expression independently of transcription factor occupancy in bacteria. Cell Rep. 2012 Jul; 2:150–61

22. Johnson S, Lindén M, and Phillips R. Sequence dependence of transcription factor-mediated DNA looping. Nucleic Acids Res. 2012 Sep; 40:7728–38

23. Melnikov A, Murugan A, Zhang X, Tesileanu T, Wang L, Rogov P, Feizi S, Gnirke A, Callan Jr CG, Kinney JB, Kellis M, Lander ES, and Mikkelsen TS. Systematic dissection and optimization of inducible enhancers in human cells using a massively parallel reporter assay. Nat. Biotechnol. 2012 Feb; 30:271–7

24. Falo-Sanjuan J, Diaz-Tirado Y, Turner MA, Davis J, Medrano C, Haines J, McKenna J, Eisen MB, and Garcia HG. Targeted mutagenesis of specific genomic DNA sequences in animals for the in vivo generation of variant libraries. bioRxivorg 2024 Jun :2024.06.10.598328

25. Eck E, Liu J, Kazemzadeh-Atoufi M, Ghoreishi S, Blythe SA, and Garcia HG. Quantitative dissection of transcription in development yields evidence for transcription-factor-driven chromatin accessibility. Elife 2020 Oct; 9

26. Li GW, Burkhardt D, Gross C, and Weissman JS. Quantifying absolute protein synthesis rates reveals principles underlying allocation of cellular resources. Cell 2014 Apr; 157:624–35

27. Schmidt A, Kochanowski K, Vedelaar S, Ahrné E, Volkmer B, Callipo L, Knoops K, Bauer M, Aebersold R, and Heinemann M. The quantitative and condition-dependent *Escherichia coli* proteome. Nat. Biotechnol. 2016 Jan; 34:104–10

28. Belliveau NM, Barnes SL, Ireland WT, Jones DL, Sweredoski MJ, Moradian A, Hess S, Kinney JB, and Phillips R. Systematic approach for dissecting the molecular mechanisms of transcriptional regulation in bacteria. Proc. Natl. Acad. Sci. U. S. A. 2018 May; 115:E4796–E4805

29. Urtecho G, Tripp AD, Insigne KD, Kim H, and Kosuri S. Systematic Dissection of Sequence Elements Controlling σ70 Promoters Using a Genomically Encoded Multiplexed Reporter Assay in *Escherichia coli*. Biochemistry 2019 Mar; 58:1539–51

30. Urtecho G, Insigne KD, Tripp AD, Brinck MS, Lubock NB, Acree C, Kim H, Chan T, and Kosuri S. Genome-wide functional characterization of *Escherichia coli* promoters and sequence elements encoding their regulation. eLife 2023 Dec

31. Kinney JB, Murugan A, Callan Jr CG, and Cox EC. Using deep sequencing to characterize the biophysical mechanism of a transcriptional regulatory sequence. Proc. Natl. Acad. Sci. U. S. A. 2010 May; 107:9158–63

32. Sastry AV, Gao Y, Szubin R, Hefner Y, Xu S, Kim D, Choudhary KS, Yang L, King ZA, and Palsson BO. The *Escherichia coli* transcriptome mostly consists of independently regulated modules. Nat. Commun. 2019 Dec; 10:5536

33. Rychel K, Decker K, Sastry AV, Phaneuf PV, Poudel S, and Palsson BO. iModulonDB: a knowledgebase of microbial transcriptional regulation derived from machine learning. Nucleic Acids Res. 2021 Jan; 49:D112–D120

34. Qiu S, Wan X, Liang Y, Lamoureux CR, Akbari A, Palsson BO, and Zielinski DC. Inferred regulons are consistent with regulator binding sequences in *E. coli*. PLoS Comput. Biol. 2024 Jan; 20:e1011824

35. Garcia HG and Phillips R. Quantitative dissection of the simple repression input-output function. Proc. Natl. Acad. Sci. U. S. A. 2011 Jul; 108:12173–8

36. Brewster RC, Weinert FM, Garcia HG, Song D, Rydenfelt M, and Phillips R. The transcription factor titration effect dictates level of gene expression. Cell 2014 Mar; 156:1312–23

37. Barnes SL, Belliveau NM, Ireland WT, Kinney JB, and Phillips R. Mapping DNA sequence to transcription factor binding energy in vivo. PLoS Comput. Biol. 2019 Feb; 15:e1006226

38. Razo-Mejia M, Barnes SL, Belliveau NM, Chure G, Einav T, Lewis M, and Phillips R. Tuning Transcriptional Regulation through Signaling: A Predictive Theory of Allosteric Induction. Cell Syst 2018 Apr; 6:456–469.e10

39. Brewster RC, Jones DL, and Phillips R. Tuning promoter strength through RNA polymerase binding site design in *Escherichia coli*. PLoS Comput. Biol. 2012 Dec; 8:e1002811

40. Phillips R, Belliveau NM, Chure G, Garcia HG, Razo-Mejia M, and Scholes C. Figure 1 Theory Meets Figure 2 Experiments in the Study of Gene Expression. Annu. Rev. Biophys. 2019 May; 48:121–63

41. Ghatak S, King ZA, Sastry A, and Palsson BO. The y-ome defines the 35% of *Escherichia coli* genes that lack experimental evidence of function. Nucleic Acids Res. 2019 Mar; 47:2446–54

42. Lally P, Gómez-Romero L, Tierrafría VH, Aquino P, Rioualen C, Zhang X, Kim S, Baniulyte G, Plitnick J, Smith C, Babu M, Collado-Vides J, Wade JT, and Galagan JE. Predictive biophysical neural network modeling of a compendium of in vivo transcription factor DNA binding profiles for *Escherichia coli*. bioRxivorg 2024 May
